# An N-heterocyclic germylene with a versatile metal-binding pocket: insights into heterodinuclear bonding and reactivity†

**DOI:** 10.1039/d5sc02751a

**Published:** 2025-06-05

**Authors:** Errikos Kounalis, Rik Sieben, Léon Witteman, Martin Lutz, Marc-Etienne Moret, Daniël L. J. Broere

**Affiliations:** a Organic Chemistry and Catalysis, Institute for Sustainable and Circular Chemistry, Faculty of Science, Utrecht University Universiteitsweg 99 3584 CG Utrecht The Netherlands m.moret@uu.nl d.l.j.broere@uu.nl; b Structural Biochemistry Bijvoet Centre for Biomolecular Research, Faculty of Science, Utrecht University Universiteitsweg 99 3584 CG Utrecht The Netherlands

## Abstract

We report the synthesis, isolation, and characterisation of an N-heterocyclic germylene (NHGe) derived from a two-electron reduced Mg-synthon of the redox-active ^dipp^NBA ligand. This NHGe features a vacant binding pocket capable of coordinating various metals, which enables the formation of heterobimetallic Ge–Zn and Ge–Mg complexes. Electronic structure calculations reveal that the Ge–Zn interactions are weak, whilst the interactions between the metals found for the heterodinuclear Ge–Mg complex are stronger. These findings highlight how the nature of the Ge–M interactions adapts to the electron density requirements of the metal occupying the redox-active binding pocket flanking the Ge(ii) centre. Notably, the Ge–Mg complex undergoes a ‘Metallo-Diels–Alder’ reaction with unsaturated C–C bonds, activating these bonds over the Ge centre and the ligand backbone – a transformation that does not proceed without Mg. This provides a compelling example of indirect cooperativity, where the Mg centre electronically stabilises a quadruply reduced ligand, upon which Ge engages in metal–ligand cooperative activation of C–C unsaturated bonds.

## Abstract 2

We report the synthesis, isolation, and characterisation of an N-heterocyclic germylene (NHGe), which features a vacant binding pocket that enables the synthesis of heterobimetallic Ge–Zn and Ge–Mg complexes.

## Graphical abstract

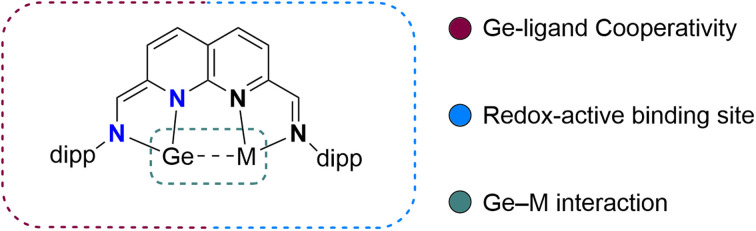

## Introduction

**Scheme 1:**
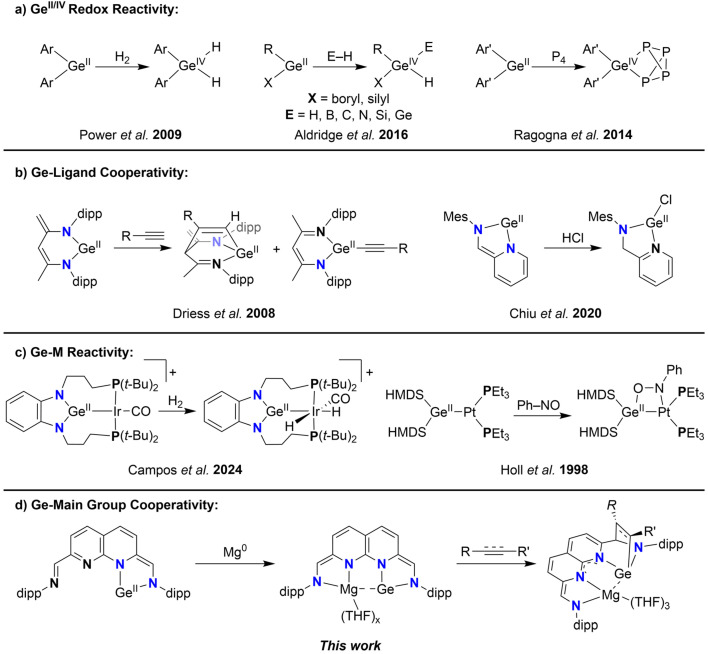
(a–c) The various types of reactivity modes described for germylenes and (d) the Ge-main group cooperativity described in this work.

Germylenes, ambiphilic Ge(ii)-complexes that bear an empty p-orbital and a Ge-centred lone pair, have attracted significant attention as promising tools in main-group catalysis due to their unique electronic structure and reactivity patterns.^1,2^ Much like N-heterocyclic carbenes (NHCs) form a substantial subclass within the lightest of the tetrylenes, N-heterocyclic germylenes (NHGes) represent a similarly significant subclass of germylenes, distinguished by analogous bonding and electronic features.^3^ The ring-systems of these NHGes exhibit different degrees of unsaturation^4^ and ring-size, spanning from 4- to 7-membered motifs.^5–10^ Additionally, decoration of the N-donor with bulky substituents results in kinetic stabilisation of the reactive Ge(ii) centre. The reactivity of these centres is largely due to the reduced state of Ge(ii), where the Ge(ii/iv) redox couple facilitates the activation of bonds like C–H and H–H *via* oxidative addition (Scheme 1a).^11–13^ This allows Ge(ii) to exhibit reactivity patterns that closely parallel those of transition metals (TMs). Redox-neutral bond activation can also take place through Ge-ligand cooperativity. In these cases the bonds are activated ditopically over the Ge(ii) centre and the ligand backbone, retaining the divalent state on Ge. Some examples of this type of reactivity feature ylide-like Ge-complexes bearing diketiminate (NacNac) ligands or Ge-complexes ligated by iminopyridines, allowing for the activation of a plethora of bonds (Scheme 1b).^8,9,14–21^

The ambiphilic nature of germylenes also renders them excellent ligands for transition metals, with examples spanning the whole d-block.^22–24^ The versatility of the potential Ge–M manifolds that can be synthesised also translates to a versatility in the loci of reactivity. The reactivity of these systems can either be localised on one of the two components of the Ge–M manifold, or be ditopic and spread over both components (Scheme 1c).^25–28^ In an interesting example by Cui *et al.*, a bis-germylene was synthesised bearing a redox-active naphthyridine diimine ligand,^29,30^ which could act as donor of up to six electrons.^31^ Redox-active ligands (RALs) can act as electron reservoirs, storing electrons and eliminating the need to access energetically unfavourable oxidation states at the bound metal centre.^32^ This results in a flattening of the energy landscape of chemical transformations, with the metals acting as a conduit for the electrons. A compelling example is the reported four-electron reactivity of a iminopyridine–germylene complex, where the Ge(ii) centre is oxidised to Ge(iv) while also acting as conduit for the electrons stored in the RAL.^18^ This electronic flexibility of RALs has also been leveraged in main group complexes to impart bond activation reactivity typically associated with transition metals.^33^ For example, a bis-Al(ii) complex bound to redox-active diimine ligands can mediate the four-electron cleavage of the N=N bond in azo-compounds, with the electrons required being provided by both oxidation of the Al(ii) centres and the RALs.^34^ This promising avenue has led to the isolation of a multitude of RAL-based complexes of main group metals such as Mg,^35,36^ Al,^37–42^ Ga,^43–47^ Si,^35^ and Ge.^18,31,48^ In contrast to the well-reported cooperative reactivity of germylene–TM complexes, examples reporting cooperative transformations such as ditopic bond activation or synergistic redox activity have, to the best of our knowledge, not been extended to germylene-main group combinations.^49^

We hypothesised that the naphthyridine diimine scaffold would enable us to integrate the previously described strategies within a single, versatile germylene scaffold. Instead of introducing two Ge(ii) centres in the dinucleating naphthyridine scaffold, introduction of only one Ge(ii) centre would leave a vacant redox-active pocket for a main group metal (or TM) to bind in close proximity to the germylene and potentially facilitate the envisioned cooperative reactivity (Scheme 1d). Here we report the synthesis, characterisation and electronic structure of such an NHGe. We describe the binding of Zn and Mg to the vacant site and detail the electronic structures of these heterodinuclear complexes, focussing on the interaction between the Ge(ii) centre with the metal in close proximity. Lastly, we detail the activation of substrates containing C–C multiple bonds by the Ge–Mg complex, where the Mg centre plays a vital role in electronically stabilising a quadruply reduced ligand, whilst Ge engages in metal–ligand cooperative activation of C–C unsaturated bonds.

## Results and discussion

**Scheme 2:**
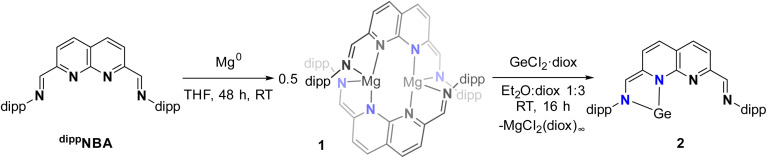
Syntheses of 1 and 2 starting from the redox-active ^dipp^NBA ligand.

Reacting the ^dipp^NBA ligand with 1 equiv. of Mg^0^ turnings in THF (Scheme 2) led to the slow formation of a dark red solution from which 1 was isolated as a red solid in 73% yield. Analysis of the ^1^H-NMR spectrum revealed full conversion of the ligand, along with several resonances consistent with the two halves of the naphthyridine motif being equivalent (see ESI Section 1.2†). The singlet at *δ* = 6.61 ppm, attributed to the methine linker, and the two doublets at *δ* = 5.70 and 4.99 ppm (^3^*J*_H,H_ = 7.6 Hz), attributed to the naphthyridine protons, are all significantly shifted upfield, consistent with a reduced character of the naphthyridine backbone.^29^ A single septet for the methine protons of the dipp i-Pr groups at *δ* = 3.43 ppm was observed and two doublets at *δ* = 1.53 and 0.98 ppm (^3^*J*_H,H_ = 6.8 Hz) were observed for the magnetically inequivalent methyl group protons of the dipp substituents.

**Fig. 1:**
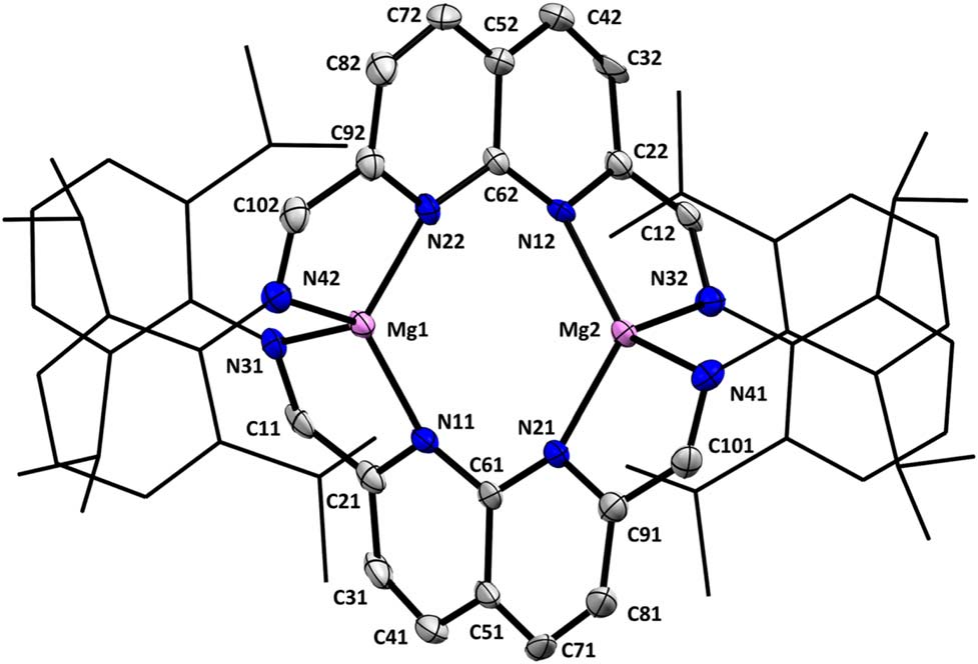
Displacement ellipsoid plot of 1 at 50% probability. Hydrogen atoms and minor disorder components are omitted for clarity. The dipp substituents are depicted as wireframe for clarity.

Single crystals suitable for analysis by X-ray diffraction were grown by layering a saturated THF solution of 1 with pentane. The dimeric nature of 1 was revealed in the solid-state structure (Fig. 1), with the two naphthyridine motifs being twisted at 67.9(10)° (dihedral angle between the two planes defined by N11–C61–N21 and N12–C62–N22, see ESI Fig. S74†) with respect to each other to accommodate a Mg centre on each side. The two-electron reduction of each ligand is evident through the bond metrics, showing a contraction of the C_methine_–C_napy_ and an elongation of the C_methine_–N_dipp_ bonds compared to the free ligand (see ESI Table S3†). The C_methine_–C_napy_ bond lengths on either side of both naphthyridine moieties are identical within error, consistent with the delocalised nature of the ligand-centred reduction, which agrees with the ^1^H-NMR spectrum and is also found in related two-electron reduced naphthyridine systems.^29,31^

With the reduced ^dipp^NBA-synthon 1 in hand, we set out to install the germylene in the reduced naphthyridine pocket. Treatment of a 3 : 1 1,4-dioxane : Et_2_O solution of 1 with 2 equiv. of the Ge(ii) precursor GeCl_2_·diox (diox = 1,4-dioxane) resulted in the formation of a white precipitate, presumably polymeric [MgCl_2_(diox)_2_]_∞_,^50^ and compound 2, which was isolated as a red solid in 68% yield. ^1^H- and ^13^C{^1^H}-NMR analysis of 2 in C_6_D_6_ revealed a resonance count consistent with the loss of the previously observed symmetry in 1 (see ESI Section 1.3†). Two sets of naphthyridine resonances are observed, one at *δ* = 8.14 and 7.02 ppm (^3^*J*_H,H_ = 7.7 Hz), and another further upfield at *δ* = 6.55 and 6.06 ppm (^3^*J*_H,H_ = 9.1 Hz). Additionally, two singlets are observed at *δ* = 8.40 and 6.79 ppm, which we attribute to the protons attached to the pendant methine carbons. Combined, these observations are consistent with two distinct binding pockets in 2. One pocket retains its aromaticity, as evidenced by the more downfield set of naphthyridine protons and the singlet at *δ* = 8.40 ppm, which is consistent with the aldimine character of the methine proton. In contrast, the other binding pocket exhibits a more reduced character. This is evident from the upfield shift of the second set of naphthyridine resonances, indicating dearomatisation of the naphthyridine pocket. This dearomatisation is also reflected by the upfield shift of the resonance of the methine proton to *δ* = 6.79 ppm, resulting from a decrease of its aldimine character. The magnetic inequivalence observed for the dipp –CH_3_ resonances in 1 is absent in the non-reduced pocket of 2, where a single doublet is observed at *δ* = 1.18 ppm (^3^*J*_H,H_ = 6.8 Hz). The opposite is true for the reduced pocket of 2, which is apparent through the presence of two slightly overlapping doublets at *δ* = 1.12 and 1.11 ppm. This magnetic inequivalence can be indicative of constrained rotation around the aniline C–N bond on the NMR timescale,^51^ which we ascribe to the introduction of Ge into the reduced pocket.

The high solubility of 2 in various apolar solvents precluded us from obtaining crystals suitable for analysis by X-ray diffraction. To probe whether 2 exists as a monomer or dimer (like 1) in solution, we resorted to convection-corrected Diffusion Ordered Spectroscopy (ccDOSY-NMR). This showed that 2 is monomeric in solution (see ESI Section 1.4†), similar to a recently reported structurally related NHGe reported by Cui *et al.*^52^

**Scheme 3:**
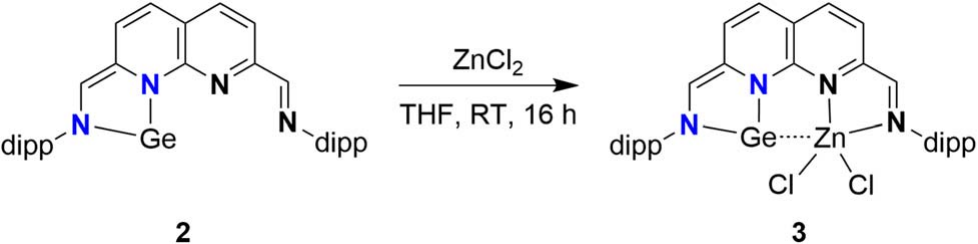
Complexation of ZnCl_2_ to the empty pocket of 2, yielding 3.

We subsequently explored the introduction of metals into the empty binding pocket of 2. The addition of an equimolar amount of ZnCl_2_ to a THF solution of 2 yielded a dark red/purple solution (Scheme 3) from which 3 was isolated in 68% yield. Analysis of the ^1^H-NMR spectrum (THF-*d*_8_, see ESI Section 1.5†) revealed two singlets, integrating to one proton each, at *δ* = 8.25 and 6.97 ppm, consistent with an aldimine and a methine proton. These chemical shifts are in agreement with one reduced binding pocket and one pocket retaining its iminopyridine character. Remarkably, the most downfield naphthyridine resonance out of the four is observed as a broad resonance lacking multiplicity rather than the expected doublet. A similar effect is observed for one of the two methine resonances of one of the dipp substituents at *δ* = 3.15 ppm. These observations are indicative of a dynamic change in speciation, with exchange being in the intermediate/fast regime on the timescale of the NMR experiments. In line with this interpretation, sharpening of the broad resonances in the ^1^H spectrum is observed upon recording the spectra at higher temperatures (see ESI Fig. S26–28†). Similarly, peak broadening and decoalescence of peaks is observed upon cooling the sample in the spectrometer in increments to 193 K (see ESI Fig. S23–25†). Interestingly, upon removal of the THF solvent, 3 changes colour to blue (see ESI Fig. S30†). This colour is observed for the solid-state, as well as in the solution-state for aromatic solvents and dioxane. Analysis of the ^1^H-NMR spectrum of 3 in C_6_D_6_ (see ESI Fig. S22†) revealed sharper resonances, suggesting that the observed fluxionality is likely induced by reversible THF coordination to the Zn centre, potentially followed by dissociation of the N-dipp sidearm.^51^

**Fig. 2:**
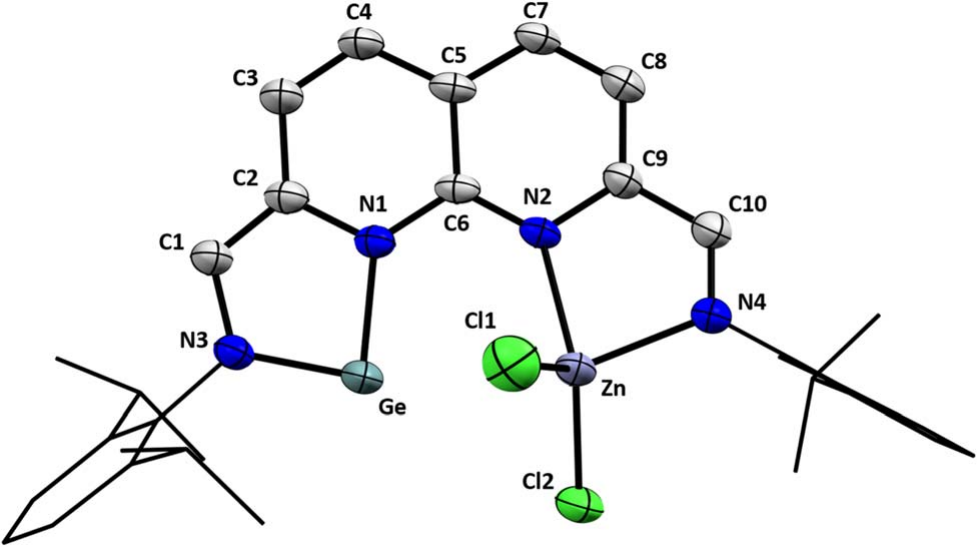
Displacement ellipsoid plot of 3 at 50% probability. Co-crystallised 1,4-dioxane molecules, hydrogen atoms and disordered components are omitted for clarity. The dipp substituents are depicted as wireframe for clarity.

Single crystals suitable for X-ray diffraction were grown by slow vapour diffusion of pentane into a saturated 1,4-dioxane solution of 3. The introduction of ZnCl_2_ to the empty pocket next to the NHGe motif was confirmed in the solid-state structure of 3 (Fig. 2). The N1–Ge–N3 bond angle of 84.15(17)° falls within the typical range for 5-membered NHGes.^53^ The Ge–Zn distance of 3.1110(8) Å lies between the sum of their covalent and their van der Waals radii (2.42 and 4.68 Å respectively),^54^ albeit appreciably larger than reported Ge–Zn distances.^55^ The environment around the Zn centre is best described as tetrahedral with a distortion due to the proximity of Ge. For the Ge-side of the molecule, the bond metrics are consistent with C–N single bonds (C1–N3 = 1.378(6) Å, C2–N1 = 1.410(6) Å) and the presence of a C=C bond located outside of the naphthyridine ring system (C1–C2 = 1.361(7) Å). These features are in agreement with the two-electron reduction of that naphthyridine pocket. The alternating single and double C–C bonds in the respective ring of the naphthyridine motif confirm the two-electron reduction. This is in stark contrast to the other ring, where C–C bonds of more uniform length are observed (see ESI Table S4†). Additionally, the bond metrics consistent with a C=N double bond (C10–N4 = 1.283(6) Å), and a C–C single bond external to the naphthyridine ring system (C9–C10 = 1.452(7) Å) further substantiate the α-diimine character of the pocket coordinating the Zn centre. The Ge⋯Cl distances (Ge⋯Cl1 = 3.527(2) Å, Ge⋯Cl2 = 3.5584(16) Å) are too long to assign any meaningful bonding interaction between these two atom pairs.^56^

**Scheme 4:**
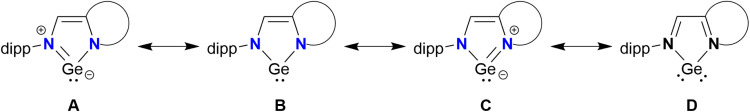
The described resonance structures of the NHGe motif found in complexes 2 and 3.

To gain insight into the electronic structure of the NHGe motif in 2 and 3, and the extent of Ge–Zn interactions in the latter, Density Functional Theory (DFT) and Natural Bond Orbitals (NBO) calculations were performed.^57–62^ The bond metrics of the geometry-optimised structure in the gas phase of 2 are in agreement with our proposed two-electron reduction of the naphthyridine pocket that coordinates the Ge(ii) centre (for more details, see ESI Section 2.5 and Table S1†). In the gas-phase optimised geometry of 3 (see ESI Section 2.7†), the calculated Ge–Zn distance is significantly shorter than the experimental distance (2.861 Å *vs.* 3.1110(8) Å). This difference in the Ge–Zn distance remains even when the calculations are performed without any empirical dispersion (2.868 Å, see ESI Section 2.8†). Because distance can be an important factor when investigating the extent of electronic interactions, we ran calculations on both the freely optimised gas-phase structure of 3 (a) and a structure where the Ge–Zn distance was fixed at the distance obtained from the solid-state structure (b, see ESI Section 2.9†). It is worth mentioning that both 3a and 3b underestimate one of the Ge⋯Cl distances by approx. 0.5 Å. Analysis of the frontier Kohn Sham orbitals of 2 revealed a delocalised bonding interaction spanning the N–Ge–N motif of the NHGe in the HOMO, along with antibonding interactions between Ge and the two N-donors in the LUMO and LUMO+1 orbitals (see ESI Fig. S61†). Using NBO analysis, we further explored the electronic structure of 2, 3a, and 3b. The partial dearomatisation of the naphthyridine backbone and the presence of a C=C bond located outside of the naphthyridine ring system (on the Ge side) were both observed in the Lewis structure on which the NBO calculations of 2, 3a, and 3b converged. According to the calculations, the π-bonding interaction along the N–Ge–N motif is best described as a 3-centre-4-electron (3c4e) hyperbond (see ESI Fig. S62†), which is in agreement with the reported electronic structure of related 5-membered N-heterocyclic tetrylenes.^63^ Structurally this can best be visualised as the superposition of the neutral germylene resonance structure (B) and the two ylide structures (A, C) depicted in Scheme 4. For 5-membered NHGe motifs like the one found in 2 and 3, an additional resonance structure has been proposed based on spectroscopic and reactivity studies: the so-called ‘chelated atom’ structure (Scheme 4, D).^18,64^ Based on the calculated bond metrics of the NHGe motif in both complexes, the contribution of this resonance structure – a formally Ge(0) centre stabilised by a diazabutadiene fragment – appears minor. Nonetheless, we envision that germylone-like reactivity of 2 and 3 is possible, based on the reported four-electron reactivity of a structurally similar iminopyridine–germylene complex, where the Ge centre is oxidised to Ge(iv) while also acting as conduit for the electrons stored in the redox-active ligand.^18^ Germanium being one of the heavier tetrels, the germylene lone pair of 2 resides in an orbital of mostly s-character (87%, 13% p-character, ESI Fig. S62c†), due to poor mixing of the s- and p-orbitals and hence lack of hybridisation.

**Fig. 3:**
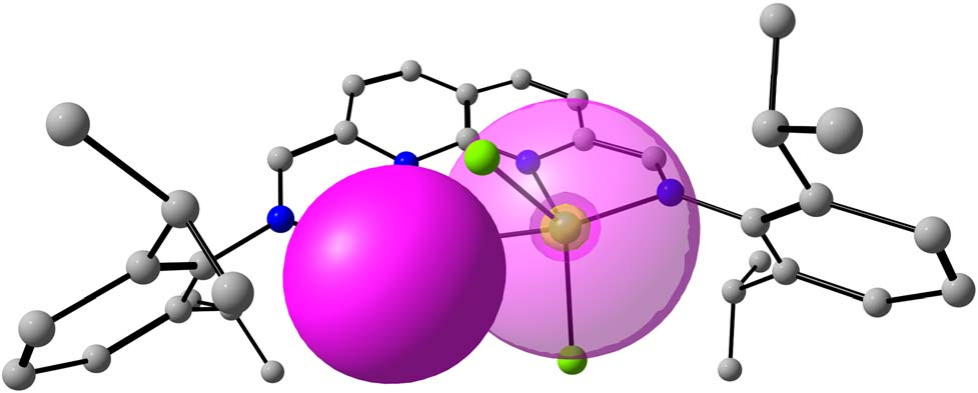
Overlap of the Ge-based lone pair donor NBO (filled) with the Zn-based acceptor NBO (translucent) of 3b, where the Ge–Zn distance was kept frozen to the value obtained from the solid-state structure. 2nd order pertubation analysis revealed that the Ge–Zn interactions are weak.

Similarly, the Ge lone pair of 3a and 3b resides in a spherically diffuse orbital of mostly s-character (90%). Second-order perturbation analysis revealed a delocalisation energy of 15.2 kcal mol^−1^ from the Ge lone pair donor NBO of 3a to a Zn-based acceptor NBO of s-character (>99%). The same interaction for 3b (Fig. 3) has a delocalisation energy of 6.7 kcal mol^−1^, consistent with the larger Ge–Zn separation and the resulting decrease in overlap between the donor and acceptor NBOs. Both interactions are small compared to the calculated interactions found between Zn and the N-donors (32–40 kcal mol^−1^) and Zn and the Cl-donors (68–90 kcal mol^−1^). Additionally, the calculations do not reveal any additional interactions from Zn to Ge.

**Scheme 5:**
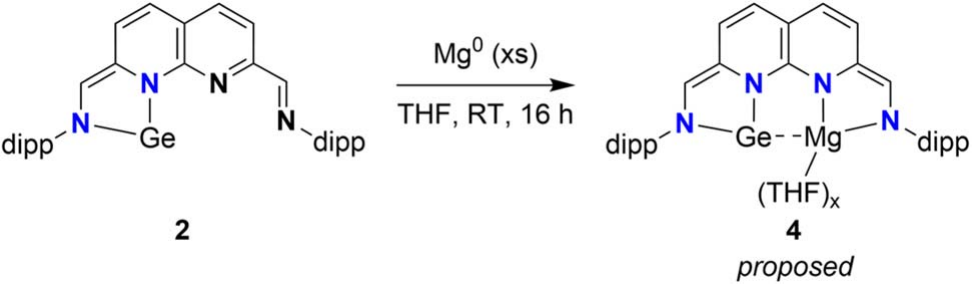
Reduction of red 2 with Mg^0^, resulting in the formation of dark green 4.

We subsequently investigated if the remaining binding pocket could be reduced. Treatment of a THF solution of 2 with a slight excess (2.5 equiv.) of fine mesh (325) Mg^0^ powder results in the formation of a dark green solution of 4, a highly reactive compound that decomposes upon complete removal of the solvent (Scheme 5). ^1^H-NMR analysis of 4 in THF-*d*_8_ (see ESI Section 1.6†) revealed four equally integrating naphthyridine resonances in the range of *δ* = 6.27–5.04 ppm. In contrast to the spectroscopic data of 2, no methine resonance with aldimine character was observed, with the two singlets attributed to the methine resonances being found at an upfield chemical shift of *δ* = 6.50 and 5.37 ppm. Combined, these observations are consistent with the two-electron reduction of the previously empty pocket (and formal four-electron reduction of the ligand), likely with a Mg centre being bound in a similar fashion as to a monomer of 1. The observed decomposition upon complete removal of the solvent implies that THF molecules are bound to 4. Combined with our NMR-spectroscopic observations, we propose a structure for 4 as depicted in Scheme 5.

**Scheme 6:**
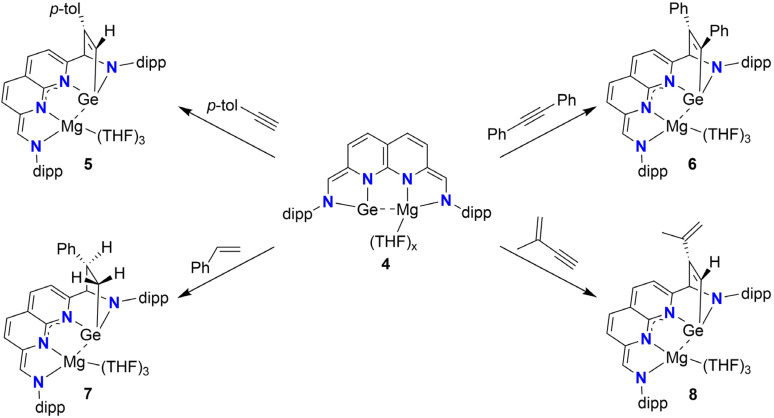
Syntheses of adducts 5–8 out of 4, with the unsaturated C–C bonds being activated through Ge-ligand cooperativity over the C_methine_–Ge vector.

Whilst the cooperative reactivity of germylene–transition metal complexes is well-documented,^22,25^ analogous transformations involving main-group partners have remained elusive. We therefore turned our attention to investigating whether 4 could enable (cooperative) bond activation. The addition of *p*-tolylacetylene to a THF solution of 4 resulted in the formation of a green solution within 5 minutes (Scheme 6, top-left). ^1^H-NMR analysis of the mixture in THF-*d*_8_ (see ESI Section 1.7†) revealed full conversion of 4 to a new non-symmetric species 5.

The four doublets attributed to the naphthyridine protons are found relatively upfield (*δ* = 5.54–4.71 ppm) and the chemical shift of the two singlets attributed to the sidearm protons (*δ* = 5.57 and 5.07 ppm) is more consistent with assignment to an enamide character of these protons over an aldimine character. The presence of two coupling doublets at *δ* = 7.27 and 6.97 ppm (^3^*J*_H,H_ = 8.1 Hz, 2H each) and a singlet at *δ* = 2.23 ppm (3H), are indicative of one equivalent of *p*-tolylacetylene being bound. We attribute the singlet at *δ* = 7.38 (^1^H) ppm to the terminal proton of the former acetylene fragment. Through a combination of 2D NMR experiments we were able to confirm the addition of one of the unsaturated C–C bonds of *p*-tolylacetylene over the Ge centre and the methine, resulting in a bicyclo[2,2,1]heptane motif (Scheme 6).

**Fig. 4:**
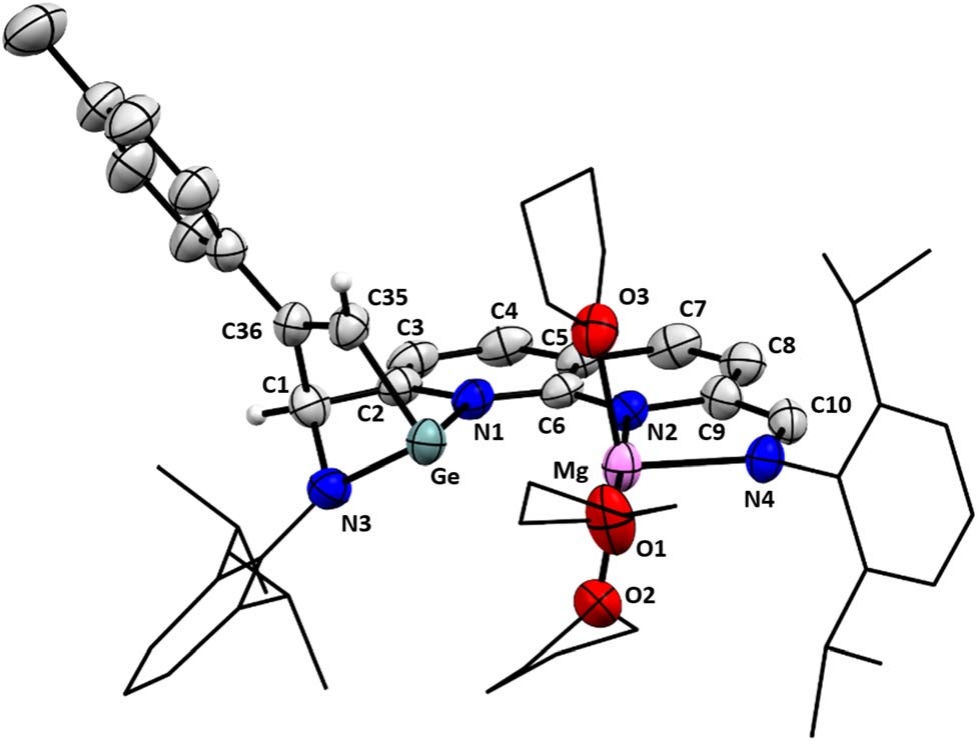
Displacement ellipsoid plot of 5 at 50% probability. Hydrogen atoms and minor disorder components are omitted for clarity. The dipp substituents and the carbon backbones of the THF molecules are depicted as wireframe for clarity.

Storing a saturated THF/MTBE solution of 5 at −40 °C yielded single crystals suitable for analysis X-ray diffraction (Fig. 4). The solid state-structure of 5 confirms the presence of a Ge and a Mg centre bound to the ^dipp^NBA ligand. The solid-state structure also corroborates the formation of the bicyclo[2,2,1]heptane motif, consistent with our spectroscopic observations. The Ge–Mg distance of 2.9115(15) Å lies well within the sum of their van der Waals radii (4.80 Å) and outside of the sum of their covalent radii (2.62 Å).^54^ Additionally, the distance is significantly longer compared to other reported Ge–Mg distances.^65^ These observations are consistent with a dative Ge–Mg bond being present rather than a covalent bond (see below). The geometry at the Ge centre is distorted tetrahedral and the geometry around the Mg centre is distorted octahedral, with three meridionally binding THF ligands completing the coordination sphere. The C9–C10 bond length of 1.380(6) Å is consistent with a double bond, indicative of the two-electron reduction of the binding pocket coordinated to Mg(ii). On the Ge side of the complex, the C1–C2 (1.511(6) Å) and C35–C36 (1.342(6) Å) bond lengths are consistent with single and double bond character, respectively. The addition of one of the unsaturated C–C bonds of the acetylene moiety over the NHGe motif can best be described as a formal [4 + 2] cycloaddition or ‘Metallo-Diels–Alder’ reaction of *p*-tolylacetylene with the NHGe motif from resonance structure C (Scheme 4).^66^

The bond metrics of the naphthyridine motif (see also ESI Table S5†) are consistent with localised double bonds between C2–C3 (1.349(6) Å), C4–C5 (1.379(6) Å), and C7–C8 (1.368(6) Å) and provide a rationale for the relatively upfield chemical shift of the naphthyridine protons in solution. Combined with the short C6–N1 and C6–N2 distances (1.354(5) and 1.332(5) Å, respectively), the metrics point towards delocalisation of one anionic charge over the N1–C6–N2 motif.

The computationally derived bond metrics of geometry-optimised 4d (4 with 3 THF molecules bound to Mg) and 5 (see ESI Fig. S73†) further corroborated our observations from NMR-spectroscopy and the X-ray crystal structure. The alternating single/double C–C bonds observed throughout the naphthyridine backbone found for 4d are consistent with a four-electron reduced ligand featuring two bis-anionic NN pockets. The calculated bond metrics of 5 match the experimental bond metrics well (see ESI Fig. S71†) and support the description of a two-electron reduced naphthyridine-imine ligand with a delocalised anionic charge over the N1–C6–N2 motif.

To get a better understanding of the Ge–Mg interactions found for 4 and 5, electronic structure calculations were performed (for a detailed description see ESI Section 2.10†). NBO analysis indicates that the Ge–Mg interaction in 4, bound to 0–3 THF molecules (structures 4a–4d, respectively), is best described by donation from the Ge lone pair residing in a spherically diffuse donor NBO (s-character = 79.9–86.8%) to a Mg-based acceptor NBO of high s-character (>95%). These dative interactions are consistently stronger than the respective contributions from the N- and O-donors and decrease slightly upon increasing the coordination number of the Mg centre. The dative interaction is equally strong after the observed addition of *p*-tolylacetylene over the NHGe in 5, albeit with a small decrease of the s-character of the Ge lone pair (73.7%) and delocalisation energy. These results demonstrate that there is a strong, dative Ge–Mg interaction present in both 4 and 5. This is in contrast with our observations for Ge–Zn complex 3 and highlights the versatility of the germylene centre in 2, with the extent of Ge–M dative interactions varying as a function of the required electron density on the metal centre that occupies the redox-active binding pocket.

We subsequently set out to explore the scope of C–C unsaturated bonds we could activate with 4. Treating a THF solution of 4 with 1 equiv. of diphenylacetylene at ambient temperatures resulted in full conversion of 4 to 6 (Scheme 6, top-right). Analysis of the ^1^H- and ^13^C{^1^H}-NMR spectra (THF-*d*_8_, see ESI Section 1.8†) revealed similar spectra to 5. The characteristic ^13^C{^1^H} chemical shift of the bridgehead methine (*δ* = 85.4 ppm) and a ^1^H–^13^C HMBC cross peak between its attached proton and a carbon resonance at *δ* = 165.8 ppm (attributed to the alkyne carbon added to the methine carbon of the NHGe motif) confirm the addition of the alkyne triple bond over the NHGe.

To explore if C=C bonds could also be activated, we treated a THF solution of 4 with styrene (Scheme 6, bottom-left). Gratifyingly, full conversion of 4 was observed to yield blue 7. Extensive 1D and 2D NMR analysis (THF-*d*_8_, see ESI Section 1.9†) showed that 7 is formed as a single diastereomer with the phenyl group pointing towards the naphthyridine motif as depicted in Scheme 6. The diastereomer with the phenyl group pointing towards the N-dipp motif was not observed, an observation we attribute to steric clashing.

Finally, to investigate the chemoselectivity of 4 towards alkenes or alkynes, we treated 4 with 2-methylbut-1-en-3-yne, a conjugated enyne (Scheme 6, bottom-right). Examination of the ^1^H-NMR spectrum (THF-*d*_8_, see ESI Section 1.10†) revealed full conversion of 4, yielding 8. Similar to 5, a downfield singlet resonance at *δ* = 7.15 ppm that shows a ^1^H–^13^C ASAP-HMQC cross-peak with a carbon resonance at *δ* = 152.3 ppm (*vs. δ* = 7.38 and 150.5 ppm found for 5), are indicative of preferential addition of the alkyne moiety to the NHGe over the alkene moiety. Consistent with this assignment, two singlet resonances in the alkene region of chemical shift are found, which are bound to the same carbon atom (*δ* = 110.3, confirmed through ^1^H–^13^C ASAP-HMQC NMR), and were assigned to the terminal alkene protons.

Similar “Metallo”-Diels–Alder reactions of (phenyl)acetylene with an ylide-like NHGe have been reported by the Driess group, yielding the respective bicyclo[2,2,2]octane adducts alongside the terminal C–H activation products (Scheme 1b).^15^ We propose that the latter is not observed for our system due to lower basicity of the methine positions of 4 compared to the diketiminate-based ylide. For the Driess system, reaction of the NHGe with diphenylacetylene does not take place, even at elevated temperatures, which was attributed to increased steric strain of the system.^15^ We propose that the discrepancy in observed reactivity between the two systems is influenced by two factors: the decreased steric strain around the Ge centre due to the presence of only one dipp substituent in 4 and the higher nucleophilicity of the methine carbon of the NHGe.

The reactions depicted in Scheme 6 yield bicyclo[2,2,1]heptane adducts where the unsaturated substrates are added over the Ge and methine position in one NN binding pocket, while leaving the other NN pocket containing Mg unaltered. Hence, we performed analogous reactions between 2 – containing the same germylene motif but lacking said Mg – with an equivalent of the same unsaturated substrates. However, even at prolonged reaction times or reflux conditions no conversion of 2 was observed.^67^ The observed discrepancy in reactivity of 2 and 4 is also reproduced computationally (see ESI Table S2†), with addition of *p*-tolylacetylene over the NHGe motif in 2 being endergonic by 0.7 kcal mol^−1^, whereas the same reaction for 4d was found to be exergonic by 25.5 kcal mol^−1^ and thereby suggesting that this difference is thermodynamic in nature. We hypothesise that this difference stems from the different oxidation states of the ^dipp^NBA ligand. In 2, the ligand is reduced by two electrons, with aromaticity being retained in one of the naphthyridine rings. In contrast, no aromaticity is retained in the formally quadruply reduced ligand of 4. The combined experimental and computational data do not indicate that aromaticity is gained upon cycloaddition,^68^ but do suggest increased delocalisation of charge over the naphthyridine N–C–N motif (see ESI Fig. S73†). We therefore propose that the majority of thermodynamic driving force originates from decreased electronic repulsion within the quadruply reduced ^dipp^NBA ligand upon cycloaddition.

The difference in reactivity between 2 and 4 highlights the crucial role of the Mg centre flanking the Ge centre, despite it not being directly involved in bond breaking or electron transfer events. We argue that the Mg centre electronically stabilises the four-electron reduced ligand sufficiently to allow for this reactive state to be accessed.^69^ This provides a compelling example of indirect cooperativity, where the Mg centre electronically stabilises the quadruply reduced ligand, upon which the germylene engages in metal–ligand cooperative activation of C–C unsaturated bonds.

## Conclusion

In conclusion, we report the isolation, synthesis, and characterisation of NHGe 2, featuring a vacant, redox-active binding pocket that is able to bind metals in close proximity to the Ge centre. This was demonstrated by the isolation of Ge–Zn (3) and Ge–Mg (4) complexes, with the versatile redox-active binding pocket accessing different oxidation states. We envision that complex 4 presents a promising entry point into germylene–(transition) metal chemistry, through transmetalation of the Mg centre in the bis-anionic binding site with various metal precursors. Electronic structure calculations reveal that the extent of the Ge–M dative interactions can vary as a function of the required electron density on the metal centre that occupies the redox-active binding pocket, with the Ge–Zn interaction in 3 being weak, whilst strong dative Ge–Mg interactions are found for 4. The latter engages in ‘Metallo-Diels–Alder’ reactions with a multitude of substrates containing C–C unsaturated bonds, activating the C–C bond over Ge and the ligand backbone. The same reaction does not proceed prior to two-electron reduction of the vacant binding site by Mg^0^. This provides a well-defined example of indirect cooperativity, where the Mg centre electronically stabilises the quadruply reduced ligand, upon which the germylene engages in metal–ligand cooperative activation of C–C unsaturated bonds. Combined, these findings not only expand the understanding of germylene reactivity but also open new avenues for designing systems that leverage cooperative interactions between germylenes and proximal metals for chemical transformations.

## Author contributions

Synthesis and characterisation were done by E. K. with support from R. S. and L. W. All crystallographic measurements and the corresponding data refinement was performed by M. L. Computations were done by E. K. Project design, supervision and oversight were done by M. E. M. and D. L. J. B. Funding acquisition and administration were done by D. L. J. B. The original draft was written by E. K. and reviewing and editing was done by M. E. M. and D. L. J. B. with contributions from all authors.

## Conflicts of interest

There are no conflicts to declare.

## Supplementary Material

SC-OLF-D5SC02751A-s001

SC-OLF-D5SC02751A-s002

## Data Availability

The data supporting this article have been included as part of the ESI,† the raw data can be accessed through the Yoda repository, DOI: https://doi.org/10.24416/UU01-FJ03BR. CCDC 2427530–2427533 contain the supplementary crystallographic data for this paper. These data can be obtained free of charge from The Cambridge Crystallographic Data Centre *via*www.ccdc.cam.ac.uk/data_request/cif.
