# Supplementary material for: An N-heterocyclic germylene with a versatile metal-binding pocket: insights into heterodinuclear bonding and reactivity
Source: Chem Sci. 2025 Jun 5;16(27):12613–22. doi: 10.1039/d5sc02751a (PMC12161148; doi:10.1039/d5sc02751a)
Supplement: SC-016-D5SC02751A-s001 [file SC-016-D5SC02751A-s001.pdf]

Supplementary Information (SI) for Chemical Science.
This journal is © The Royal Society of Chemistry 2025

SUPPORTING INFORMATION FOR

An N-Heterocyclic Germylene with a Versatile Metal-Binding Pocket: Insights into Heterodinuclear Bonding and Reactivity

Errikos Kounalis,[a] Rik Sieben,[a] Léon Witteman,[a] Martin Lutz,[b] Marc-Etienne Moret,*[a] Daniël L. J. Broere*[a]

[a] Organic Chemistry & Catalysis, Institute for Sustainable and Circular Chemistry, Utrecht University, Universiteitsweg 99, 3584 CG, Utrecht, The Netherlands

[b] Structural Biochemistry, Bijvoet Centre for Biomolecular Research, Faculty of Science, Utrecht University, Universiteitsweg 99, 3584 CG, Utrecht, The Netherlands

***Corresponding Authors**
m.moret@uu.nl, d.l.j.broere@uu.nl

Contents

1. Experimental methods: ...S3
1.1 General Considerations: ...S3
1.2 Synthesis of (^{dipp}NBA*Mg)$_2$ (1): ...S4
1.3 Synthesis of ^{dipp}NBA*Ge (2): ...S8
1.4 ccDOSY-NMR Analysis of ^{dipp}NBA*Ge: ...S12
1.5 Synthesis of ^{dipp}NBA*GeZnCl$_2$ (3): ...S15
1.6 Synthesis of ^{dipp}NBA**GeMg·(THF)$_x$ (4): ...S23
1.7 Synthesis of ^{dipp}NBA*GeMg·*p*-tolylacetylene (5): ...S27
1.8 Synthesis of ^{dipp}NBA*GeMg·diphenylacetylene (6): ...S31
1.9 Synthesis of ^{dipp}NBA*GeMg·styrene (7): ...S35
1.10 Synthesis of ^{dipp}NBA*GeMg·2-methylbut-1-en-3-yne (8): ...S40
2. Computational Methods: ...S44
2.1 General Considerations: ...S44
2.2 Example Input File for Geometry Optimisation: ...S44
2.3 Example Input File for NBO Calculations: ...S44
2.4 Example Input File for SP Calculations: ...S45
2.5 Electronic Structure of 2: ...S45
2.6 XYZ Coordinates of 2: ...S47
2.7 XYZ Coordinates of 3a: ...S49
2.8 XYZ Coordinates of 3a Without Empirical Dispersion: ...S51
2.9 XYZ Coordinates of 3b: ...S53
2.10 Electronic Structure of Complexes 4a-4d: ...S55
2.11 XYZ Coordinates of 4a: ...S58
2.12 XYZ Coordinates of 4b: ...S60
2.13 XYZ Coordinates of 4c: ...S62
2.14 XYZ Coordinates of 4d: ...S64
2.15 XYZ Coordinates of 5: ...S67
2.16 XYZ Coordinates of 2·*p*-tolylacetylene: ...S72
2.17 XYZ Coordinates of *p*-tolylacetylene: ...S74
3. Crystallographic Information: ...S75
3.1 X-ray Crystal Structure Determination of ^{dipp}NBA: ...S75
3.2 X-ray Crystal Structure Determination of (^{dipp}NBA*Mg)$_2$ (1): ...S75
3.3 X-ray Crystal Structure Determination of ^{dipp}NBA*GeZnCl$_2$ (3): ...S76
3.4 X-ray Crystal Structure Determination of ^{dipp}NBA*GeMg·*p*-tolylacetylene (5): ...S77
4. References: ...S79

1. Experimental methods:

1.1 General Considerations:

All manipulations were performed under inert atmosphere using standard Schlenk techniques or inside a N_2-filled MBraun UNILABplus or N_2-filled MBraun MB200B glovebox using anhydrous solvents and reagents, unless stated otherwise. Glassware was dried under vacuum at 130 °C before use. Solvents were collected from a MBraun MB-SPS-800 solvent purification system and stored over 4 Å molecular sieves. THF was distilled from a purple ketyl solution and degassed by sparging with N_2. All solvents were degassed by sparging with N_2. Deuterated solvents were obtained from Cambridge Isotope Laboratories, degassed by 3 freeze-pump-thaw cycles followed by backfilling with N_2 and stored over molecular sieves. All commercial reagents were obtained from Sigma Aldrich, Strem, Fischer Scientific or Acros and used without further purification. NMR data was recorded on an Agilent MRF400 equipped with an oneNMR probe and Optima Tune system, on a Varian VNMR-S-400 equipped with an AutoX probe and Agilent ProTune probe tuning accessory or on a 400 MHz Jeol EZCL G system with a HFX probe. Spectra were recorded at 298 K and chemical shifts (δ) are given in ppm referenced to (one of) the residual solvent peak (7.16 for C_6D_6, 3.58 for THF-d_8, 2.09 for toluene-d_8). All resonances in ^{13}C-NMR were referenced to the solvent. IR spectra were recorded on a FT-IR PerkinElmer Spectrum Two^{TM} spectrophotometer equipped with an ATR-probe. Elemental analyses were performed by MEDAC ltd. in the United Kingdom. **^{dipp}NBA** and 1,8-naphthyridine-2,7-dicarboxaldehyde were synthesised according to literature procedures.[1,2] Single crystals suitable for analysis by X-Ray diffraction of **^{dipp}NBA** were grown at -40 °C from a saturated THF/hexane solution.

1.2 Synthesis of (^{dipp}**NBA***Mg)$_2$ (**1**):

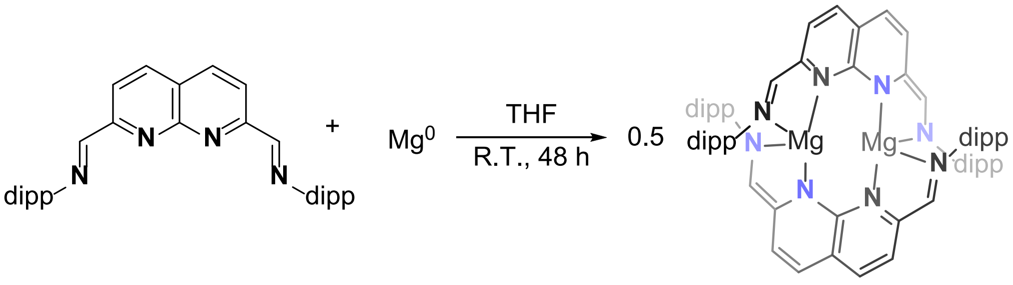

1

A solution of ^{dipp}**NBA** (149.9 mg, 297.0 µmol, 1.05 equiv) in THF (6 mL) was added to a suspension of Mg^0 turnings (6.9 mg, 283.8 µmol, 1 equiv) in THF (1.5 mL). The mixture was allowed to stir for 48 h at ambient temperature and resulted in a dark red mixture. The mixture was filtrated and afterwards all volatiles were removed under vacuum, yielding (^{dipp}**NBA***Mg)$_2$ (**1**) as a dark red solid. (115 mg, 73% yield).

Single crystals of **1** suitable for analysis by X-Ray diffraction were grown by layering a saturated THF solution of **1** with pentane.

^{1}H-NMR (400 MHz, C_6D_6, 298 K): δ 7.09-7.04 (m, 4H), 7.02 (m, 8H), 6.61 (s, 4H), 5.71 (d, $^3J_{H,H}$ = 7.6 Hz, 4H), 4.99 (d, $^3J_{H,H}$ = 7.6 Hz, 4H), 3.43 (sept, $^3J_{H,H}$ = 6.8 Hz, 8H), 1.53 (d, $^3J_{H,H}$ = 6.8 Hz, 24H), 0.98 (d, $^3J_{H,H}$ = 6.8 Hz, 24H).

^{13}C{^{1}H}-NMR (100 MHz, C_6D_6, 298 K): δ 169.23, 152.22, 147.00, 141.91, 141.24, 140.20, 131.69, 124.65, 124.45, 113.20, 30.0, 24.7, 24.3.

ATR-IR (cm^{-1}): 3059 (m), 2951 (s), 2926 (m), 2868 (m), 1573 (m), 1542 (w), 1493 (w), 1429 (m), 1397 (m), 1333 (m), 1299 (m), 1233 (w), 1204 (m), 1178 (m), 1106 (w), 1055 (m), 1034 (m).

Elemental Analysis: The reactive nature of this compound precluded obtaining satisfactory elemental analysis.

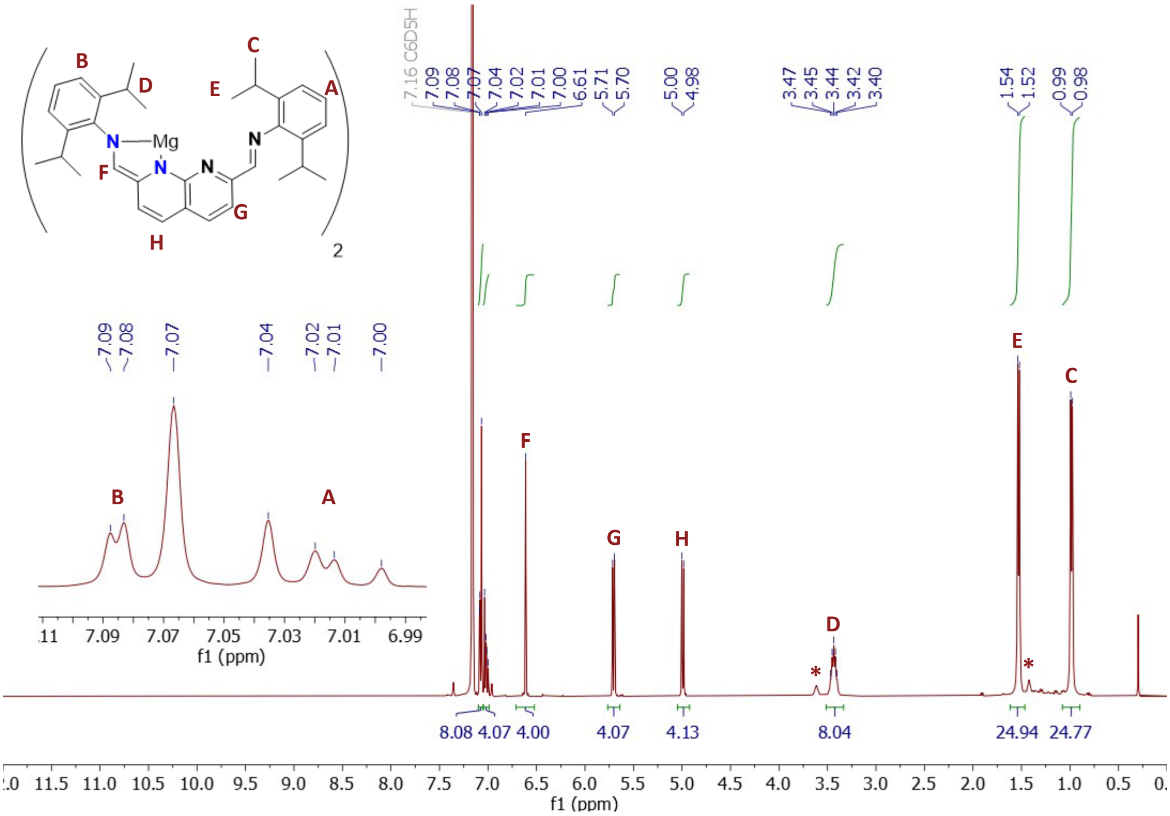

Figure S1: ^{1}H-NMR spectrum of **1** in C_6D_6 at 298 K. Resonances marked with an * are attributed to residual THF. The inset shows the zoomed-in region of the aromatic dipp resonances.

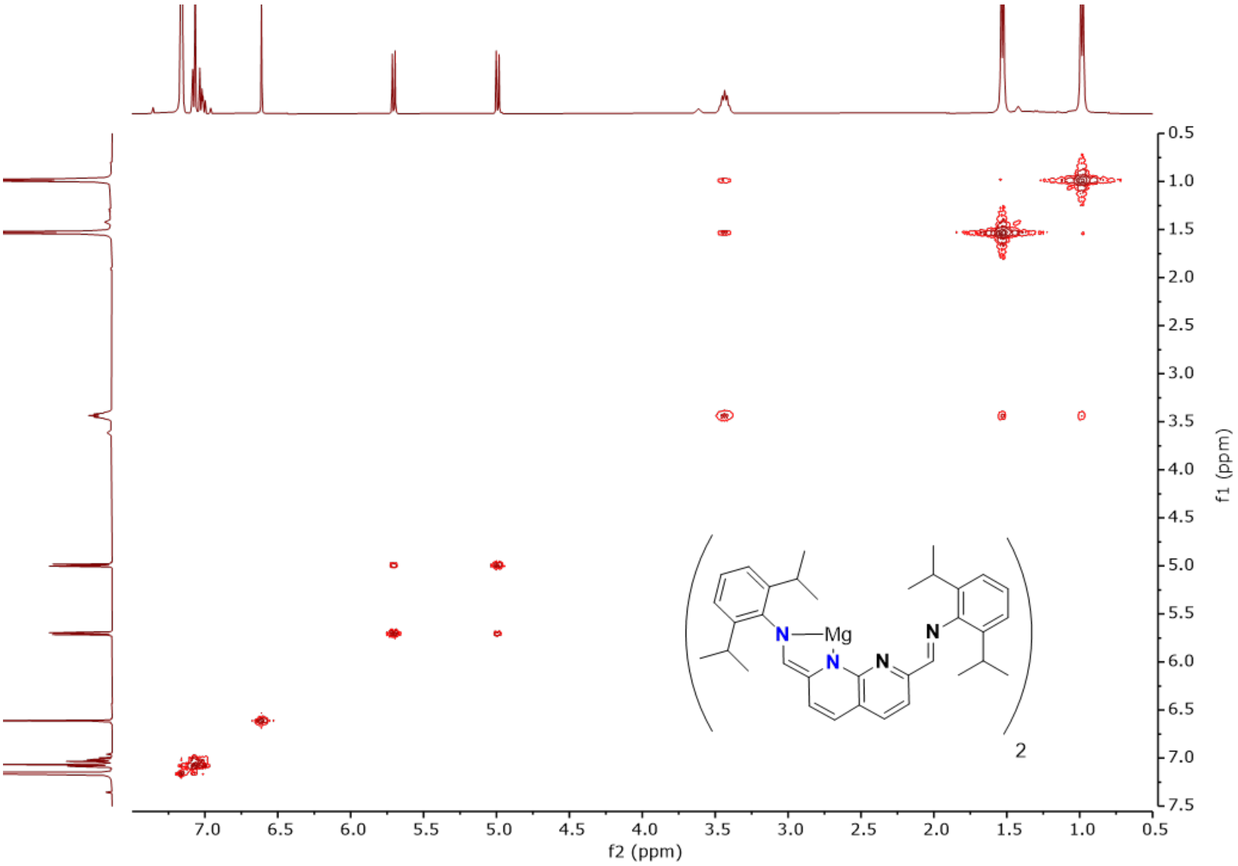

Figure S2: ^{1}H COSY-NMR spectrum of **1** in C_6D_6 at 298 K.

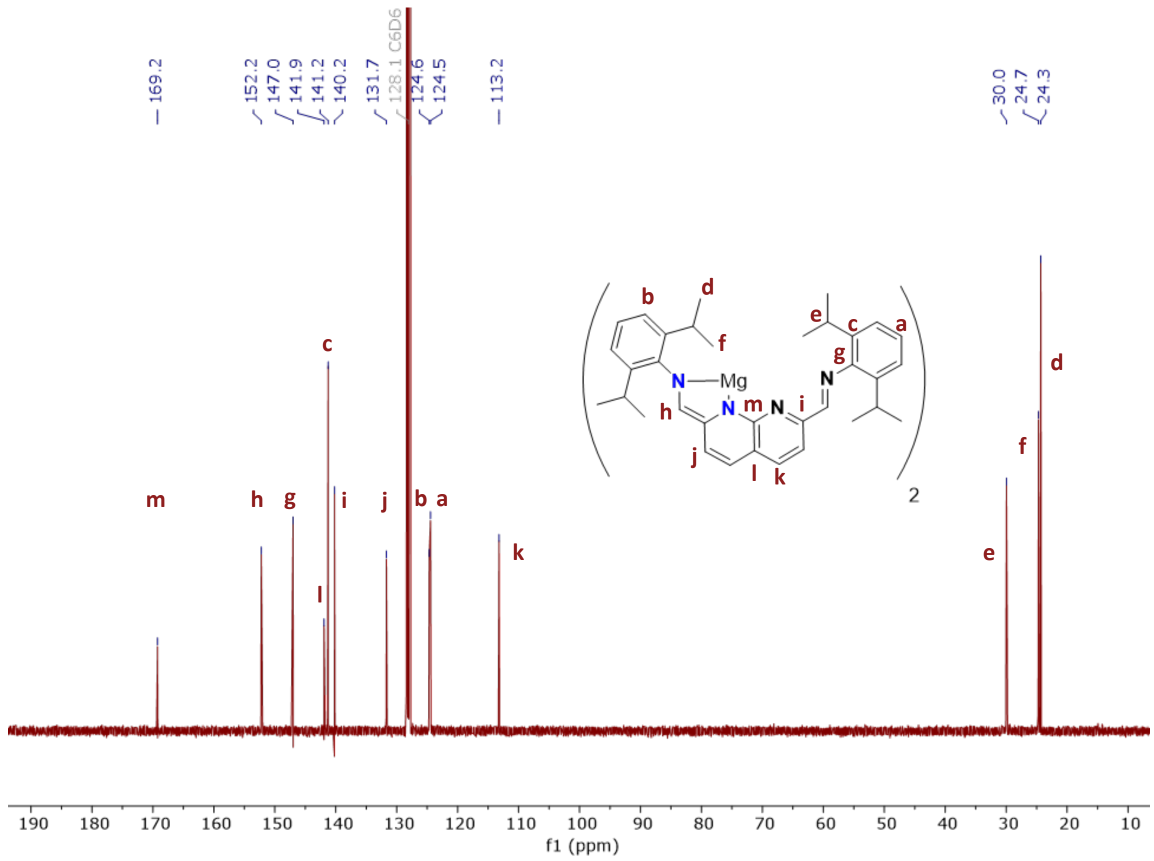

Figure S3: $^{13}C\{^{1}H\}$-NMR spectrum of **1** in C_6D_6 at 298 K.

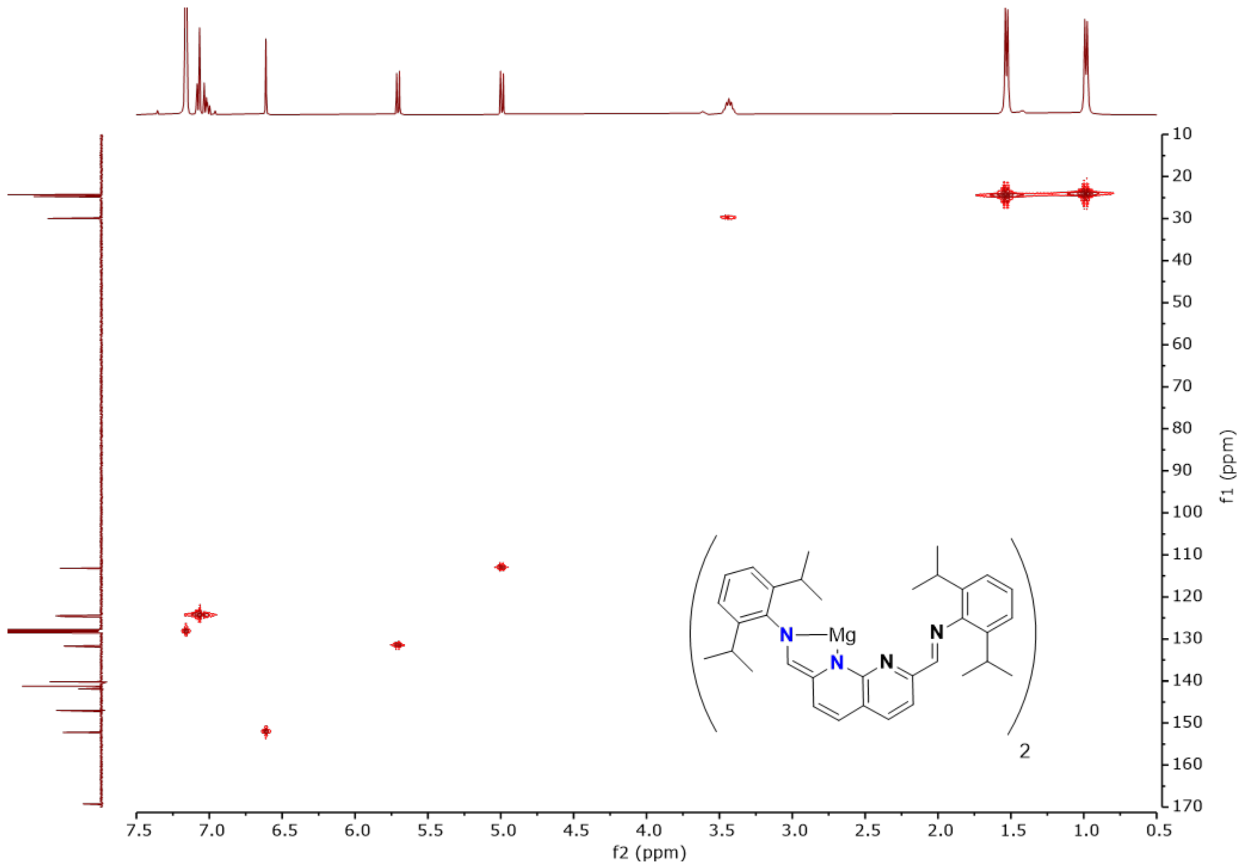

Figure S4: ^{1}H-^{13}C HMQC-NMR spectrum of **1** in C_6D_6 at 298 K.

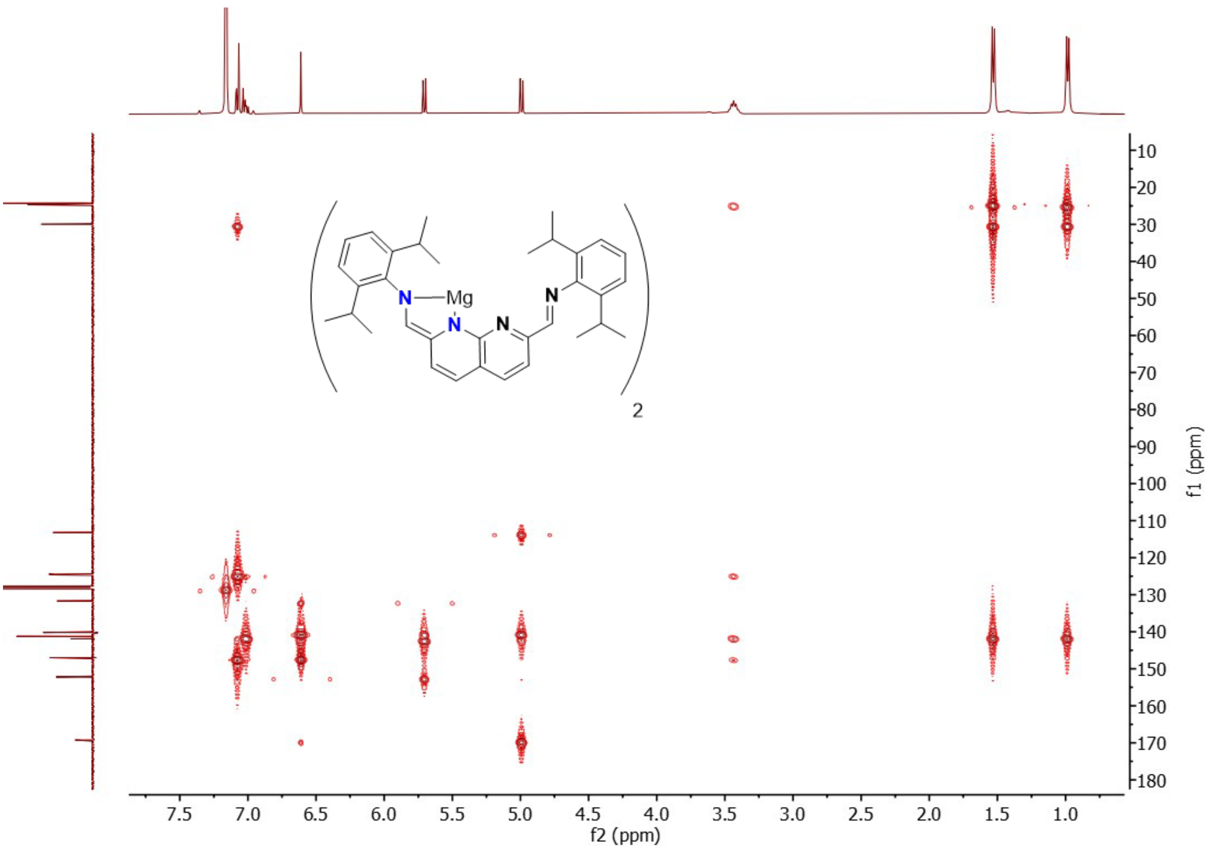

Figure S5: ^{1}H-^{13}C HMBC-NMR spectrum of **1** in C_6D_6 at 298 K.

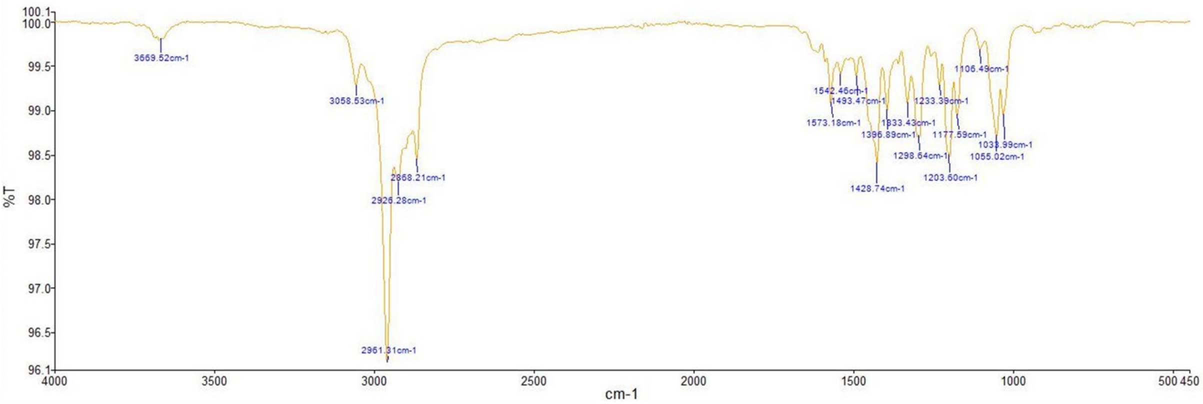

Figure S6: ATR-IR spectrum of **1** measured as a film under N_2 flow at 298 K.

1.3 Synthesis of ^{dipp}NBA*Ge (2):

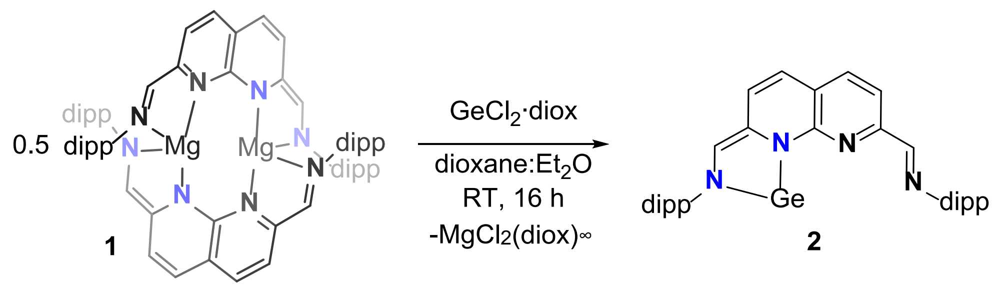

A 3:1 (v/v) 1,4-dioxane:Et_2O solution (1.5 mL) of $GeCl_2$·dioxane (17.5 mg, 75.6 µmol, 2.0 equiv) was added to a stirring solution of **1** (40.0 mg, 37.8 µmol, 1.0 equiv) in 3:1 (v/v) 1,4-dioxane:Et_2O (1.5 mL) and left to stir at ambient temperature for 16 h. The resulting mixture was subjected to pipette filtration over a short pad (<0.5 cm) of pre-wetted Celite® and dried under vacuum to a dark red solid. This solid was extracted with benzene (4 x 1 mL) and the extracts were subjected to pipette filtration over a short pad (<0.5 cm) of pre-wetted Celite® and dried under vacuum to a dark red solid. The solid was stripped with pentane (1 mL, to remove residual benzene), after which the volatiles were removed under vacuum, yielding **2** as a dark red solid (42.5 mg, 73.6 µmol, 97% yield).

^{1}H-NMR (400 MHz, C_6D_6, 298 K): δ 8.40 (s, 1H), 8.14 (d, $^{3}J_{H,H}$ = 7.7 Hz, 1H), *, 7.02 (d, $^{3}J_{H,H}$ = 7.7 Hz, 1H), 6.79 (s, 1H), 6.55 (d, $^{3}J_{H,H}$ = 9.1 Hz, 1H), 6.06 (d, $^{3}J_{H,H}$ = 9.1 Hz, 1H), 3.20 (sept, $^{3}J_{H,H}$ = 6.8 Hz, 2H), 2.88 (apparent sept, $^{3}J_{H,H}$ = 6.8 Hz, 2H), 1.18 (d, $^{3}J_{H,H}$ = 6.8 Hz, 12H), 1.12 (d, $^{3}J_{H,H}$ = 7.1, Hz, 6H), 1.11 (d $^{3}J_{H,H}$ = 7.3, 6H).

^{13}C{^{1}H}-NMR (100 MHz, C_6D_6, 298 K): δ 163.3, 155.2, 151.2, 149.7, 145.0, 141.3, 137.5, 135.1, 133.7, 125.4, 124.9, 124.4, 123.6, 123.5, 120.4, 119.1, 117.4, 28.5, 28.3, 25.9, 24.6, 23.6.

*Aromatic dipp resonances are overlapped by the C_6D_5H resonance.

ATR-IR (cm^{-1}): 3062 (m), 2960 (s), 2926 (m), 2868 (m), 1637 (m), 1461 (w), 1394 (w), 1342 (m), 1139 (m), 1058 (w).

Elemental Analysis: The reactive nature of this compound precluded obtaining satisfactory elemental analysis. To ensure the complete removal of $MgCl_2$, a small sample of **2** was digested by $HNO_{3(aq)}$ and treated with $AgNO_3$, upon which no precipitate was observed, suggestive of the absence of Cl^- in the sample of **2**.

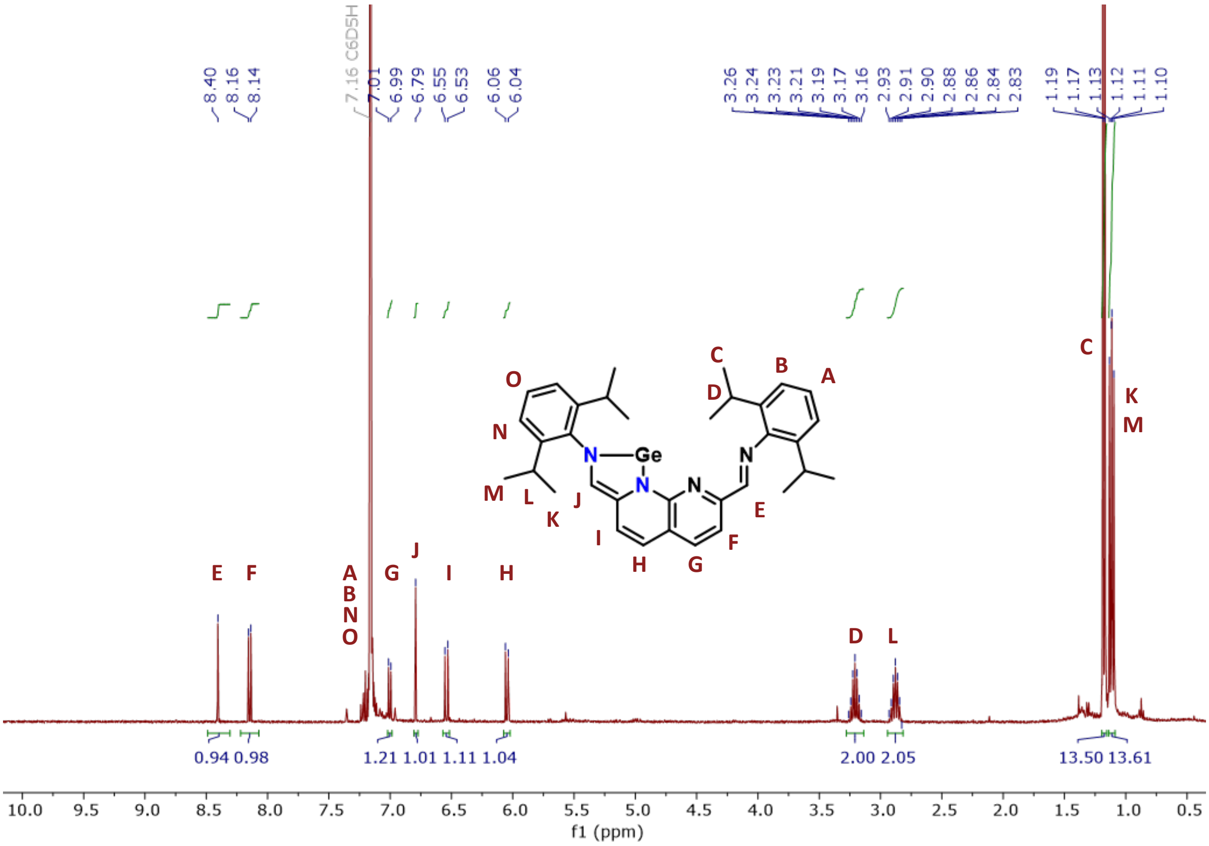

Figure S7: ^{1}H-NMR spectrum of **2** in C_6D_6 at 298 K.

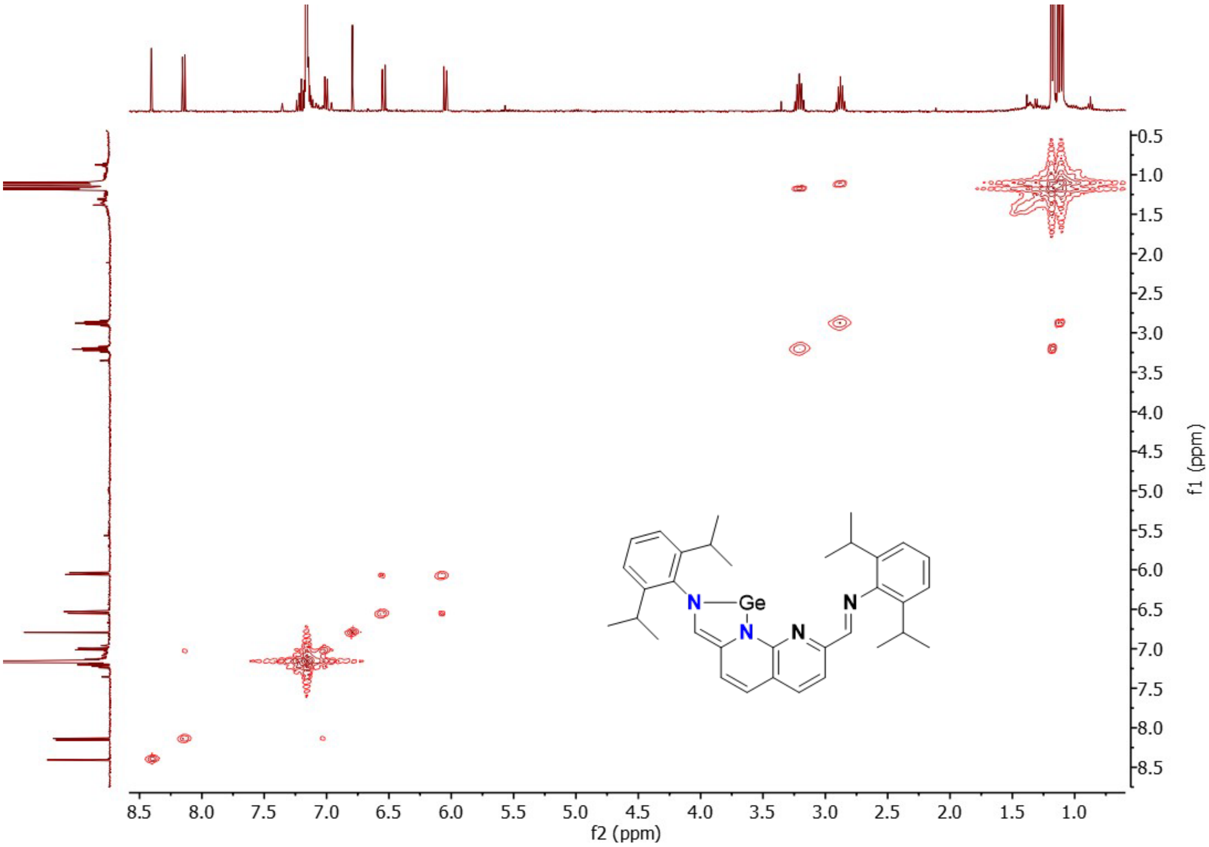

Figure S8: ^{1}H COSY-NMR spectrum of **2** in C_6D_6 at 298 K.

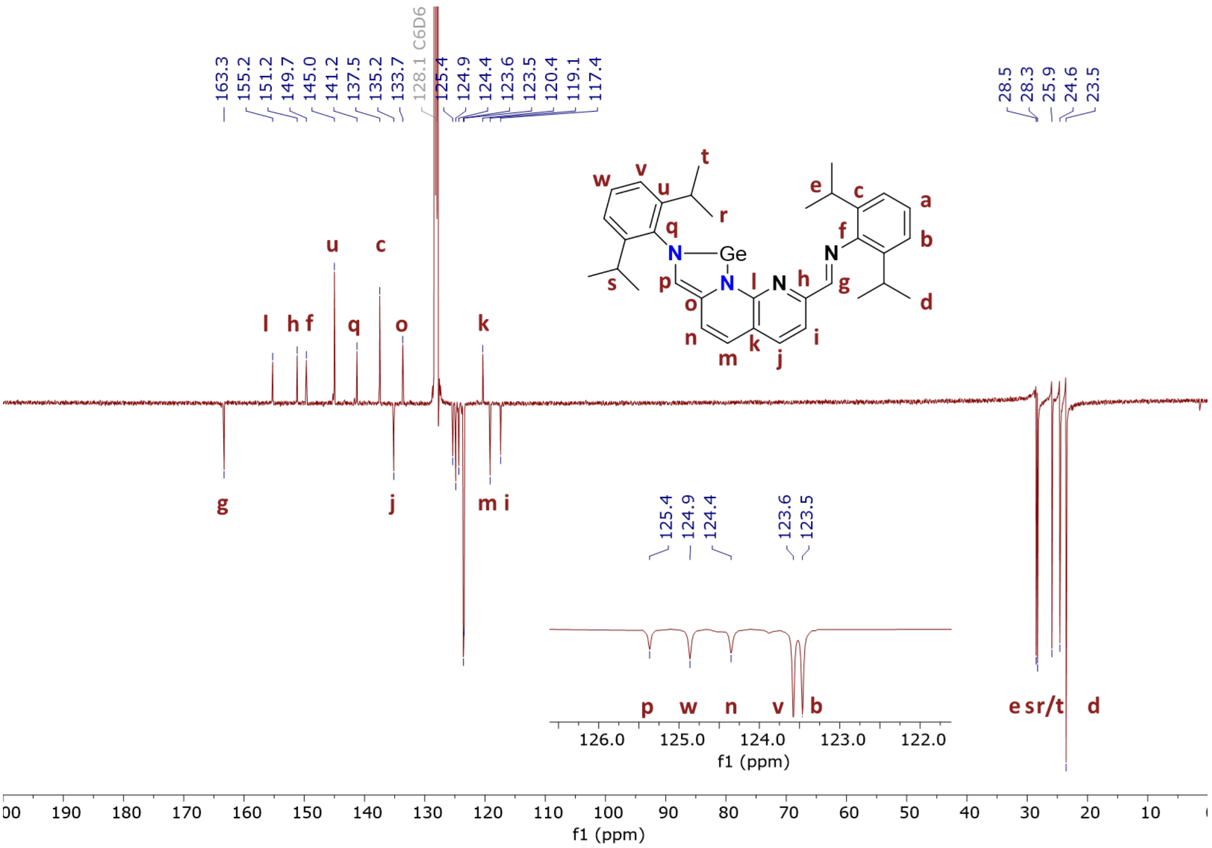

Figure S9: $^{13}C\{^{1}H\}$-NMR (APT) spectrum of **2** in C_6D_6 at 298 K. The inset shows the zoomed-in region between 122-126 ppm.

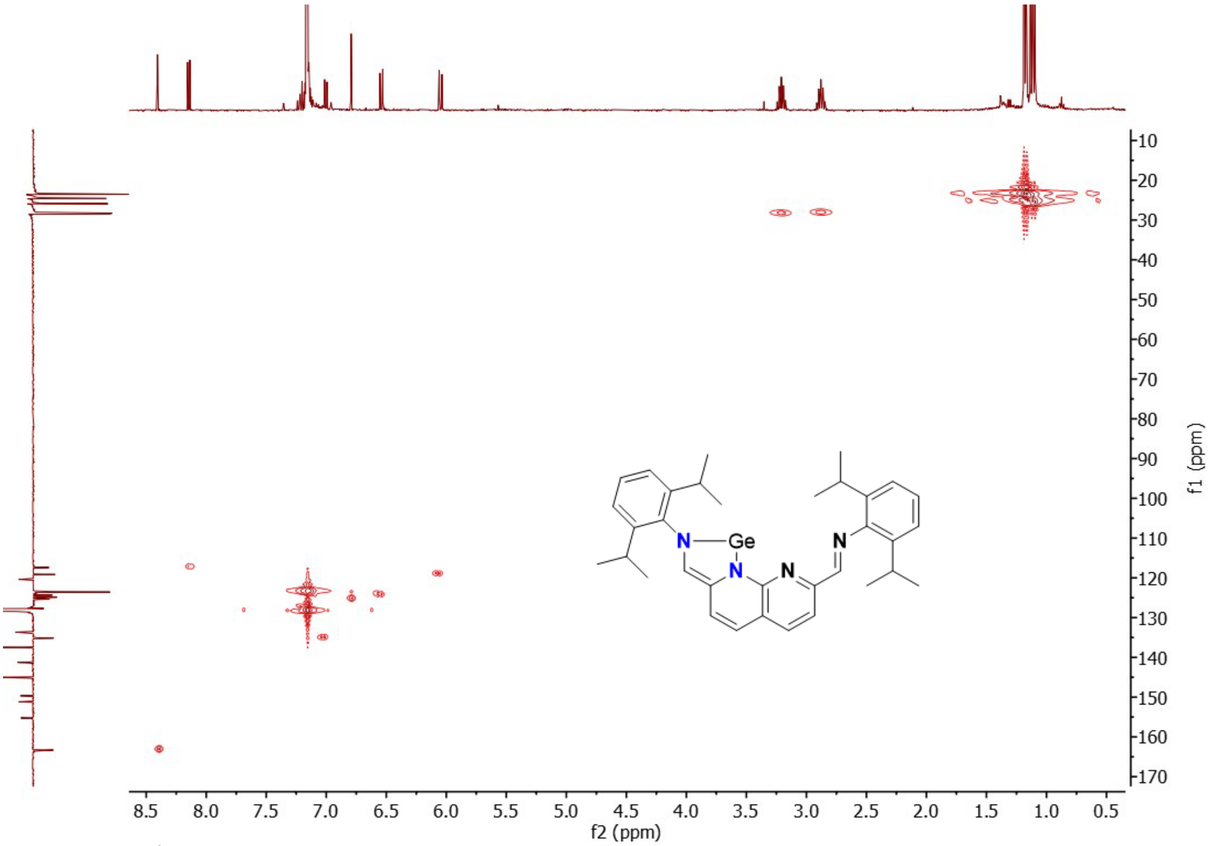

Figure S10: ^{1}H-^{13}C HMQC-NMR spectrum of **2** in C_6D_6 at 298 K.

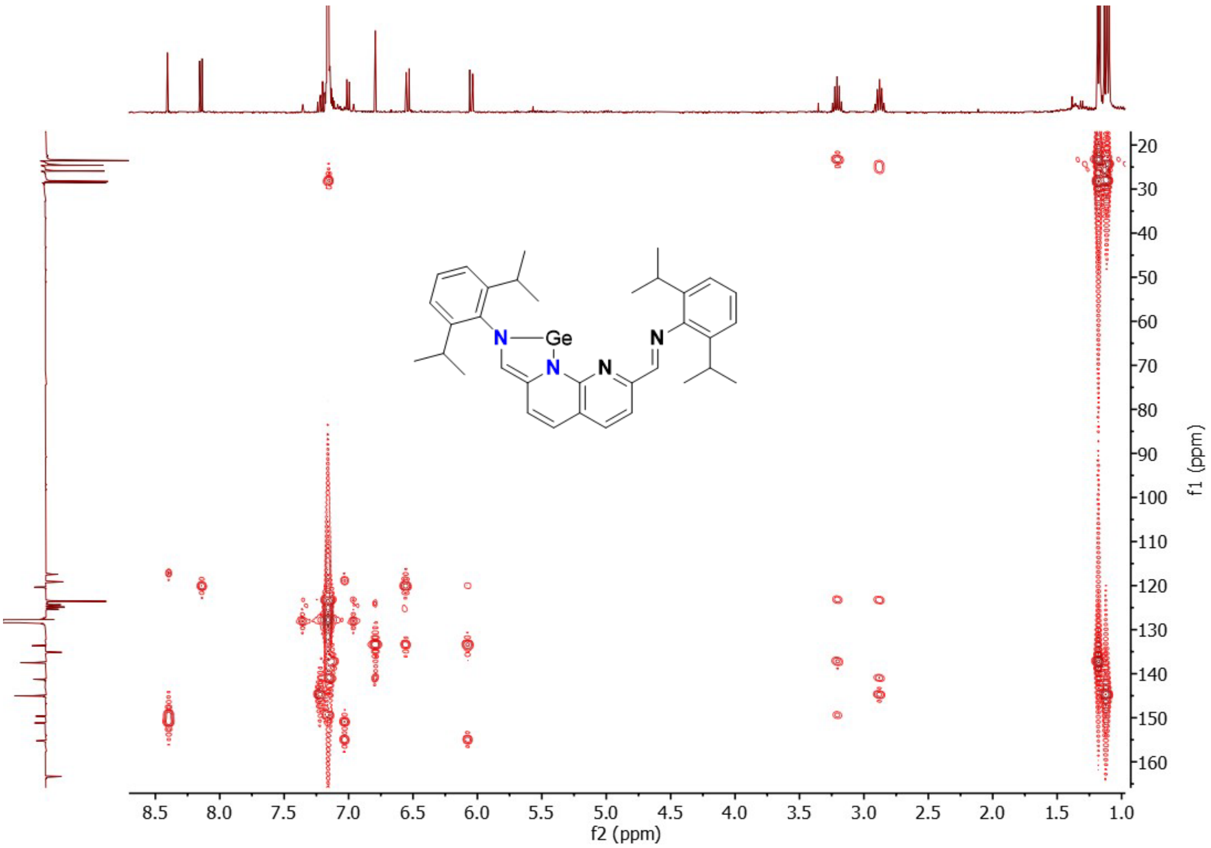

Figure S11: ^{1}H-^{13}C HMBC-NMR spectrum of **2** in C_6D_6 at 298 K.

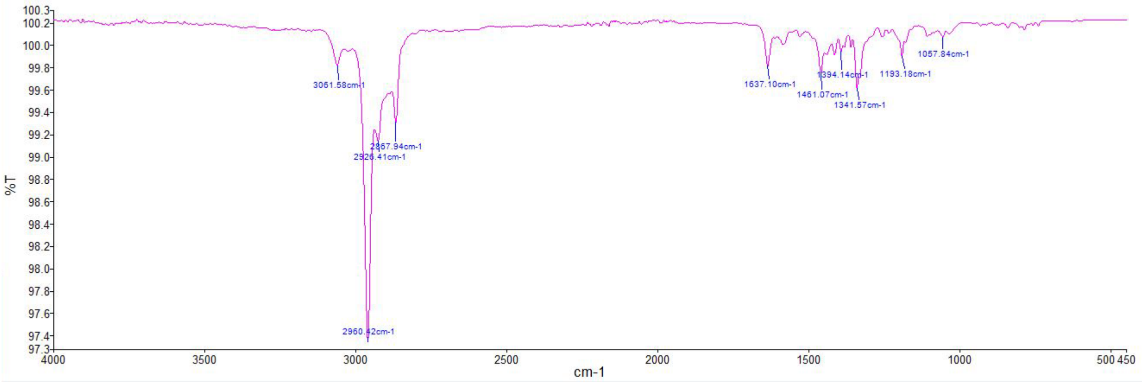

Figure S12: ATR-IR spectrum of **2** measured as a film under N_2 flow at 298 K.

1.4 ccDOSY-NMR Analysis of ^{dipp}**NBA***Ge:

We recorded the ccDOSY spectra of equimolar solutions of **2** with monomeric ^{dipp}**NBA**, and **2** with dimer **1**. Analysis of the ccDOSY spectrum of the mixture of **2** with ^{dipp}**NBA** (see **Figures** S**13-14**) revealed comparable diffusion coefficients amongst the two components, indicative of **2** being monomeric. Analysis of the ccDOSY spectrum of the mixture of **2** with **1** (see **Figures S15-16**) further corroborated this hypothesis, with **1** having a significantly smaller diffusion coefficient (and hence larger radius) than **2**.

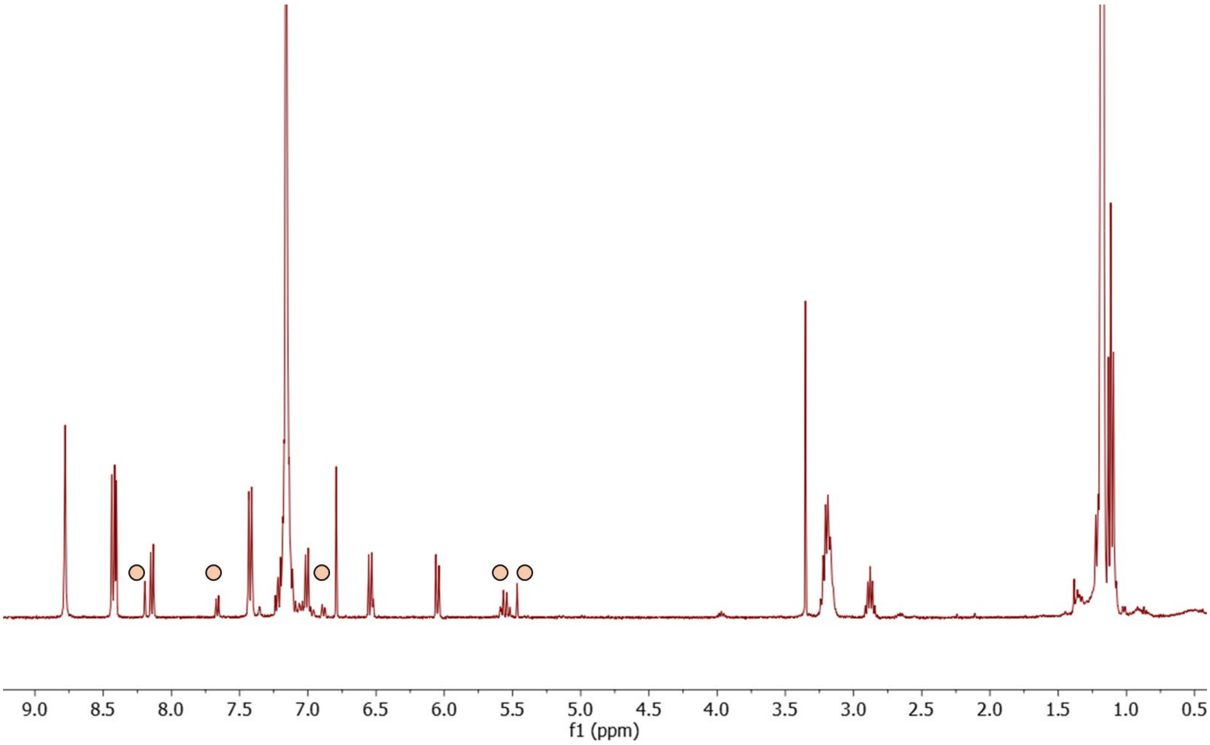

Figure S13: ^{1}H-NMR spectrum of an equimolar mixture of **2** and ^{dipp}**NBA** in C_6D_6, recorded at 298 K. Resonances indicated with a cream-coloured circle are attributed to an unknown species that forms upon mixing of the components. The intensity of these resonances does not increase over time.

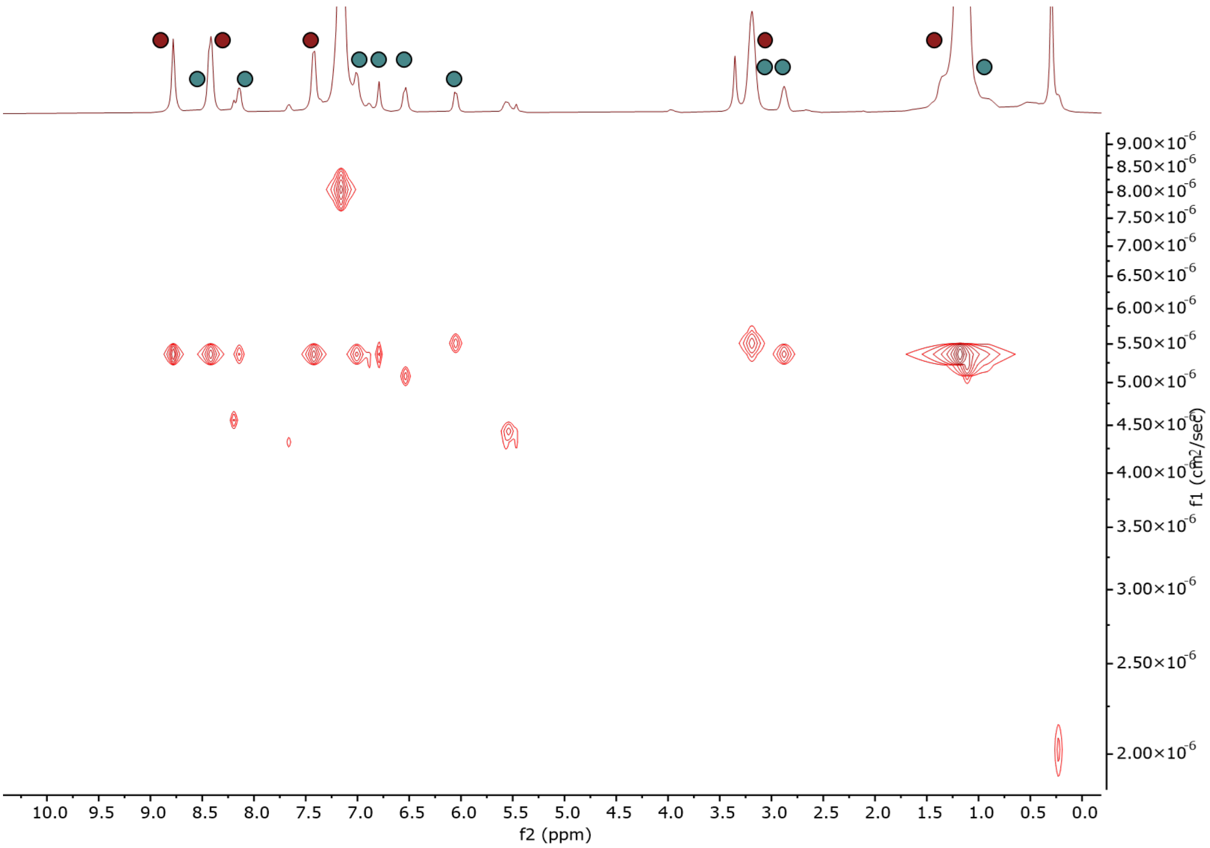

Figure S14: ccDOSY-NMR spectrum of an equimolar mixture of **2** (teal) and **^{dipp}NBA** (maroon) in C_6D_6, recorded at 298 K. Measured in 32 increments from 5 to 80 Gcm^{-1}. Relaxation delay = 13 s, apodisation = 10.0 Hz, gradient length = 1 ms, diffusion delay = 140 ms.

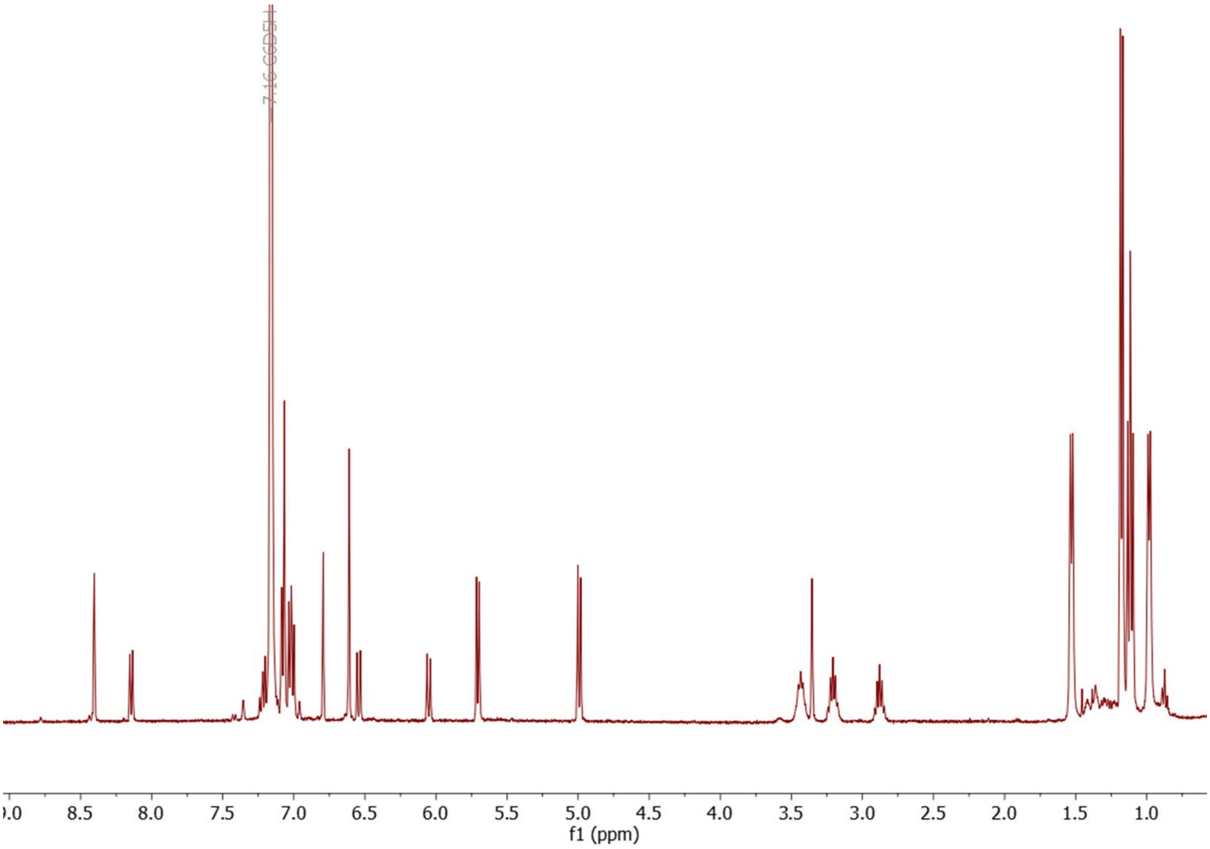

Figure S15: ^{1}H-NMR spectrum of an equimolar mixture of **2** and **1** in C_6D_6, recorded at 298 K.

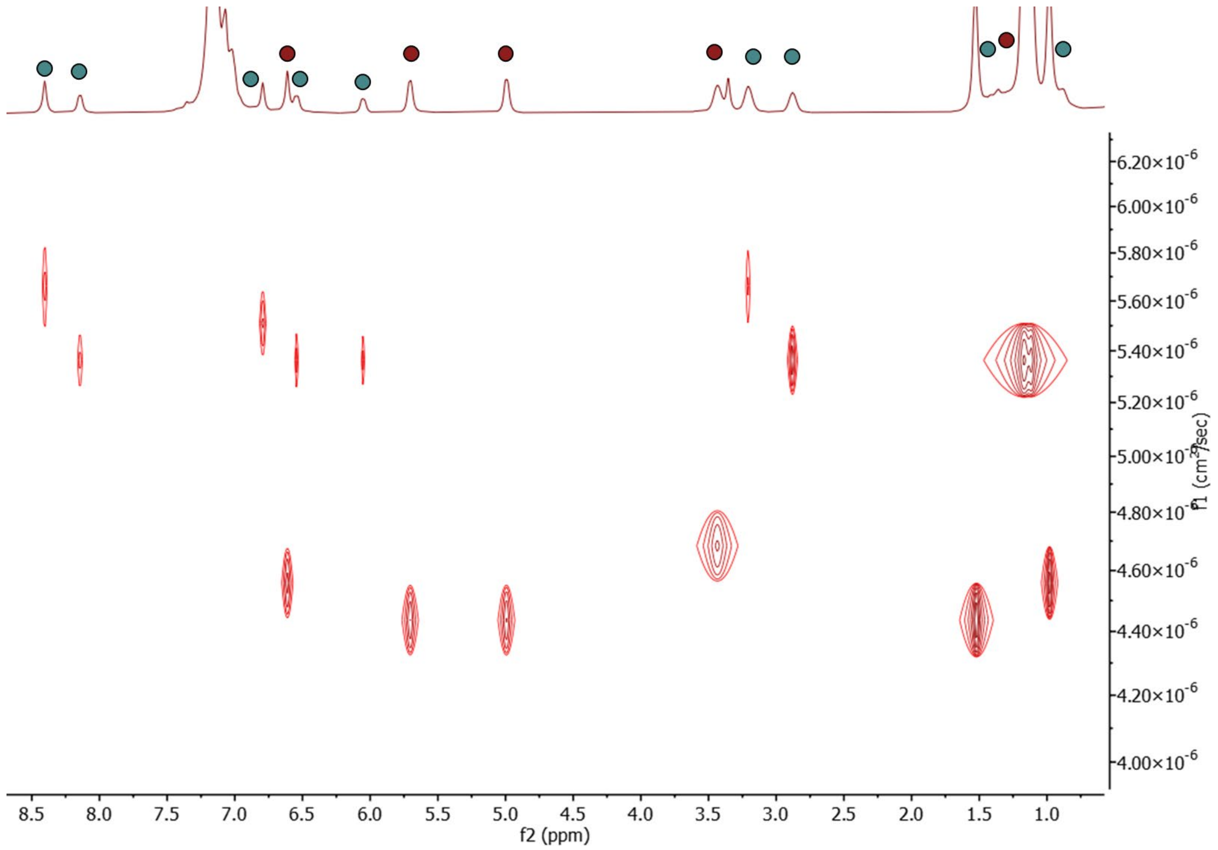

Figure S16: ccDOSY-NMR spectrum of an equimolar mixture of **2** (teal) and **1** (maroon) in C_6D_6, recorded at 298 K. Measured in 32 increments from 5 to 80 Gcm^{-1}. Relaxation delay = 13 s, apodisation = 10.0 Hz, gradient length = 1 ms, diffusion delay = 145 ms.

1.5 Synthesis of ^{dipp}**NBA***GeZnCl$_2$ (**3**):

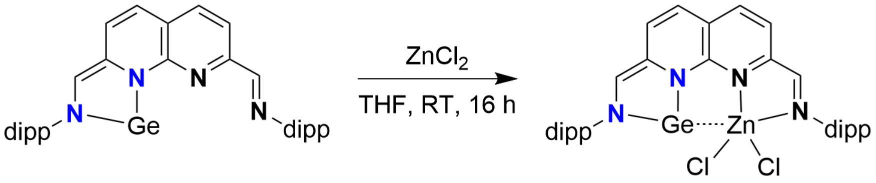

2 **3**

A solution of $ZnCl_2$ (5.1 mg, 37.6 µmol, 1.0 equiv) in THF (1.5 mL) was added to a stirring solution of **2** (21.7 mg, 8.7 µmol, 1.0 equiv) in THF (2 mL) and left to stir overnight at ambient temperature. The red/purple mixture was then subjected to pipette filtration over a short pad (<0.5 cm) of pre-wetted Celite® and dried under vacuum to a blue solid*. The solid was washed with pentane (3 x 1.5 mL) and dried under vacuum, yielding **3** as a blue solid (18.2 mg, 25.5 µmol, 68%).

Single crystals suitable for analysis by X-ray diffraction were grown by slow vapour diffusion of pentane into a saturated 1,4-dioxane solution of **3**.

*The blue colour persists in dioxane and aromatic solvents. Redissolving in THF reforms the red/purple colour. When swirling a red/purple solution of **3** in a vial the blue colour is observed on the drying sides of the vial, consistent with weak binding of THF.

^{1}H-NMR (400 MHz, THF-*d*$_8$, 298 K): δ 8.24 (s, 1H), 7.80 (broad, 1H), 7.64 (d, $^3J_{H,H}$ = 7.5 Hz, 1H), 7.27-7.21 (m, 3H), 7.17-7.11 (m, 3H), 7.01 (d, $^3J_{H,H}$ = 9.1 Hz, 1H), 6.98 (s, 1H), 6.53 (d, $^3J_{H,H}$ = 9.1 Hz, 1H), 3.11 (broad, 2H), 2.89 (sept, $^3J_{H,H}$ = 6.8 Hz, 2H), 1.20-1.15 (m, 24H).

^{13}C{^{1}H}-NMR (100 MHz, THF-*d*$_8$, 298 K): δ 164.9, 157.3 (br), 145.9, 141.3, 135.4, 134.1, 127.3, 126.3, 124.1, 119.2, 29.0. The fluxional nature of **3** prevents obtaining a satisfactory APT spectrum and as such we recorded a normal ^{13}C{^{1}H}-NMR spectrum instead.

ATR-IR (cm^{-1}): 2961 (s), 2924 (s), 2868 (m), 1613 (w), 1586 (w), 1460 (s), 1400 (m), 1384 (m), 1364 (w), 1330 (s), 1258 (m), 1240 (m), 1196 (m), 1176 (m), 1099 (w), 1069 (m), 1057 (m), 867 (w), 802 (m), 770 (w).

Elemental Analysis: The reactive nature of this compound precluded obtaining satisfactory elemental analysis.

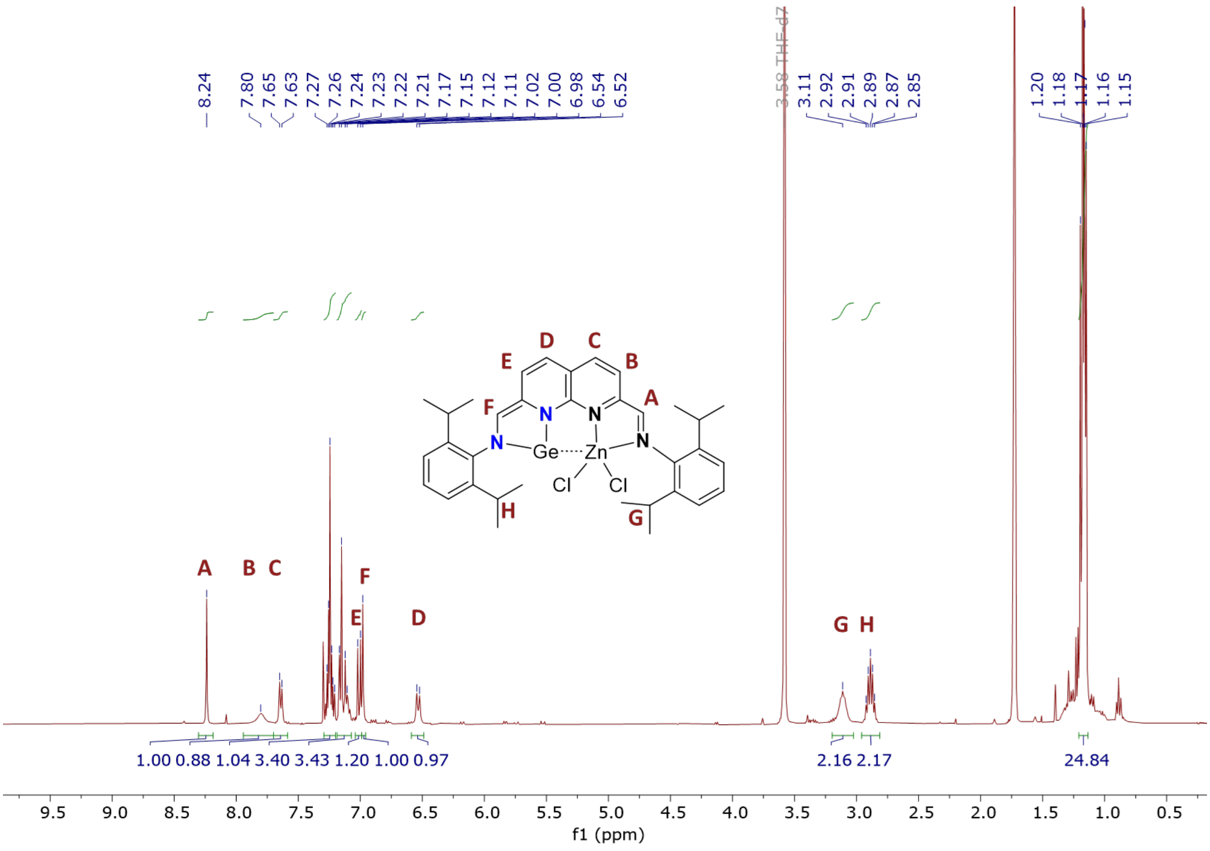

Figure S17: ^{1}H-NMR spectrum of **3** in THF-d_8 at 298 K.

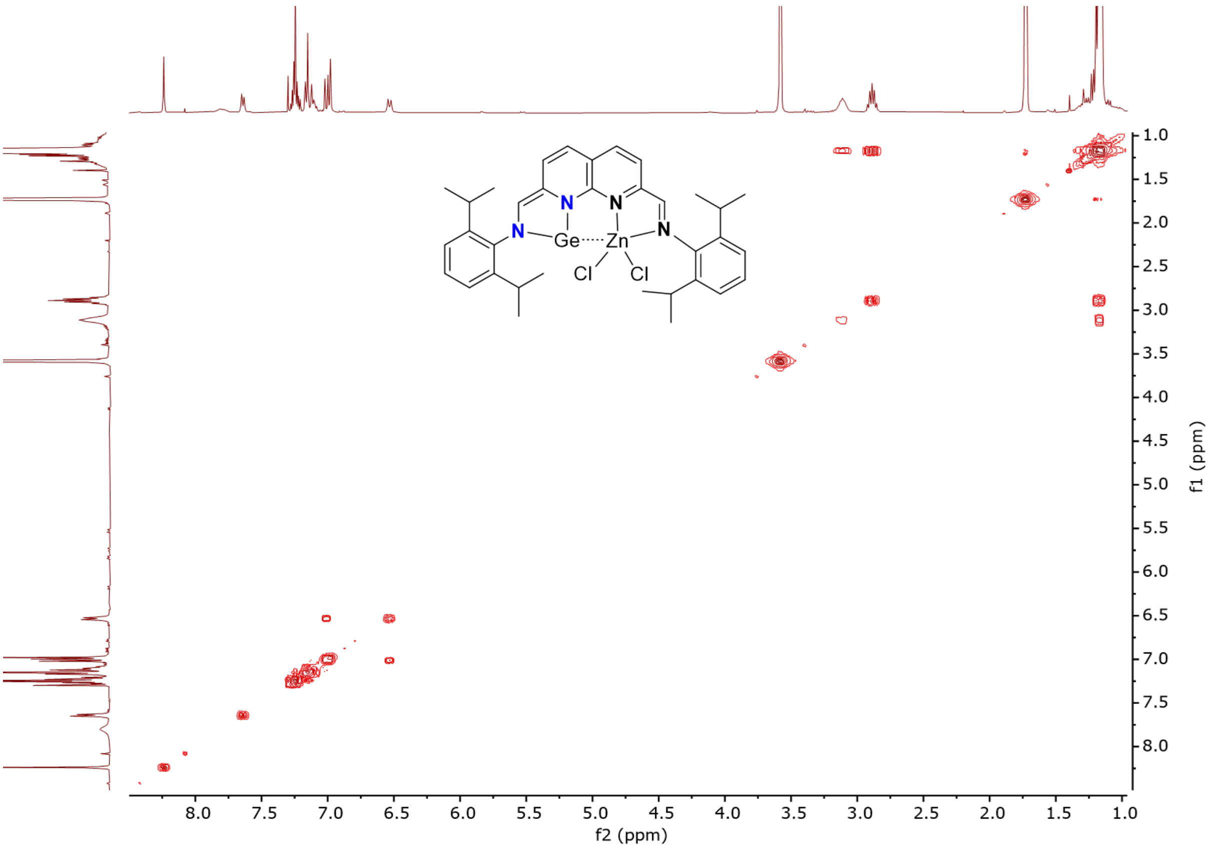

Figure S18: ^{1}H COSY-NMR spectrum of **3** in THF-d_8 at 298 K.

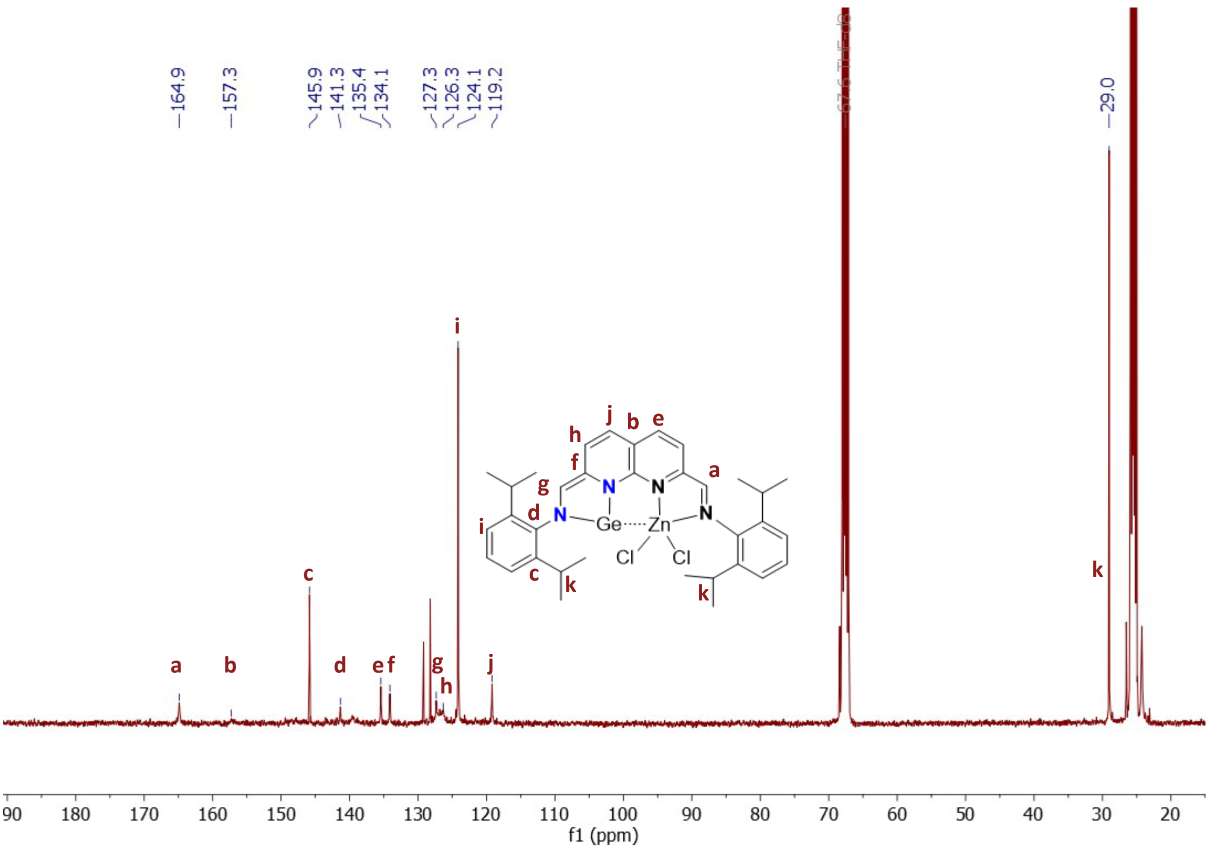

Figure S19: $^{13}C\{^{1}H\}$-NMR spectrum of **3** in THF-d_8 at 298 K. Only resonances that could be unambiguously assigned were labelled.

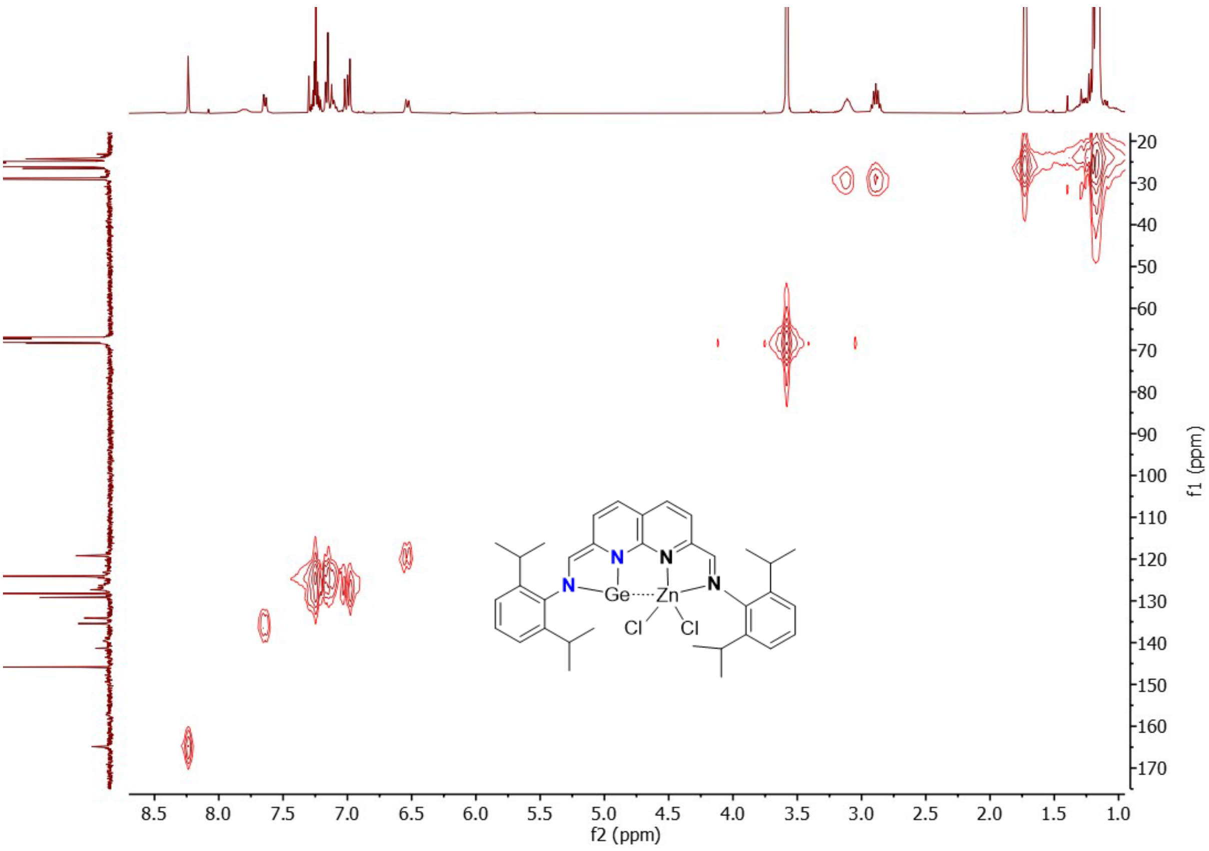

Figure S20: ^{1}H-^{13}C ASAP HMQC-NMR spectrum of **3** in THF-d_8 at 298 K.

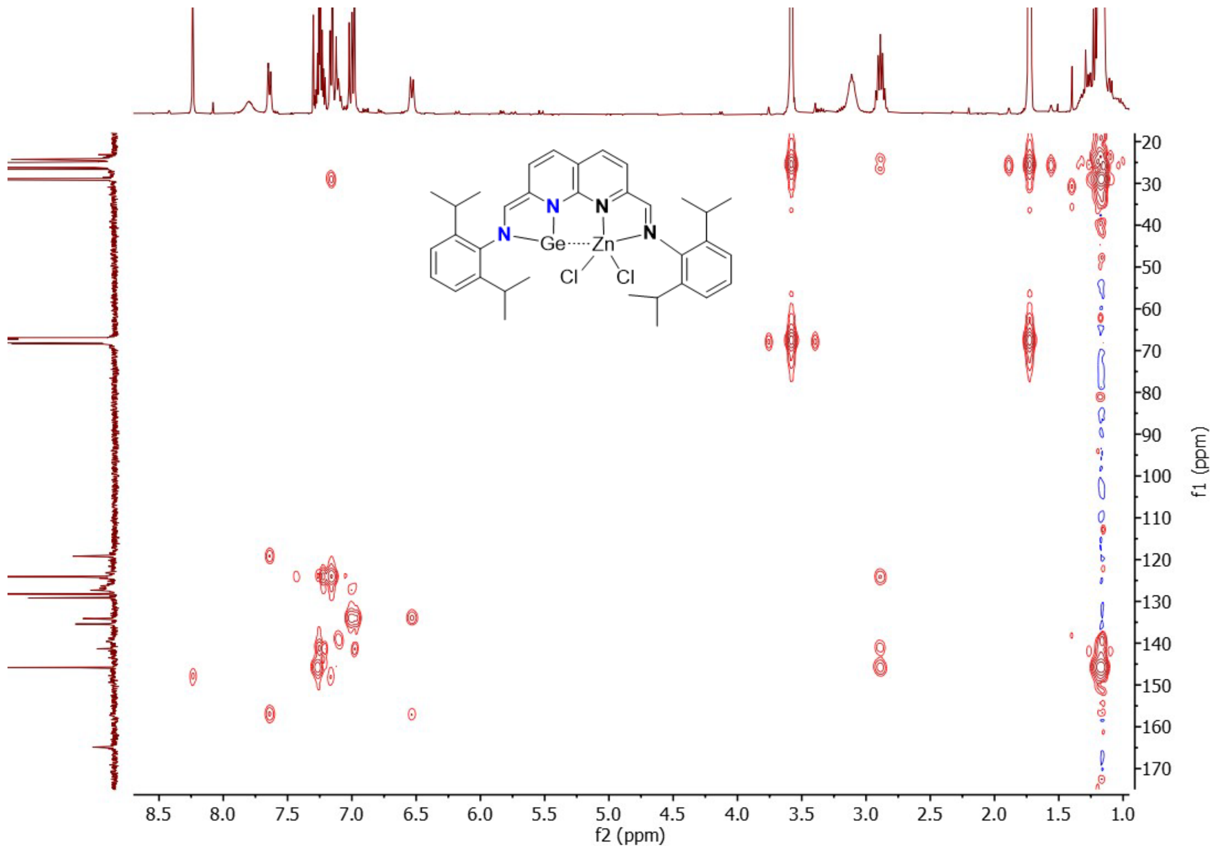

Figure S21: ^{1}H-^{13}C HMBC-NMR spectrum of **3** in THF-d_8 at 298 K.

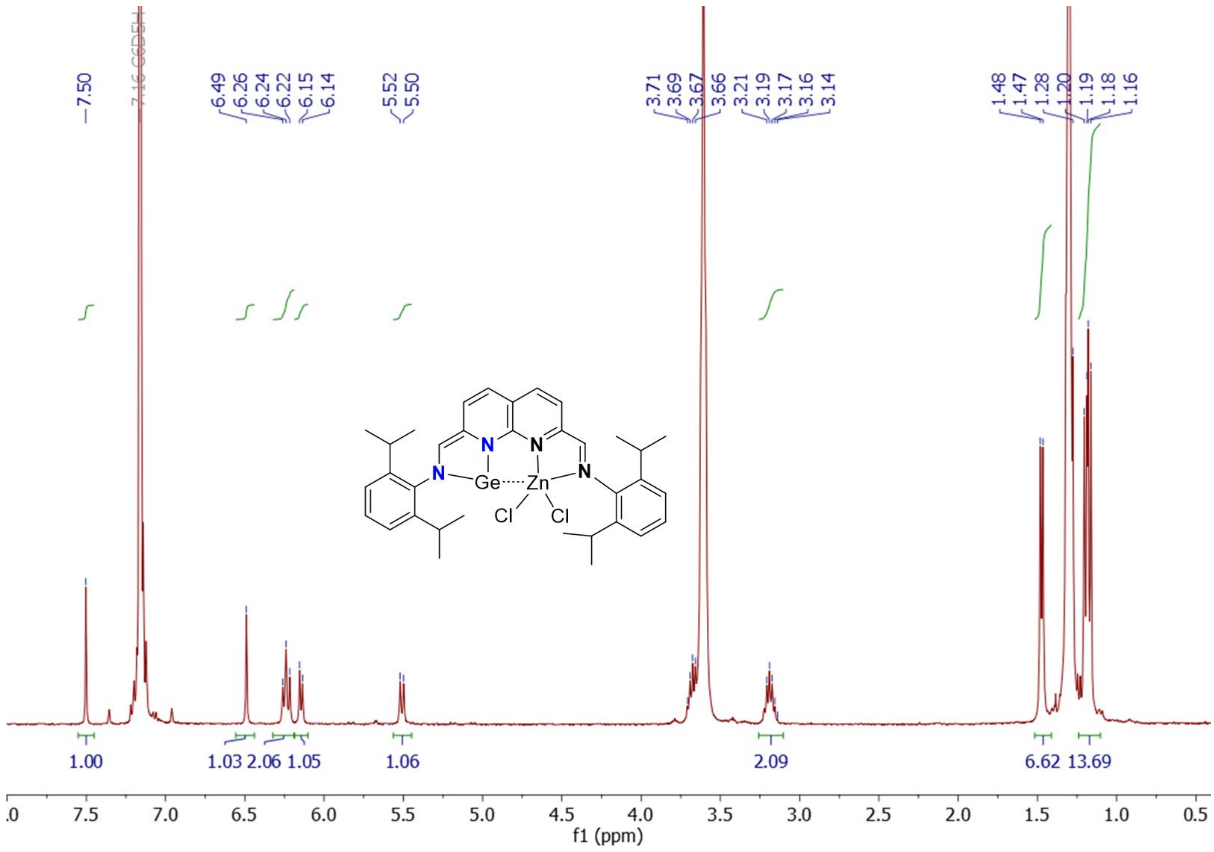

Figure S22: ^{1}H-NMR spectrum of **3** in C_6D_6 at 298 K, demonstrating sharper resonances despite the presence of residual THF.

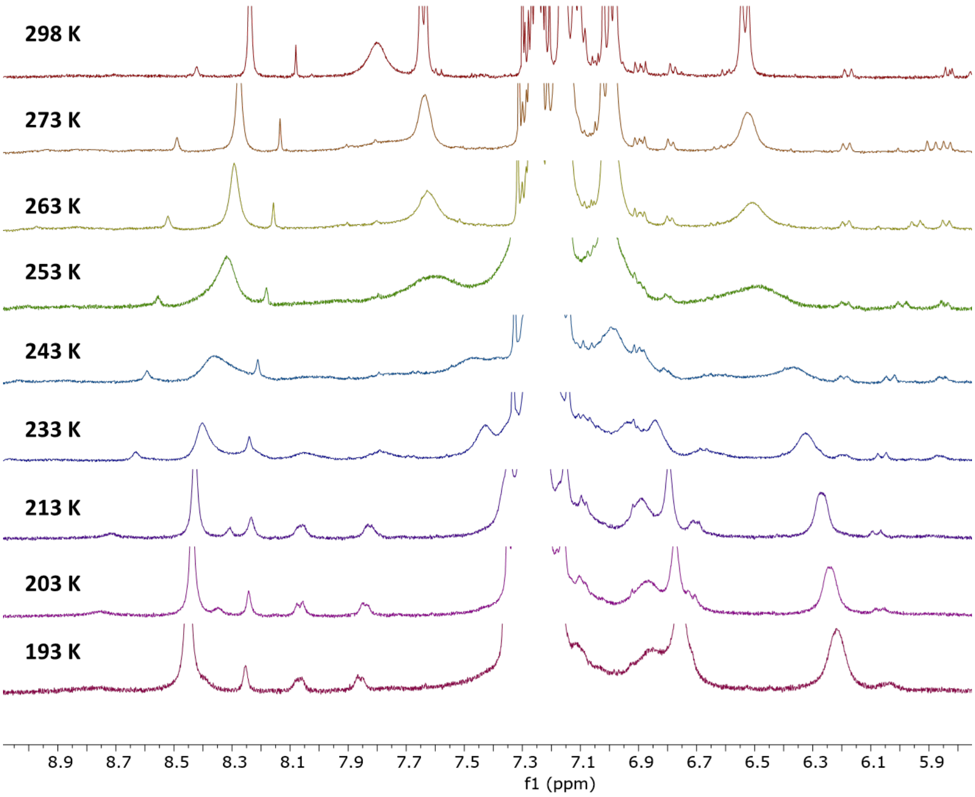

Figure S23: Stacked VT ^{1}H-NMR spectra of the aromatic region of **3** in THF-d_8, recorded at 298-193 K.

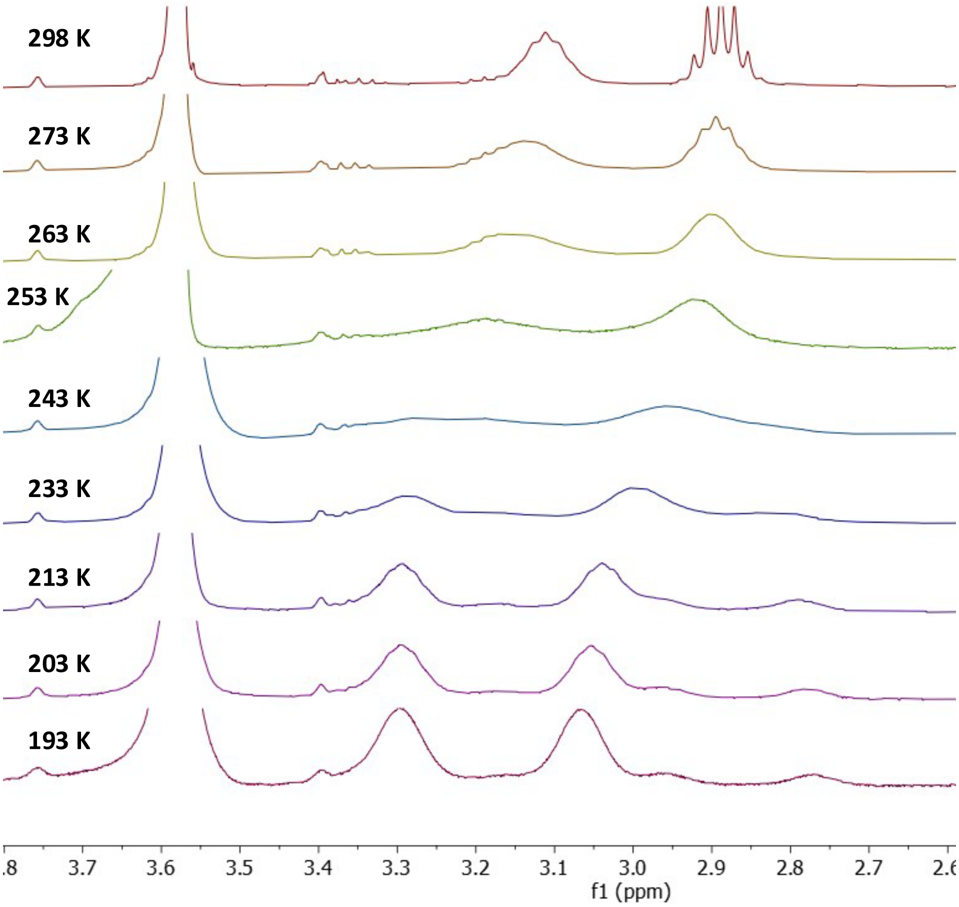

Figure S24: Stacked VT ^{1}H-NMR spectra of the dipp-methine region of **3** in THF-d_8, recorded at 298-193 K.

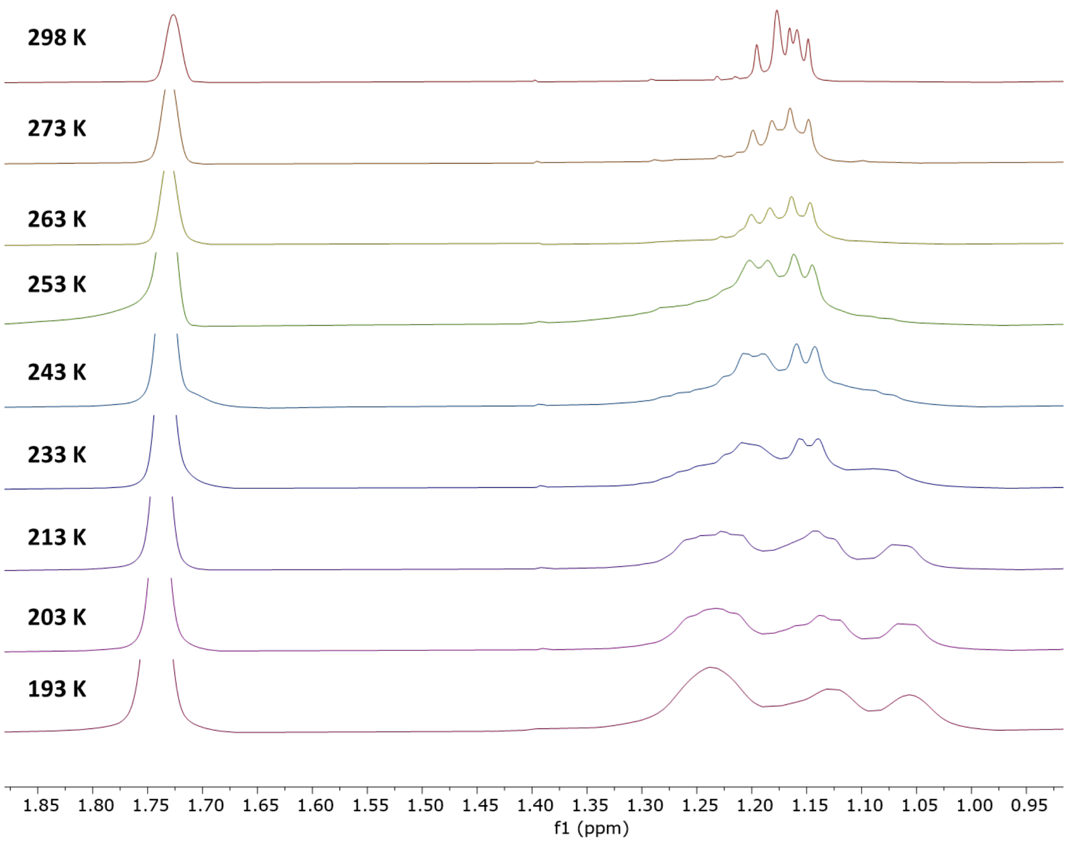

Figure S25 Stacked VT ^{1}H-NMR spectra of the dipp-methyl region of **3** in THF-d_8, recorded at 298-193 K.

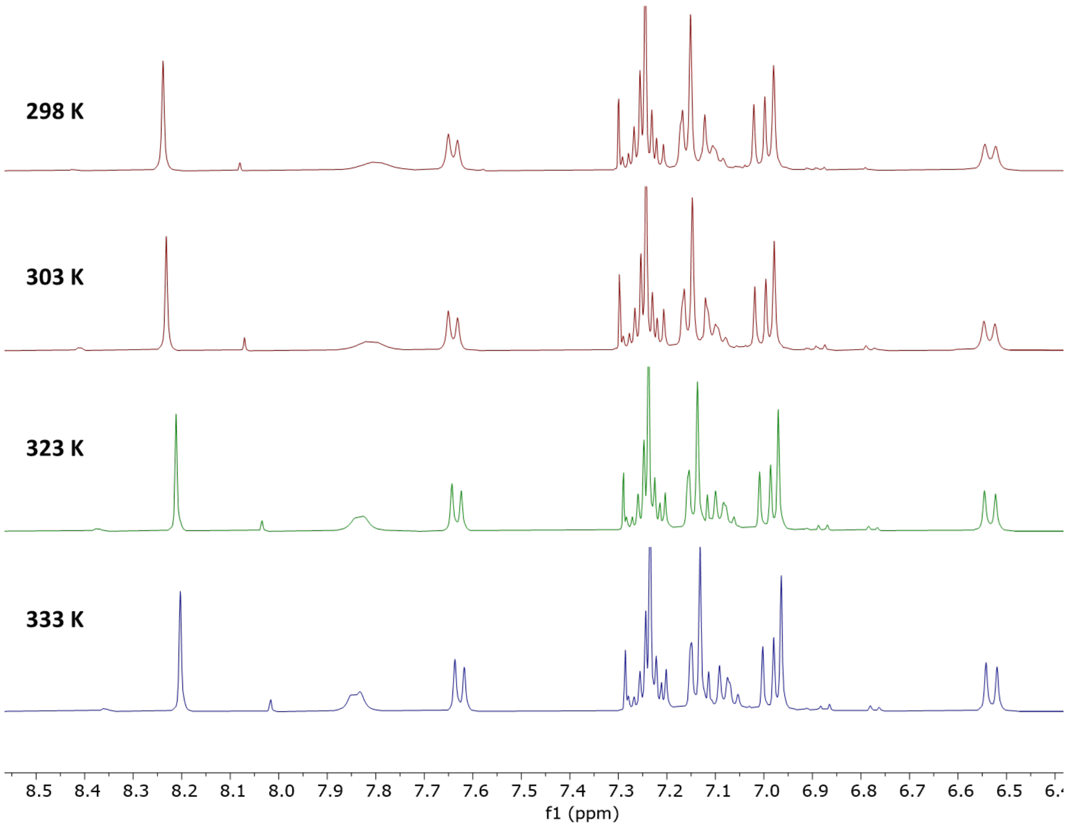

Figure S26: Stacked VT ^{1}H-NMR spectra of the dipp-methine region of **3** in THF-d_8, recorded at 298-333K.

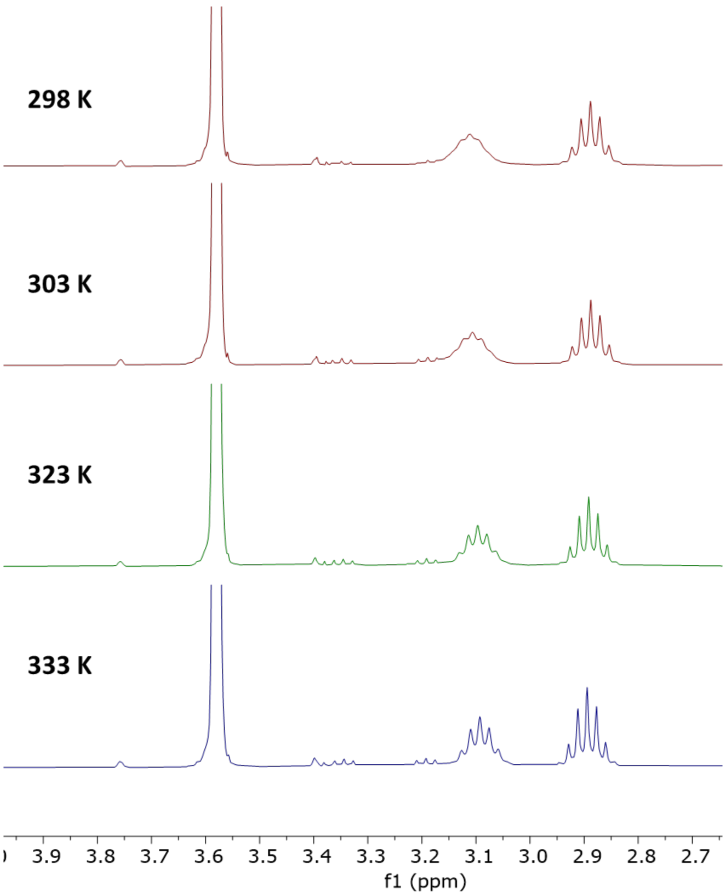

Figure S27: Stacked VT ^{1}H-NMR spectra of the dipp-methine region of **3** in THF-d_8, recorded at 298-333 K.

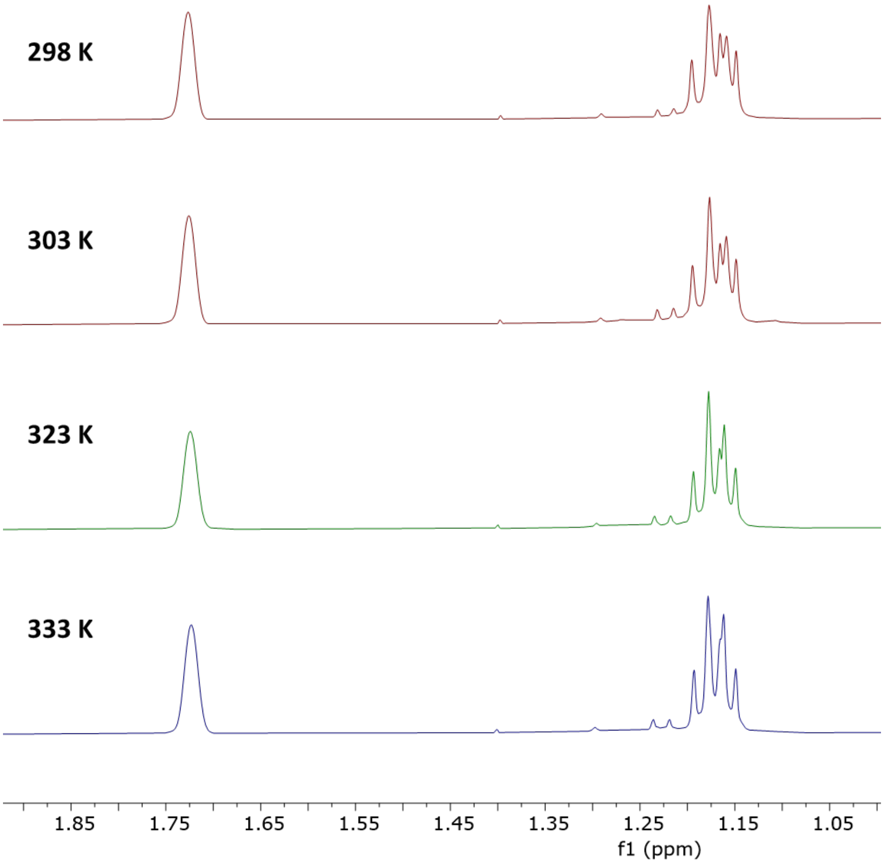

Figure S28: Stacked VT ^{1}H-NMR spectra of the dipp-methyl region of **3** in THF-d_8, recorded at 298-333 K.

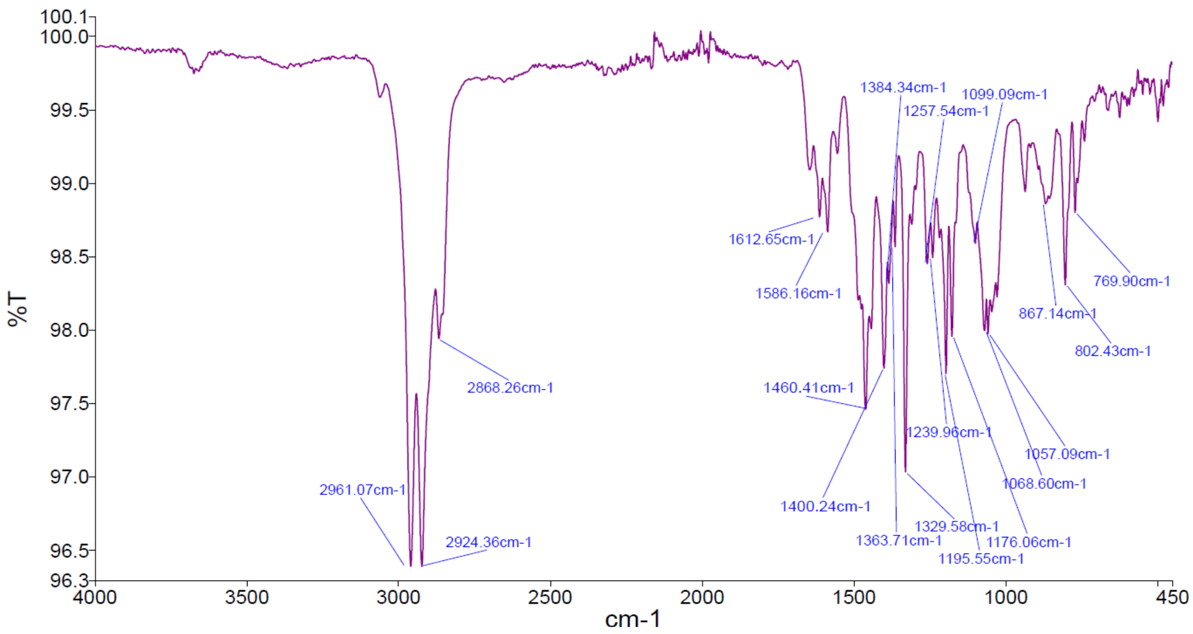

Figure S29: ATR-IR spectrum of **3** measured as a film under N_2 flow at 298 K.

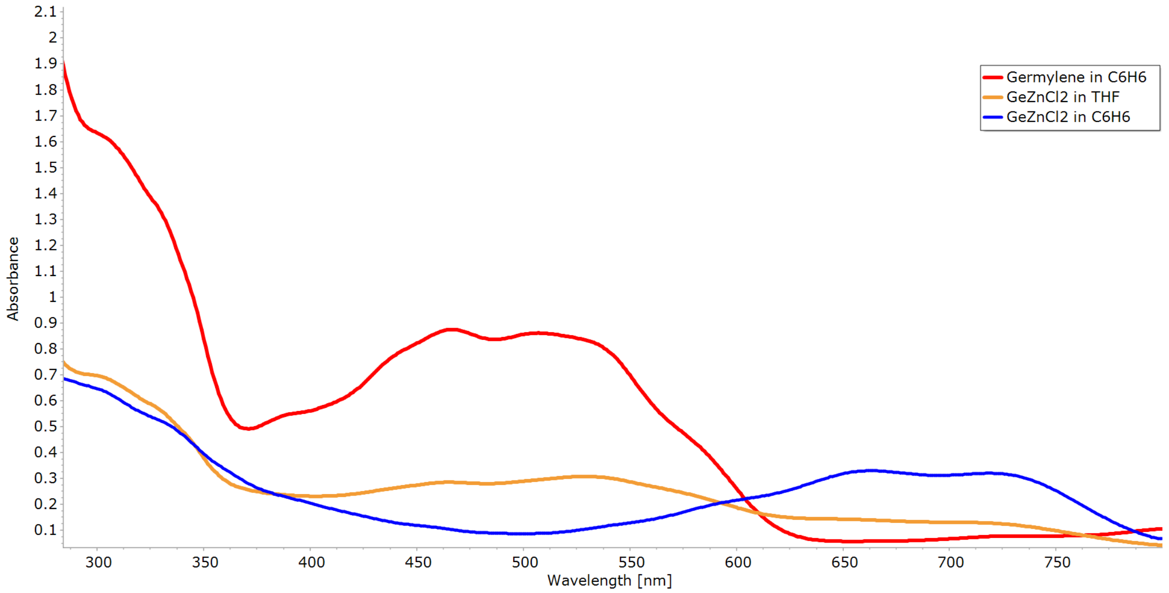

Figure S30: Stacked UV-Vis spectra of **2** in benzene (red trace), **3** in benzene (blue trace), and **3** in THF (yellow trace), recorded at 298 K at a concentration of 0.15 mM.

1.6 Synthesis of ^{dipp}**NBA****GeMg·(THF)$_x$ (**4**):

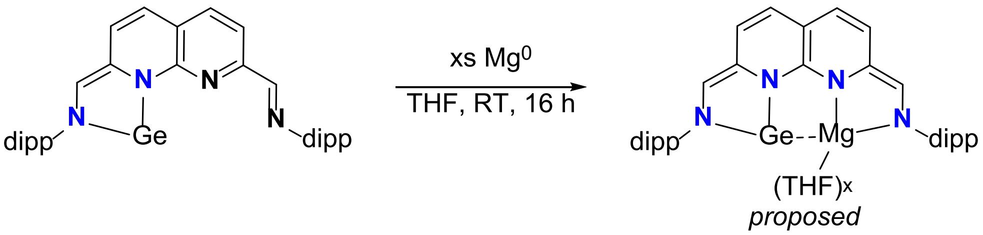

2 **4**

A solution of **2** (20.0 mg, 34.6 µmol, 1.0 equiv) in THF (3 mL) was added to a vigorously stirring suspension of Mg^0 powder (Fine mesh - 325, 2.5 mg, 102.8 µmol, 3.0 equiv) in THF (2 mL). The mixture was stirred for 16 h at ambient temperature, resulting in a colour change to dark green. Subsequently, the resulting mixture was subjected to pipette filtration over a short pad (<0.5 cm) of pre-wetted Celite® and the volatiles of the filtrate were carefully removed until a film formed.* Further reactions with **4** were carried out by dissolving the film in THF and using this as a stock solution.

* **4** is a highly reactive compound that decomposes upon complete removal of the solvent.

^{1}H-NMR (400 MHz, THF-*d*$_8$, 298 K): δ 7.22-7.17 (m, 3H), 6.90 (d, $^3J_{H,H}$ = 7.5 Hz, 2H), 6.75 (t, $^3J_{H,H}$ = 7.5 Hz, 1H), 6.50 (s, 1H), 6.27 (d, $^3J_{H,H}$ = 8.3 Hz, 1H), 5.95 (d, $^3J_{H,H}$ = 8.3 Hz, 1H), 5.43 (d, $^3J_{H,H}$ = 9.1 Hz, 1H), 5.37 (s, 1H), 5.04 (d, $^3J_{H,H}$ = 9.1 Hz, 1H), 3.67 (sept, $^3J_{H,H}$ = 6.9 Hz, 2H), 2.97 (sept, $^3J_{H,H}$ = 6.9 Hz, 2H), 1.18-1.09 (m, 24H).

^{13}C{^{1}H}-NMR (100 MHz, THF-*d*$_8$, 298 K): δ158.1, 155.5, 145.9, 145.9, 143.3, 138.6, 129.3, 128.9, 128.7, 127.6, 1238, 123.3, 121.7, 121.6, 120.4, 114.5, 107.0, 102.6, 28.6, 27.9, 26.7, 26.6, 26.0, 24.4.

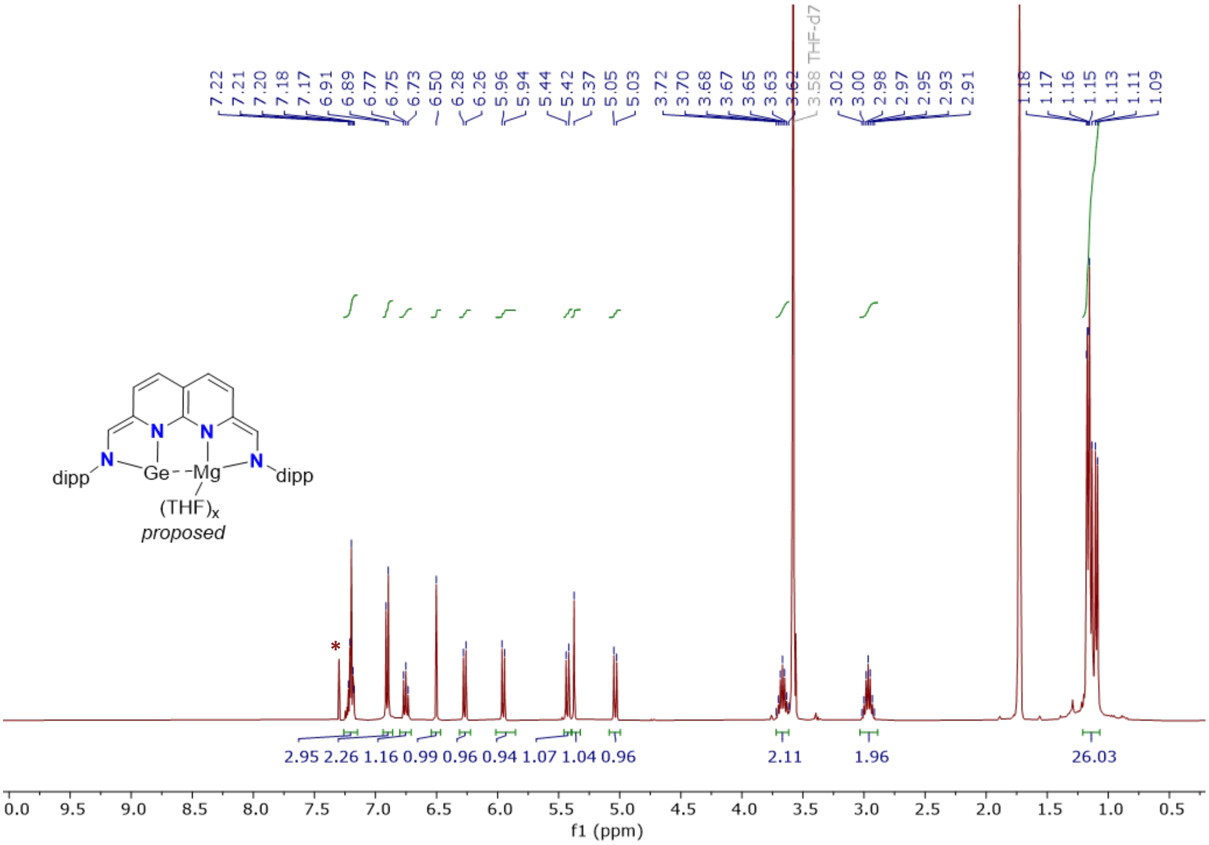

Figure S31: ^{1}H-NMR spectrum of **4** in THF-d_8 at 298 K. Resonance marked with * is attributed to adventitious benzene.

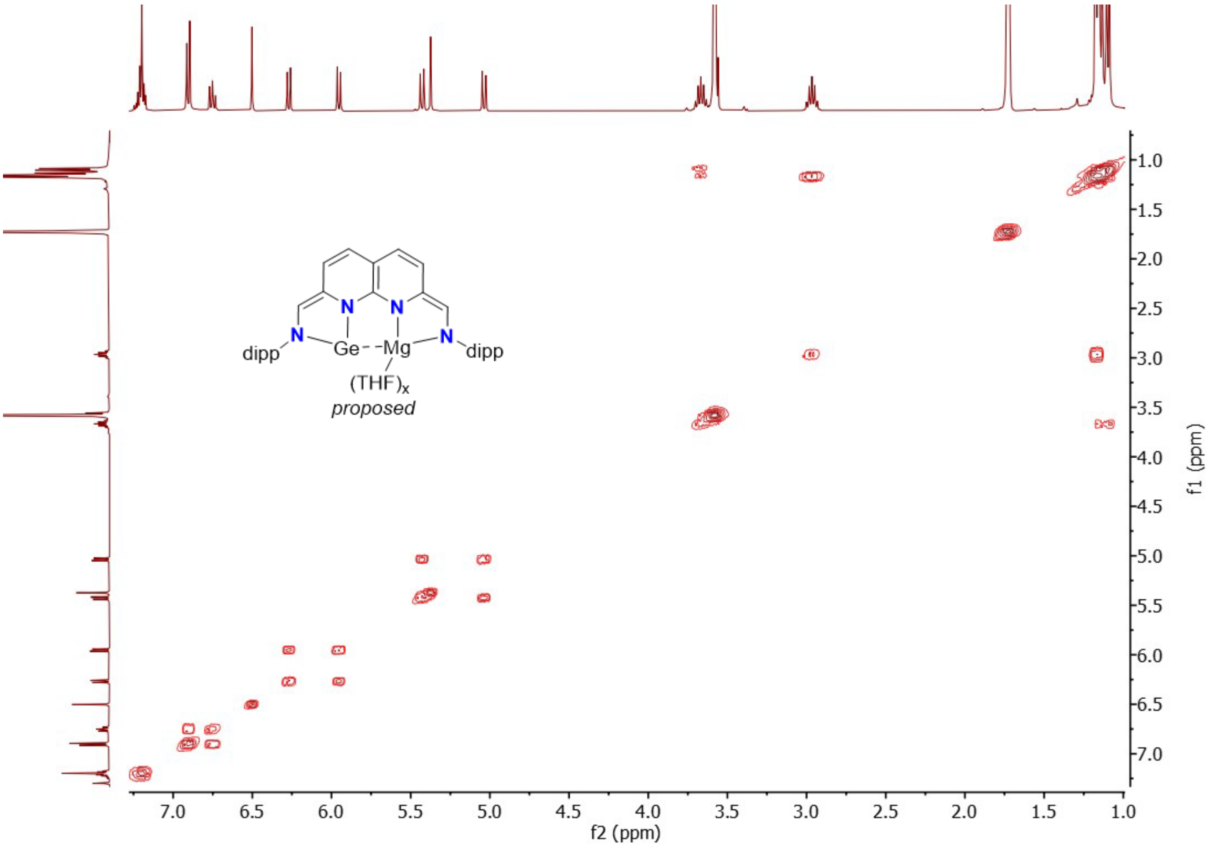

Figure S32: ^{1}H COSY-NMR spectrum of **4** in THF-d_8 at 298 K.

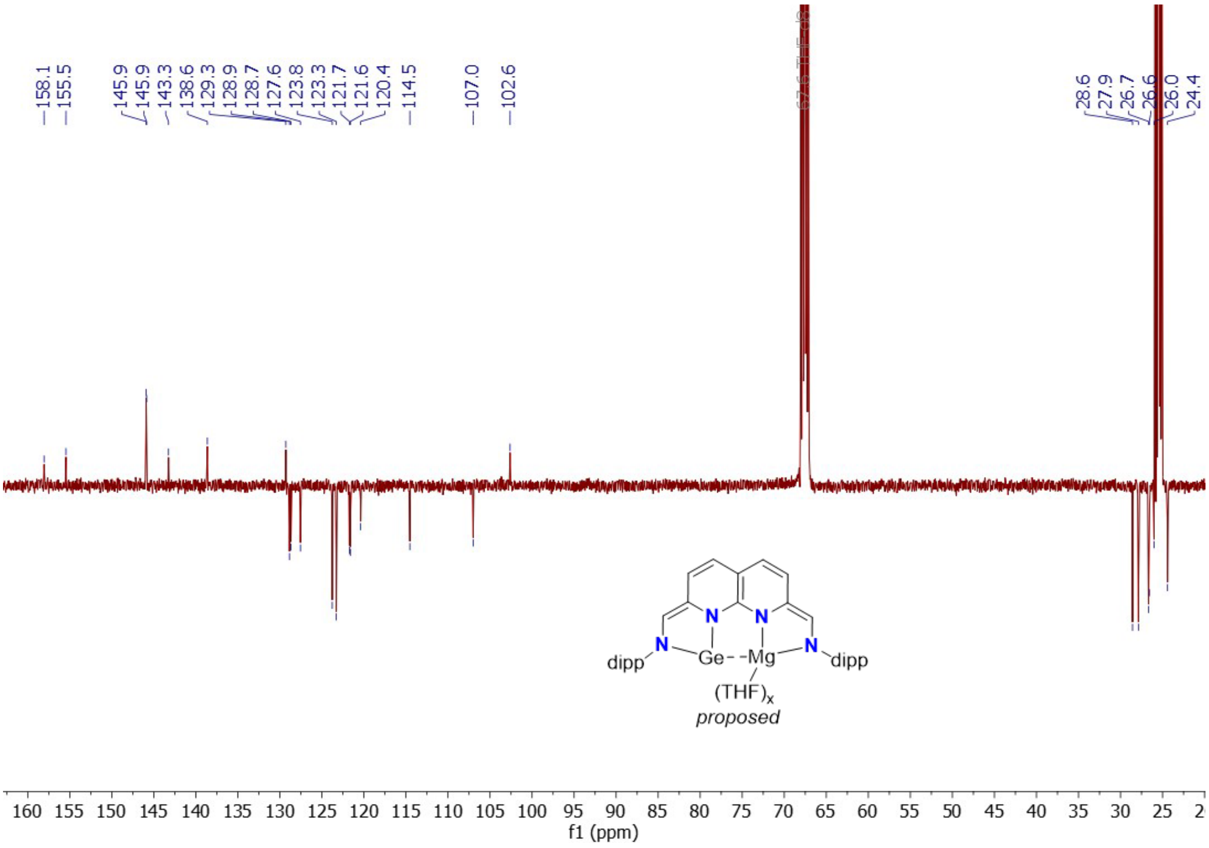

Figure S33: $^{13}C\{^{1}H\}$-NMR (APT) spectrum of **4** in THF-d_8 at 298 K.

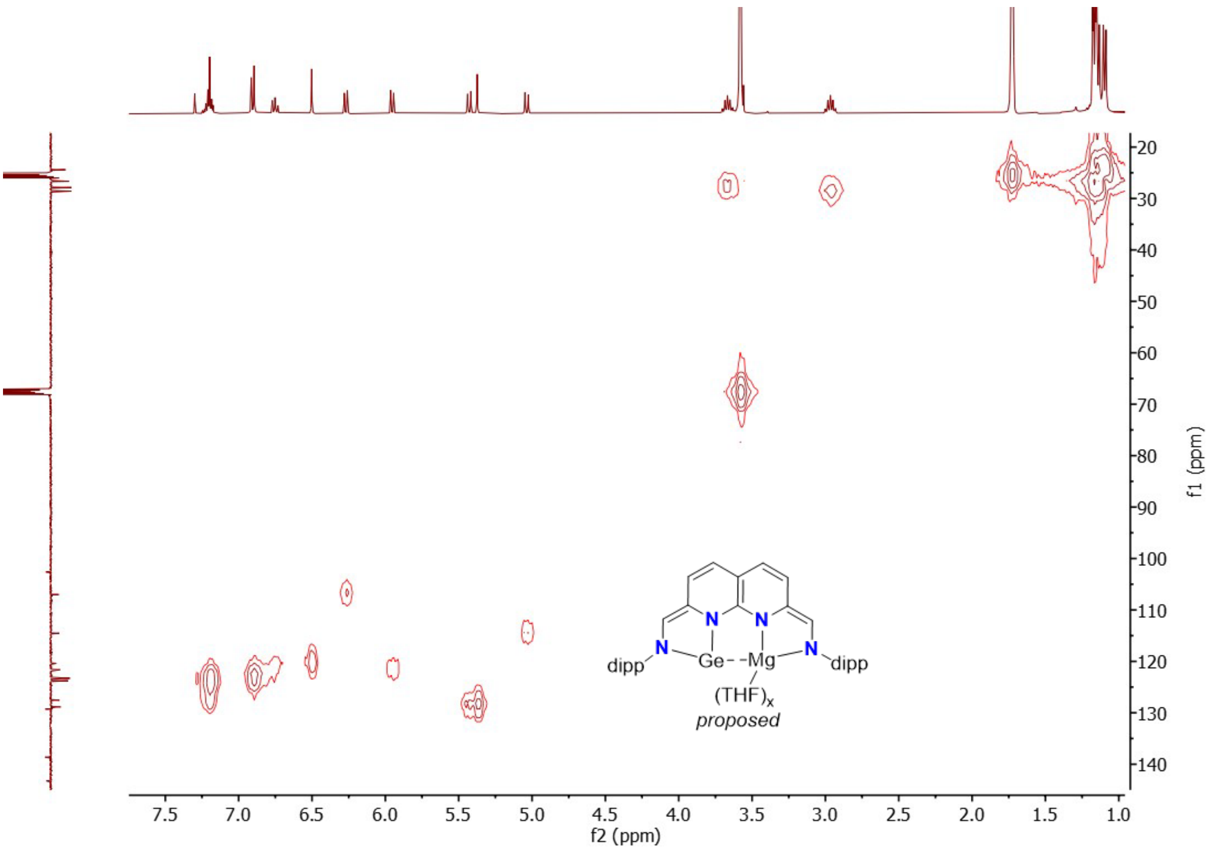

Figure S34: ^{1}H-^{13}C ASAP-HMQC-NMR spectrum of **4** in THF-d_8 at 298 K.

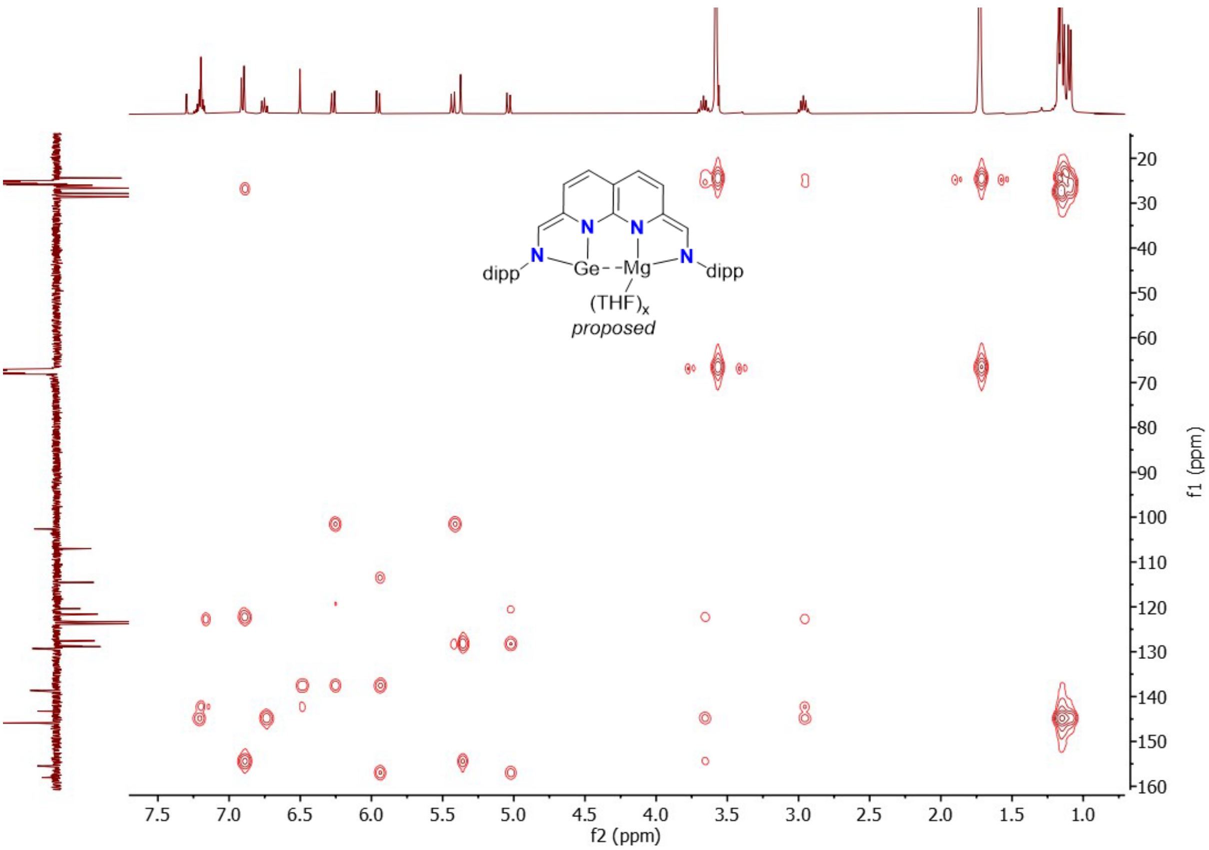

Figure S35: ^{1}H-^{13}C HMBC-NMR spectrum of **4** in THF-d_8 at 298 K.

1.7 Synthesis of ^{dipp}NBA*GeMg·*p*-tolylacetylene (5):

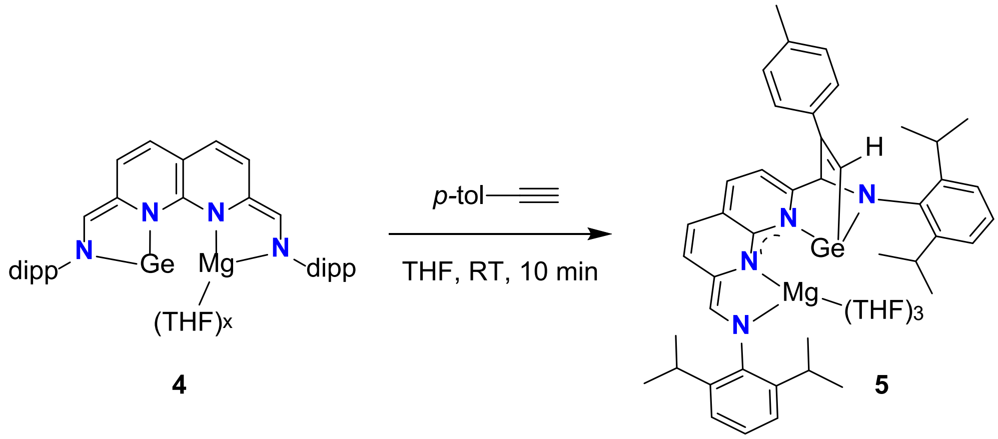

p-Tolylacetylene (1.1 µL, 8.7 µmol, 1.0 equiv) was added by microsyringe to a stirring solution of **4** (8.7 µmol, 1.0 equiv, from stock-solution, see Section 1.6) in THF (1 mL). The mixture was stirred for 10 min at ambient temperature and dried under vacuum to a green film. The film was washed with pentane (2 x 1 mL) and dried in vacuo to a green solid (5.0 mg, 5.4 µmol, 62%). Single crystals suitable for analysis by XRD diffraction were grown from a saturated THF/MTBE solution at –40 °C.

^{1}H-NMR (400 MHz, THF-*d*$_8$, 298 K): δ 7.38 (s, 1H), 7.27 (d, $^3J_{H,H}$ = 8.1 Hz, 2H), 6.97 (d, $^3J_{H,H}$ = 8.1 Hz, 2H), 6.95-6.91 (m, 4H), 6.83 (t, $^3J_{H,H}$ = 7.6 Hz, 1H), 6.67 (t, $^3J_{H,H}$ = 7.6 Hz, 1H), 5.57 (s, 1H), 5.54 (d, $^3J_{H,H}$ = 6.4 Hz, 1H), 5.43 (d, $^3J_{H,H}$ = 8.9 Hz, 1H), 5.17 (d, $^3J_{H,H}$ = 6.4 Hz, 1H), 5.07 (s, 1H), 4.71 (d, $^3J_{H,H}$ = 8.9 Hz, 1H), 3.58 (sept*, 2H), 3.47 (sept, $^3J_{H,H}$ = 6.9 Hz, 2H), 2.23 (s, 3H), 1.24-1.04 (m, 24H).

^{13}C{^{1}H}-NMR (100 MHz, THF-*d*$_8$, 298 K): δ 162.3, 159.6, 154.8, 150.5, 149.7, 147.6, 145.7, 145.5, 138.0, 137.8, 136.5, 131.5, 129.5, 129.1, 126.2, 125.2, 123.9, 123.1, 122.9, 121.5, 118.0, 110.8, 103.9, 81.0, 27.9, 27.8, 26.2, 26.0, 24.5, 23.8, 21.2.

* Resonance overlapped by solvent.

ATR-IR (cm^{-1}): 2958 (s), 2923 (s), 2865 (m), 1571 (m), 1546 (w), 1506 (w), 1459 (w), 1427 (s), 1402 (w), 1382 (w), 1323 (w), 1295 (w), 1271 (w), 1253 (w), 1232 (w), 1203 (s), 1177 (w), 1160 (w), 1054 (s), 1034 (s).

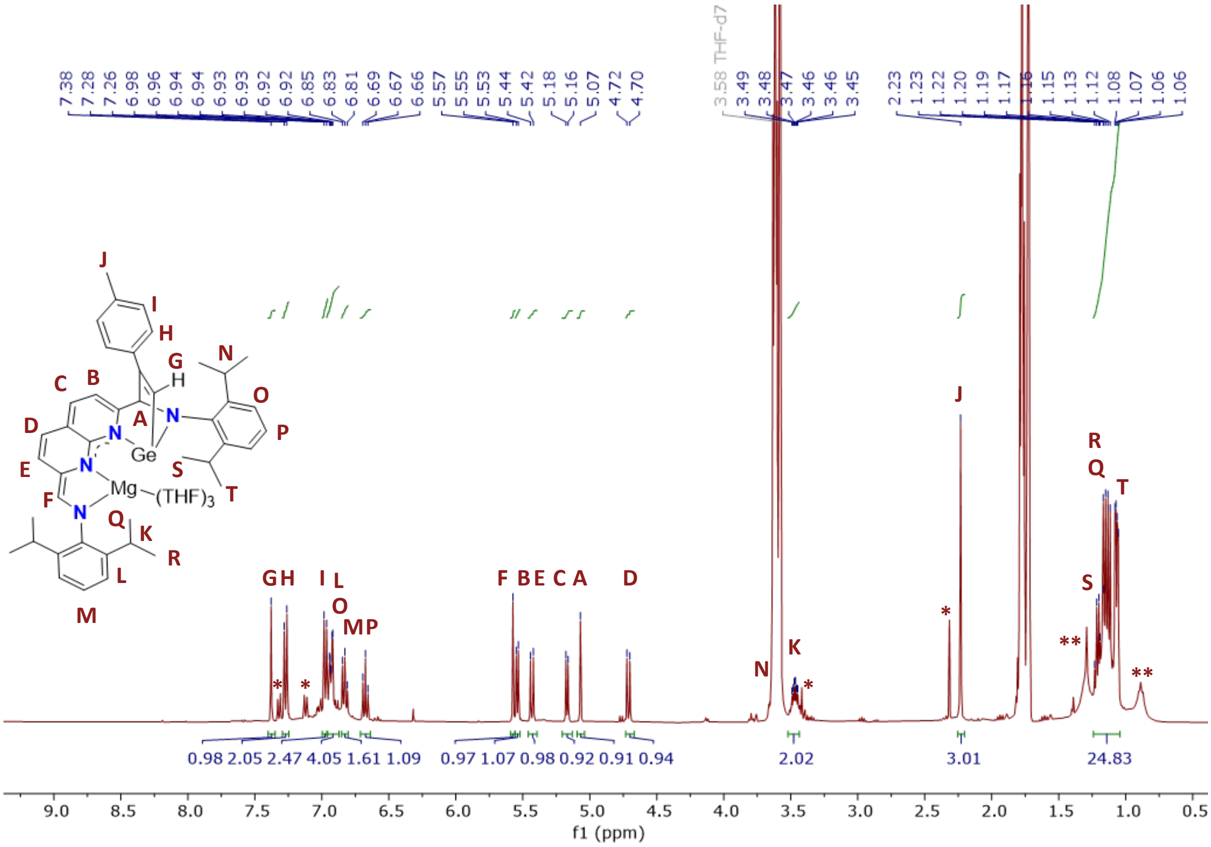

Figure S36: ^{1}H-NMR spectrum of **5** in THF-d_8 at 298 K. Resonances marked with * are attributed to residual *p*-tolylacetylene, the resonances marked with ** are attributed to adventitious pentane.

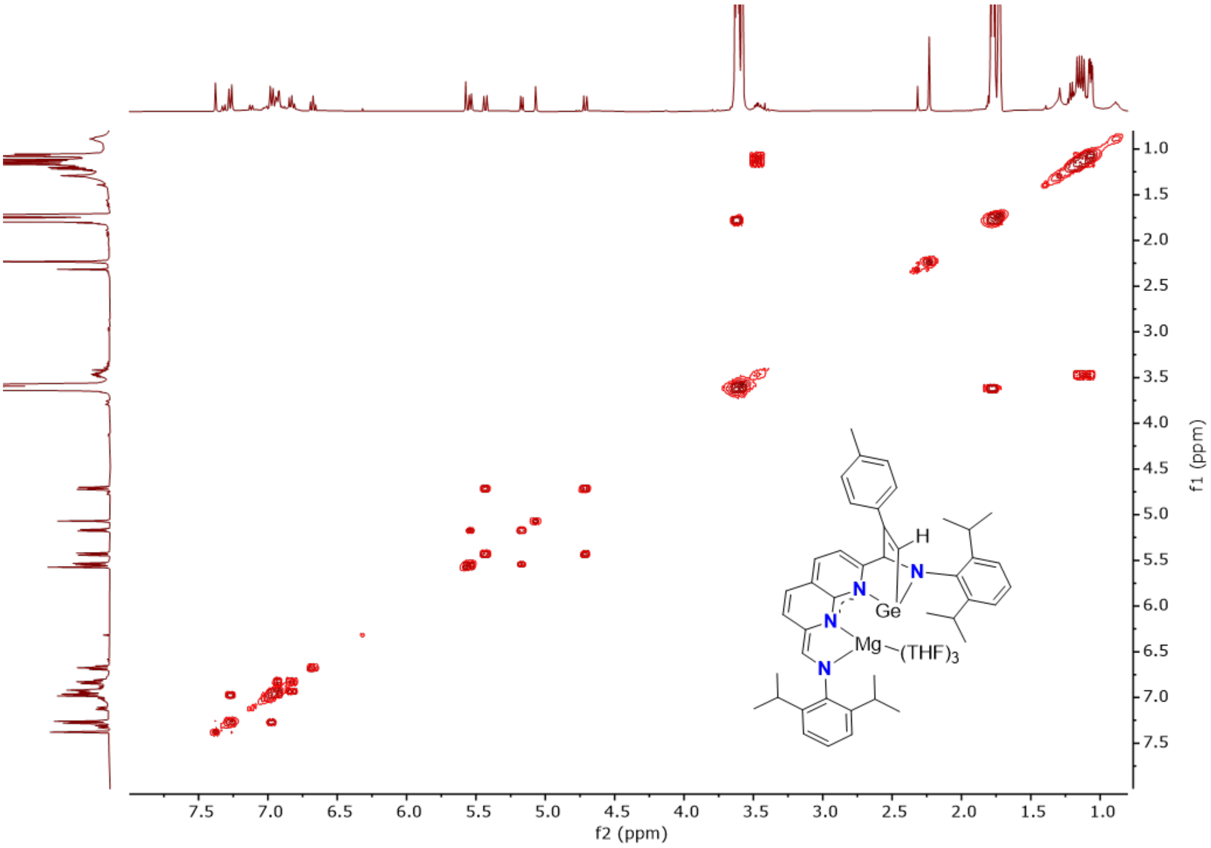

Figure S37: ^{1}H COSY-NMR spectrum of **5** in THF-d_8 at 298 K.

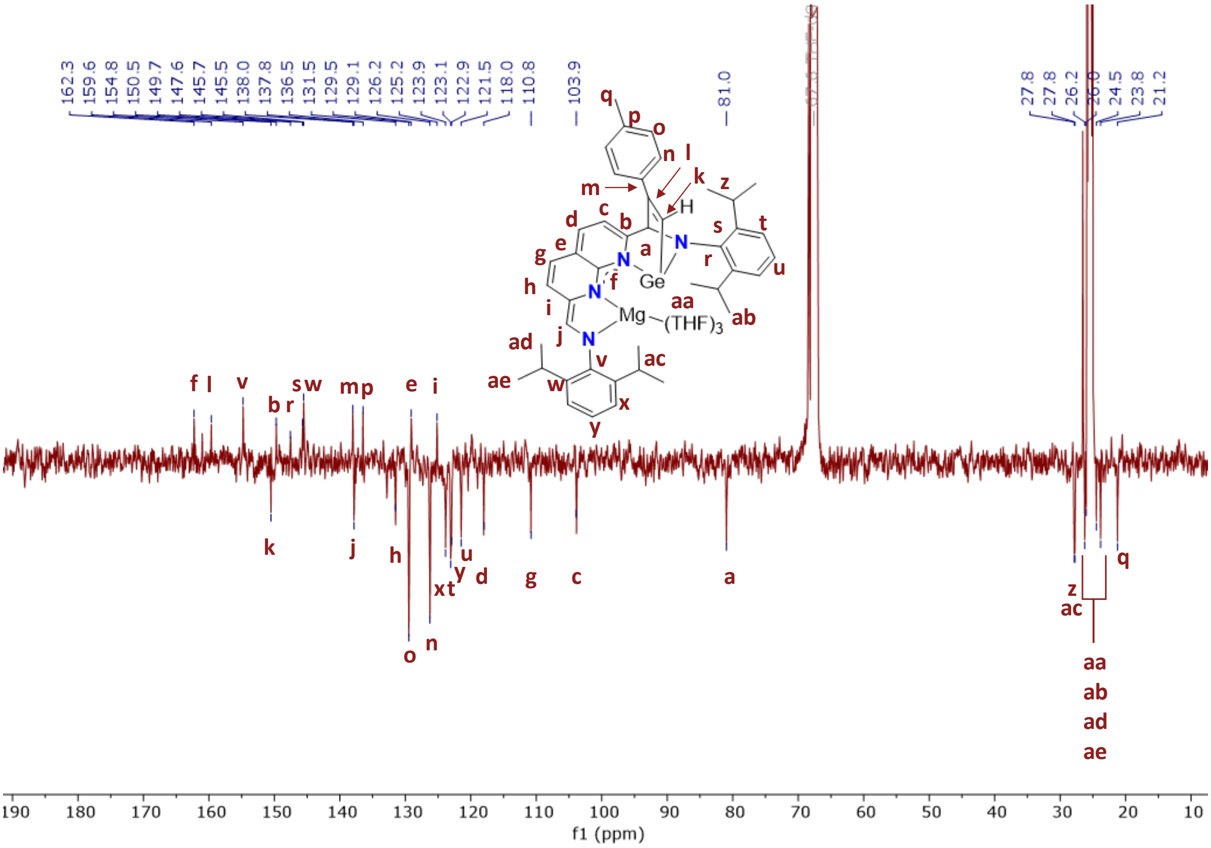

Figure S38: $^{13}C\{^{1}H\}$-NMR (APT) spectrum of **5** in THF-d_8 at 298 K.

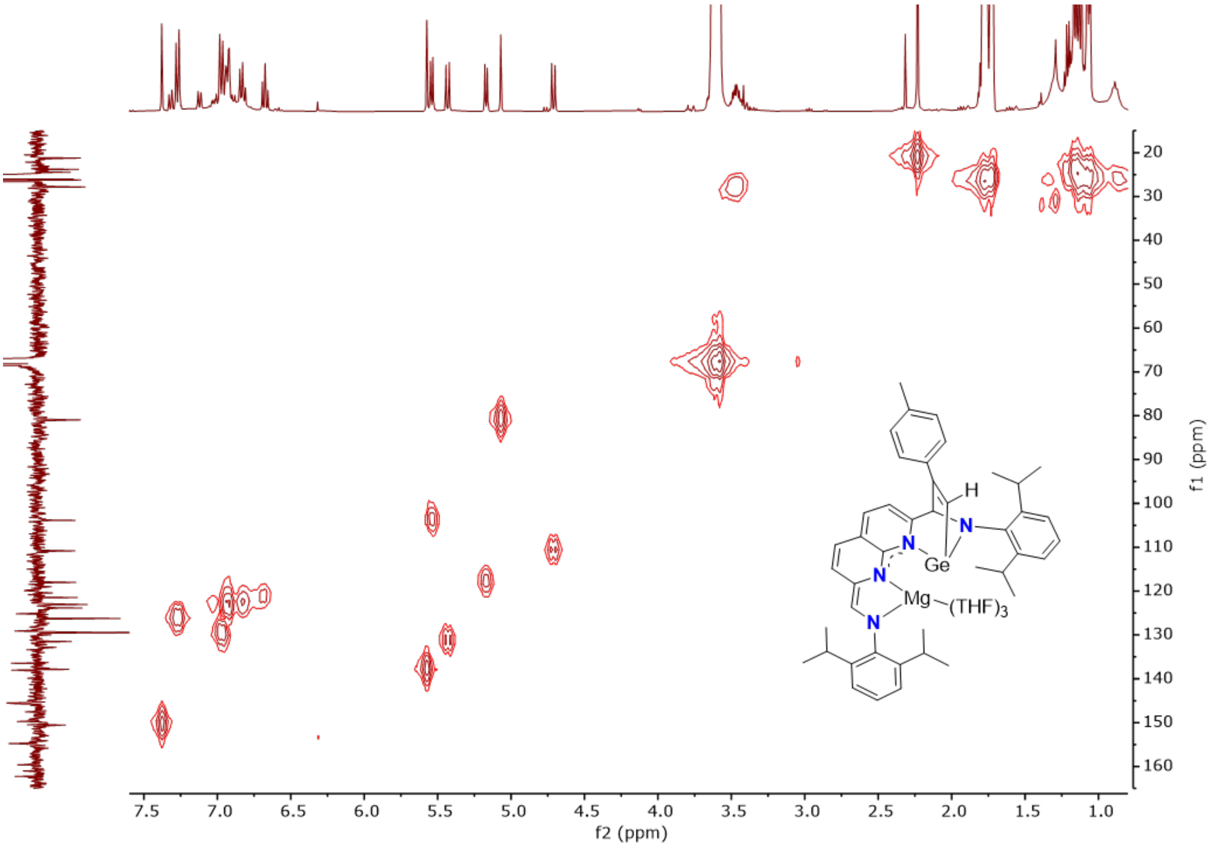

Figure S39: ^{1}H-^{13}C ASAP HMQC-NMR spectrum of **5** in THF-d_8 at 298 K.

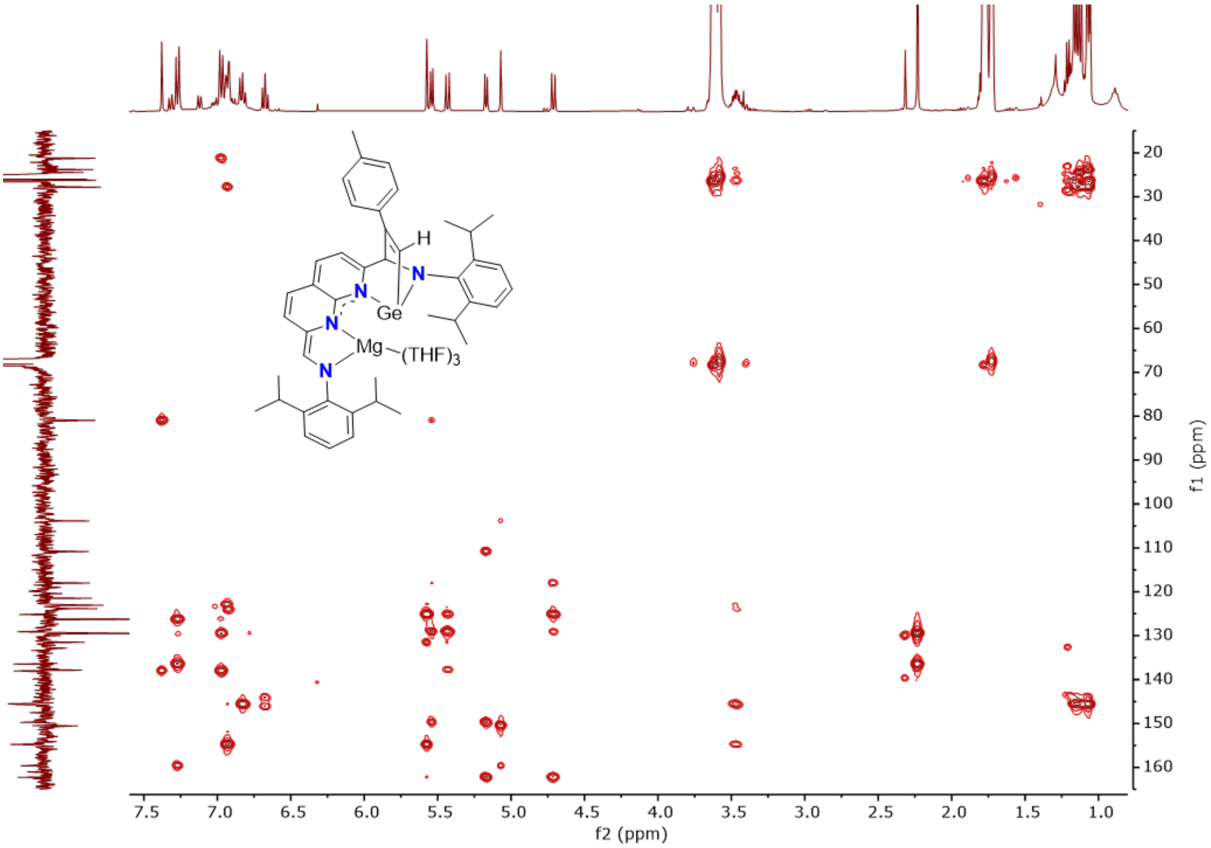

Figure S40: ^{1}H-^{13}C HMBC-NMR spectrum of **5** in THF-d_8 at 298 K.

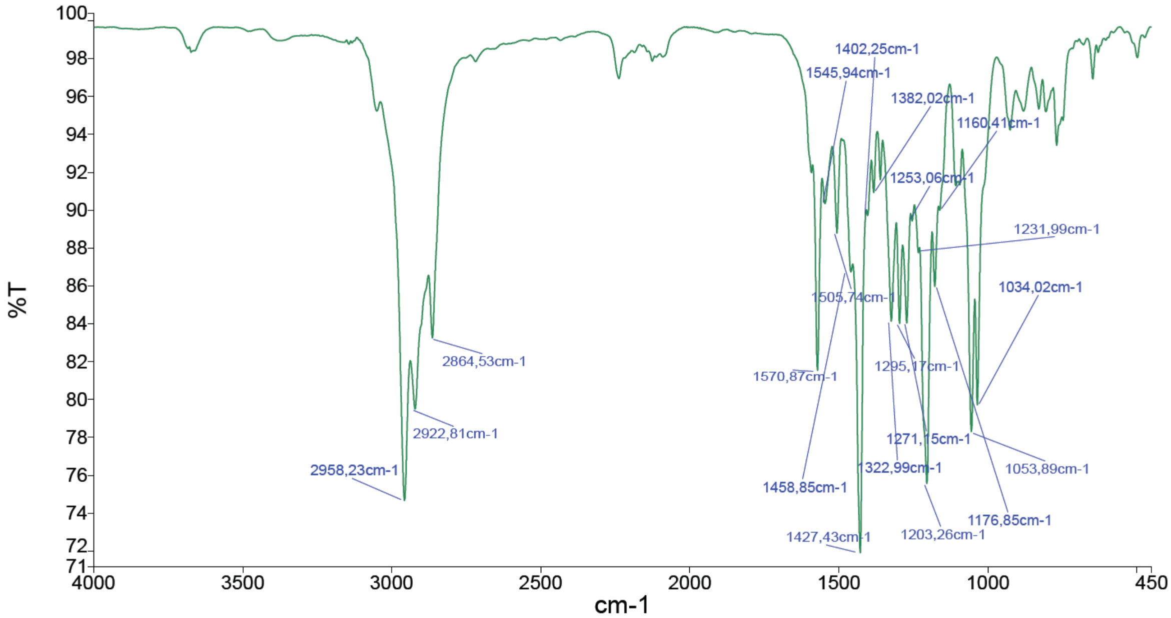

Figure S41: ATR-IR spectrum of **5** measured as a film under N_2 flow at 298 K.

1.8 Synthesis of ^{dipp}**NBA***GeMg·diphenylacetylene (**6**):

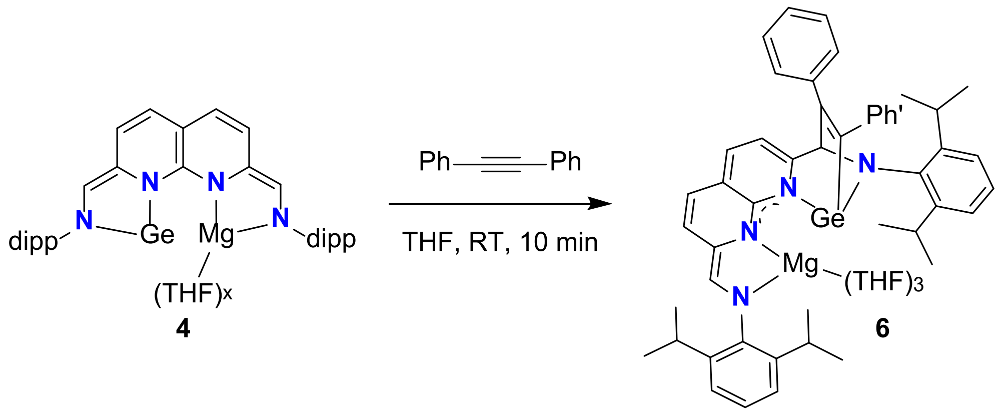

Diphenylacetylene (1.6 mg, 8.7 µmol, 1.0 equiv) dissolved in minimal THF (<0.5 mL) was added to a stirring solution of **4** (8.7 µmol, 1.0 equiv, from stock-solution, see Section 1.6) in THF (1 mL). The mixture was stirred for 10 min at ambient temperature and dried under vacuum to a green film. The film was extracted with pentane (2 x 1 mL) and dried in vacuo to a dark-green/blueish solid (5.8 mg, 5.8 µmol, 67%).

^{1}H-NMR (400 MHz, THF-d_8, 298 K): δ 7.20-7.13 (m, 4H), 7.06-6.87 (m, 10H), 6.82 (dd, $^3J_{H,H}$ = 7.6 Hz, $^3J_{H,H}$ = 7.4 Hz, 1H), 6.69 (dd, $^3J_{H,H}$ = 7.6 Hz, $^3J_{H,H}$ = 7.6 Hz, 1H), 5.76 (d, $^3J_{H,H}$ = 6.4 Hz, 1H), 5.53 (s, 1H), 5.43 (d, $^3J_{H,H}$ = 8.9 Hz, 1H), 5.29 (d, $^3J_{H,H}$ = 6.4 Hz, 1H), 4.87 (s, 1H), 4.75 (d, $^3J_{H,H}$ = 8.9 Hz, 1H), 3.97 (sept, $^3J_{H,H}$ = 6.7 Hz, 1H), 3.58 (sept*, 1H), 3.44 (sept, $^3J_{H,H}$ = 6.9 Hz, 1H), 3.25 (sept, $^3J_{H,H}$ = 6.9 Hz, 1H), 1.32 (d, $^3J_{H,H}$ = 6.9 Hz, 3H), 1.25 (d, $^3J_{H,H}$ = 6.6 Hz, 3H), 1.16 (d, $^3J_{H,H}$ = 6.6 Hz, 3H), 1.10-1.04 (m, 12 H), 1.00 (d, $^3J_{H,H}$ = 6.9 Hz, 3H).

^{13}C{^{1}H}-NMR (100 MHz, THF-d_8, 298 K): δ 165.8, 162.7, 154.7, 152.7, 150.4, 149.6, 145.8, 145.5, 144.8, 143.1, 141.6, 138.8, 131.8, 129.4, 129.2, 129.0, 128.4, 128.3, 126.2, 125.6, 124.7, 124.5, 123.6, 123.3, 123.2, 121.6, 117.8, 110.7, 104.4, 85.4, 31.4, 28.7, 27.7, 27.7, 26.6, 26.4, 26.0, 25.8*, 25.6*, **, 24.1, 23.7. ***

* Resonance partially overlapped by solvent.
** Resonance in this region absent due to overlap by solvent which is in different phase.
*** 3 carbon resonances in the aromatic region are missing, most likely due to overlap with the other resonances in this region. The corresponding proton resonances overlap to severely to resolve the resonances with 2D-NMR spectroscopy. Despite this discrepancy, the integration of the ^{1}H resonances to the appropriate value and the observed cross-peaks in the ^{1}H-^{13}C HMBC spectrum clearly demonstrate activation of the internal alkyne.

ATR-IR (cm^{-1}): 2961 (m), 2926 (m), 2864 (m), 1573 (m), 1456 (m), 1429 (s), 1395 (m), 1382 (m), 1361 (w), 1349 (w), 1322 (m), 1296 (m), 1272 (m), 1234 (m), 1205(s), 1176 (m), 1151 (m), 1115(s), 1055 (s), 1033 (s), 800 (m), 756 (s), 691 (m).

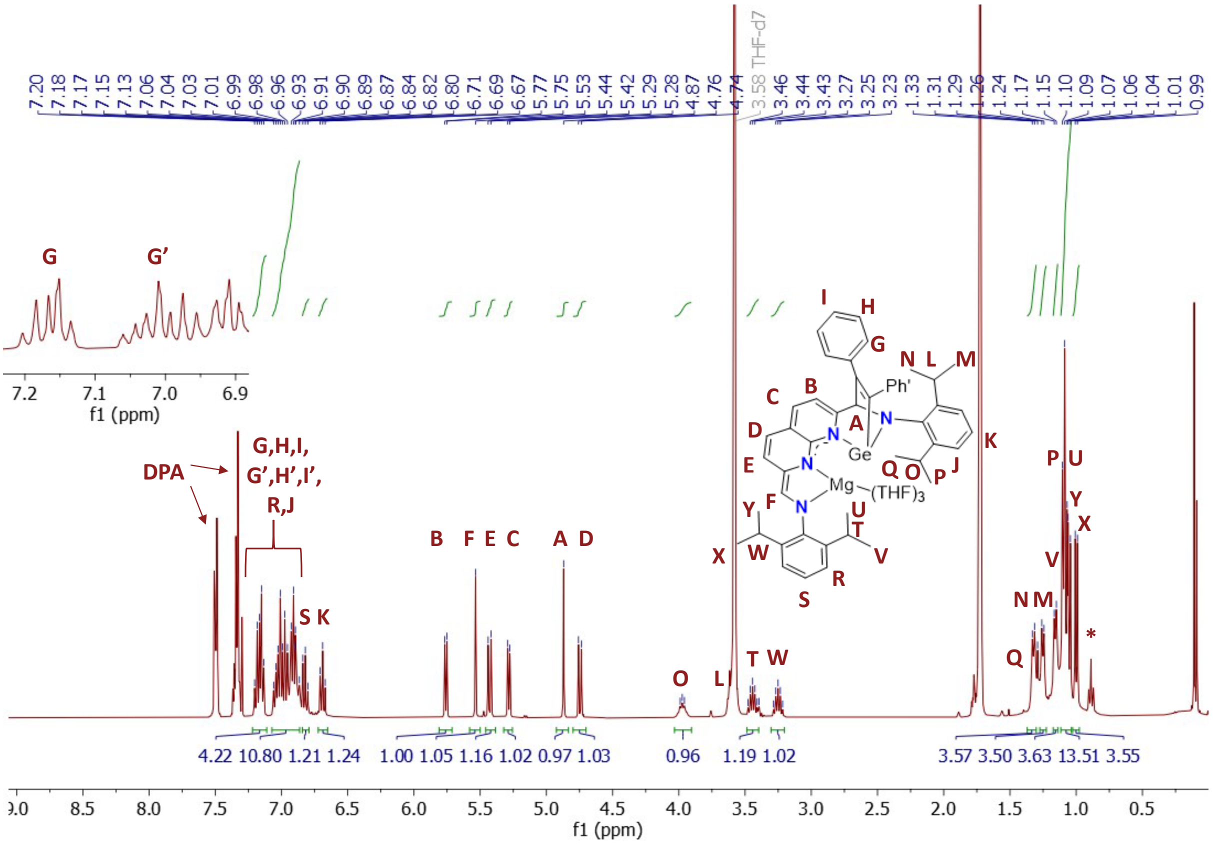

Figure S42: ^{1}H-NMR spectrum of **6** in THF-d_8 at 298 K. Resonances marked with * are attributed to residual pentane. The inset shows the zoomed-in region of the aromatic dipp and phenyl resonances.

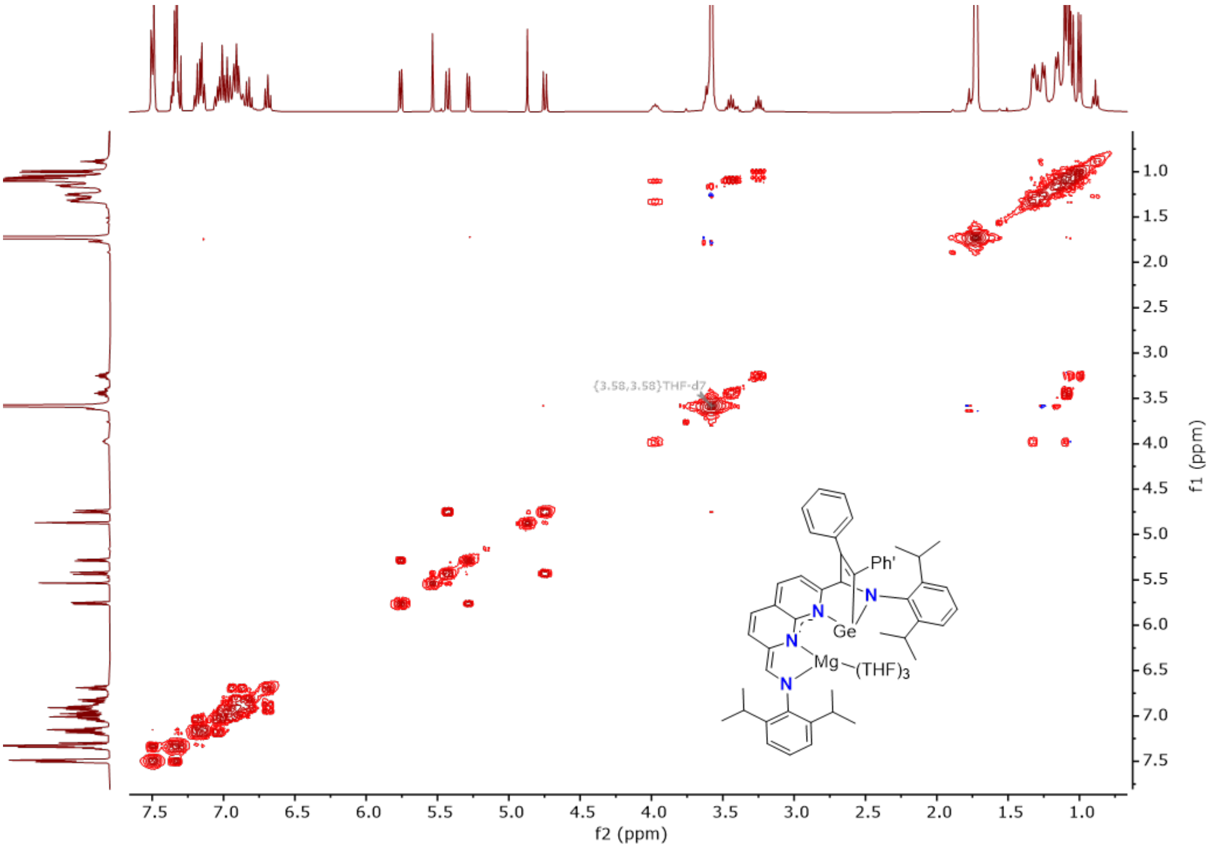

Figure S43: ^{1}H COSY-NMR spectrum of **6** in THF-d_8 at 298 K.

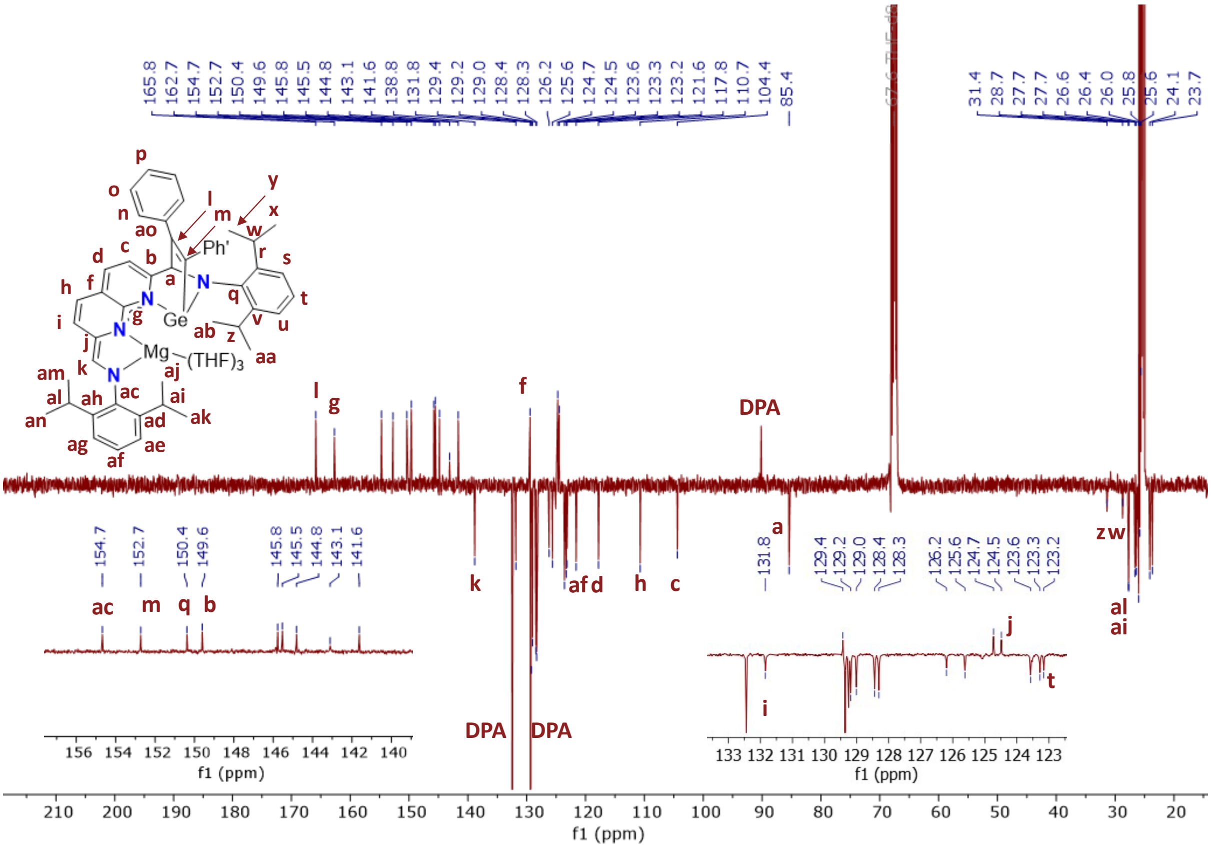

Figure S44: $^{13}C\{^{1}H\}$-NMR (APT) spectrum of **6** in THF-d_8 at 298 K. The insets show the zoomed-in regions between 140-156 and 123-133 ppm. Only resonances that could be unambiguously assigned were labelled. Resonances attributed to free DPA were not peak-picked but labelled for clarity.

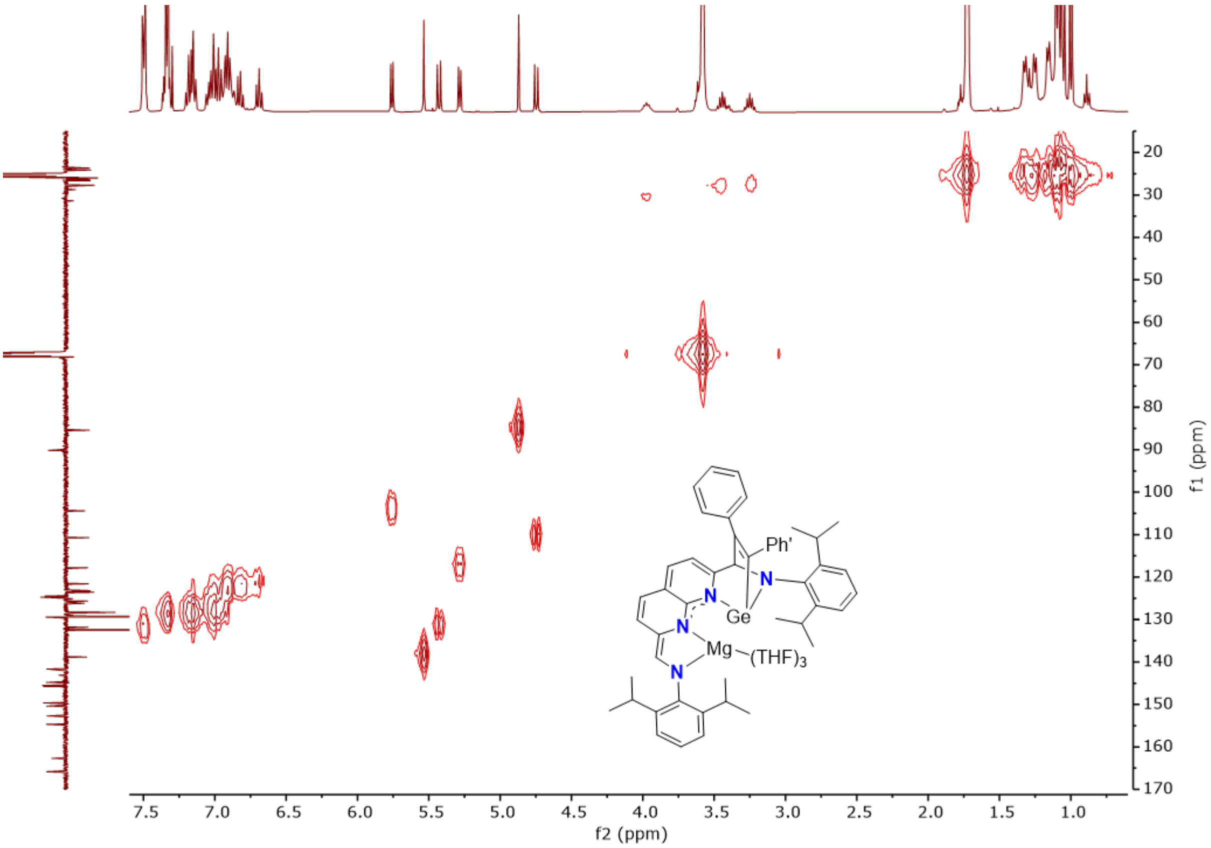

Figure S45: ^{1}H-^{13}C ASAP HMQC-NMR spectrum of **6** in THF-d_8 at 298 K.

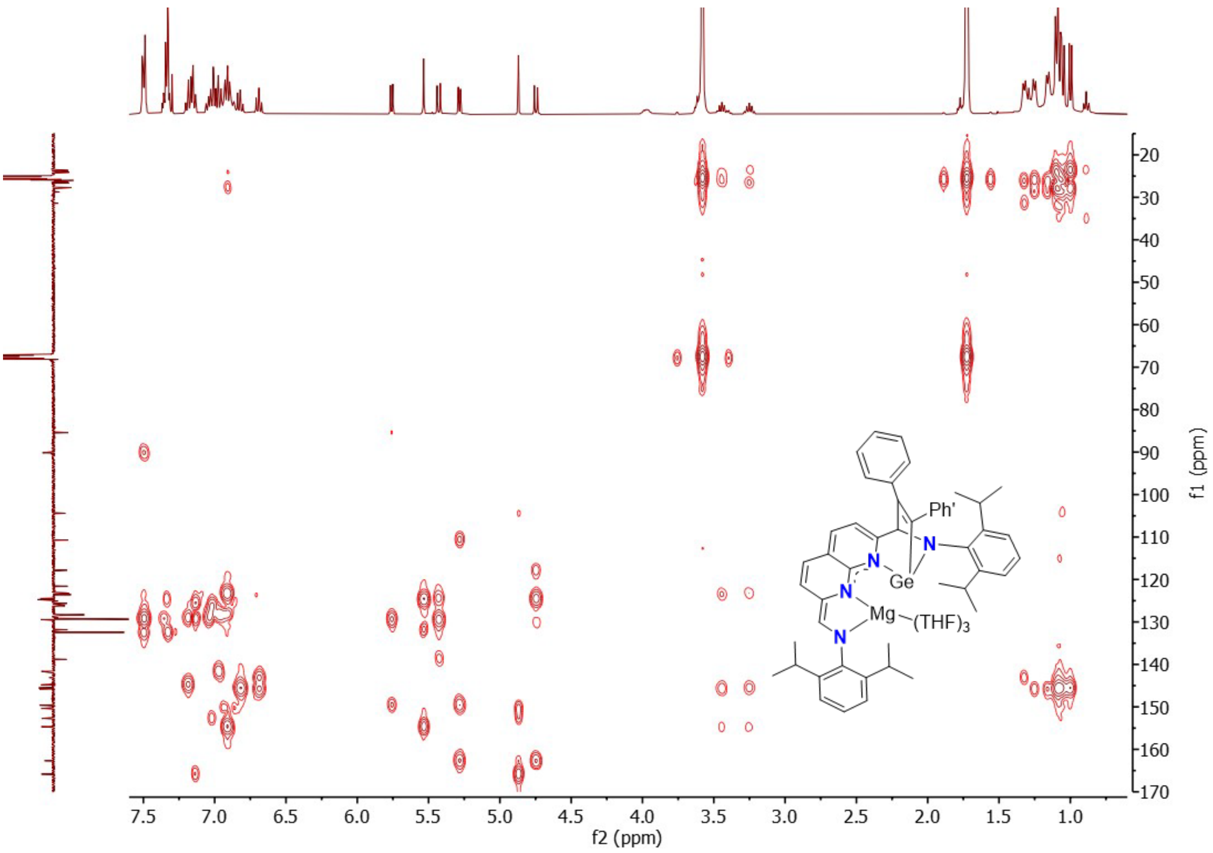

Figure S46: ^{1}H-^{13}C HMBC-NMR spectrum of **6** in THF-d_8 at 298 K.

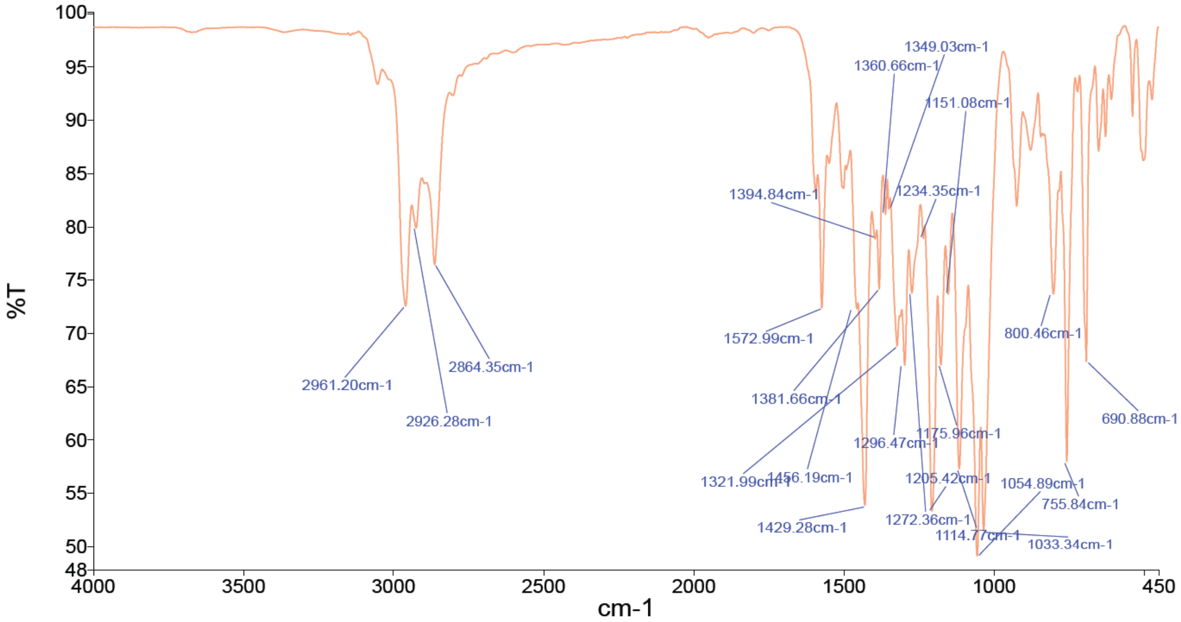

Figure S47: ATR-IR spectrum of **6** measured as a film under N_2 flow at 298 K.

1.9 Synthesis of ^{dipp}**NBA***GeMg·styrene (**7**):

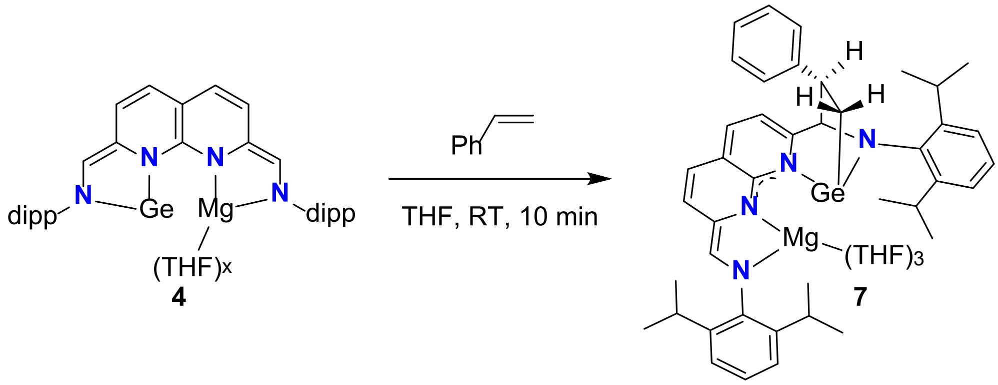

Styrene (0.9 µL, 8.7 µmol, 1.0 equiv) was added by microsyringe to a stirring solution of **4** (8.7 µmol, 1.0 equiv, from stock-solution, see Section 1.6) in THF (1 mL). The mixture was stirred for 10 min at ambient temperature and dried under vacuum to a blue film. The film was extracted with pentane (3 x 1 mL) and dried in vacuo to a blue solid (3.3 mg, 3.6 µmol, 41%).

^{1}H-NMR (400 MHz, THF-d_8, 298 K): δ 7.18 (d, $^{3}J_{H,H}$ = 7.8 Hz, 2H), 7.11 (dd, $^{3}J_{H,H}$ = 7.8 Hz, $^{3}J_{H,H}$ = 7.4 Hz, 2H), 6.82 (dd, $^{3}J_{H,H}$ = 7.6 Hz, $^{3}J_{H,H}$ = 7.4 Hz, 1H), 6.99 (t, $^{3}J_{H,H}$ = 7.4 Hz, 1H), 6.94-6.91 (m, 4 H), 6.82 (dd, $^{3}J_{H,H}$ = 7.6 Hz, $^{3}J_{H,H}$ = 7.5 Hz, 1H), 6.74 (dd, $^{3}J_{H,H}$ = 7.6 Hz, $^{3}J_{H,H}$ = 7.6 Hz, 1H), 5.55 (s, 1H), 5.43 (d, $^{3}J_{H,H}$ = 8.9 Hz, 1H), 5.07 (d, $^{3}J_{H,H}$ = 6.5 Hz, 1H), 4.66 (d, $^{3}J_{H,H}$ = 6.5 Hz, 1H), 4.64 (d, $^{3}J_{H,H}$ = 8.9 Hz, 1H), 4.00 (d, $^{3}J_{H,H}$ = 4.0 Hz, 1H), 3.47 (sept*, 4H), 2.96 (ddd, $^{3}J_{H,H}$ = 9.2 Hz, $^{3}J_{H,H}$ = 9.1 Hz, $^{3}J_{H,H}$ = 4.0 Hz, 1H), 1.44 (dd, $^{2}J_{H,H}$ = 12.1 Hz, $^{3}J_{H,H}$ = 9.2 Hz, 1H), 1.25 (d, $^{3}J_{H,H}$ = 6.6 Hz, 3H), 1.16 (d, $^{3}J_{H,H}$ = 6.6 Hz, 3H), 1.16-1.04 (m, 24 H), 0.68 (dd, $^{2}J_{H,H}$ = 12.1 Hz, $^{3}J_{H,H}$ = 9.1 Hz, 1H).

^{13}C{^{1}H}-NMR (100 MHz, THF-d_8, 298 K): δ 161.7, 154.9, 150.2, 149.4, 146.3, 145.7, 145.4, 137.3, 131.9, 129.9, 129.7, 128.1, 125.9, 125.5, 123.9, 123.0, 122.8, 121.3, 118.5, 111.0, 105.8, 81.2, 49.7, 30.6, 27.8, 27.7, 26.2, 26.0, 24.6, 23.7.

*Overlapping resonances.

ATR-IR (cm^{-1}): 2960 (m), 2864 (w), 1574 (m), 1457 (m), 1429 (s), 1382 (m), 1360 (w), 1321 (m), 1296 (m), 1272 (m), 1234 (m), 1205 (s), 1176 (m), 1156 (m), 1114 (m), 1098 (m), 1053 (s), 1032 (s), 935 (m), 874 (m), 844 (w), 798 (m), 757 (m), 698 (m), 656 (w), 644 (m), 625 (w), 616 (w), 496 (m), 471 (m).

1D and 2D NMR Characterisation:

Analysis of the ^{1}H-NMR spectrum of **7** in THF-d_8 revealed four naphthyridine doublets in the region δ = 5.44-4.63 ppm, similar to previous observations. In contrast to the compounds reported above, only one methine singlet was observed (δ = 5.55 ppm). Through ^{1}H-^{1}H COSY-NMR analysis, the remaining methine resonance was identified as a doublet at δ = 4.00 ppm ($^3J_{H,H}$ = 3.9 Hz), coupling to a doublet of doublets of doublets ($^3J_{H,H}$ = 9.2, 9.1, 3.9 Hz) at δ = 2.96 ppm, which we assign to the benzylic proton of the activated styrene motif. The activation, and hence loss of the alkene character of the styrene motif, was evident from the chemical shift of the two terminal protons, which were shifted significantly upfield to δ = 1.44 and 0.68 ppm (dd, $^2J_{H,H}$ = 12.1 Hz). Analysis of the 2D-NOESY NMR spectrum (Figure S52) revealed NOEs of the latter resonance with the other terminal proton and a doublet at δ = 7.18 ppm integrating to two protons, which we assign to the ortho protons of the phenyl ring of the activated styrene motif. This resonance at 7.18 ppm, also shows a NOE with one of the naphthyridine doublets at 4.66 ppm, showing a close proximity between the ortho protons of the phenyl and those on the naphthyridine backbone. Combined with the observation that both for the benzylic proton resonance at δ = 2.96 ppm and the terminal proton resonance at δ = 1.44 ppm, NOE cross-peaks could be found with one of the overlapping methine resonances of the dipp substituents δ = 3.47 ppm, we propose that the diastereomer with the phenyl group pointing towards the naphthyridine backbone is formed selectively.

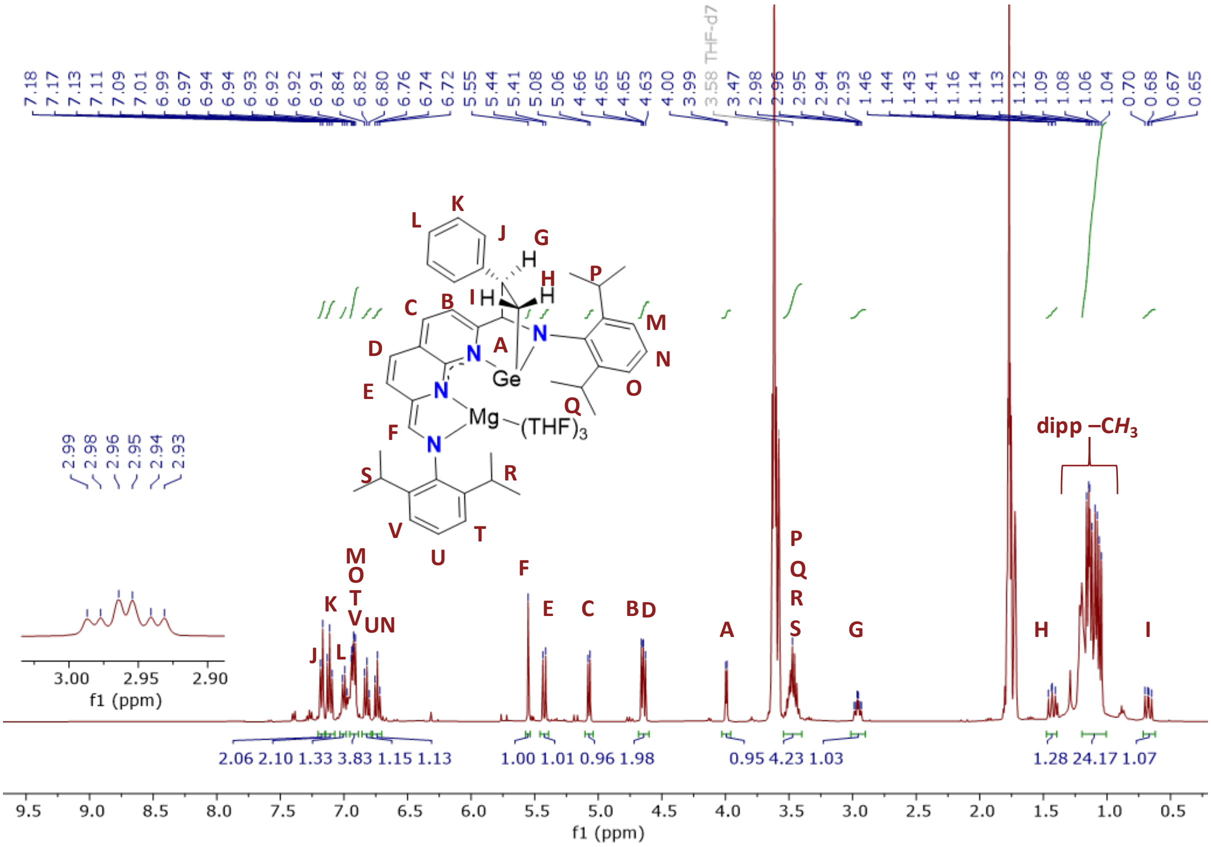

Figure S48: ^{1}H-NMR spectrum of **7** in THF-d_8 at 298 K. The inset shows the zoomed-in region of the benzylic resonances.

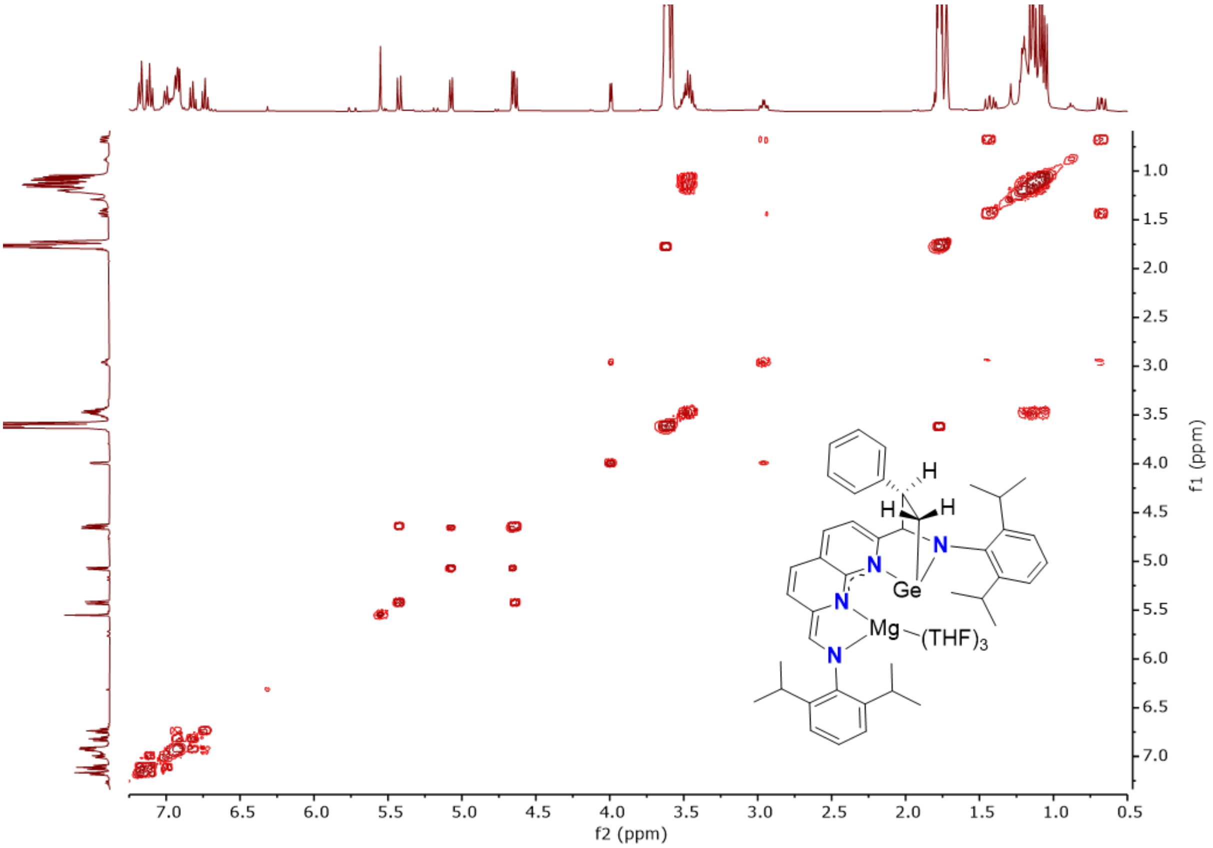

Figure S49: ^{1}H COSY-NMR spectrum of **7** in THF-d_8 at 298 K.

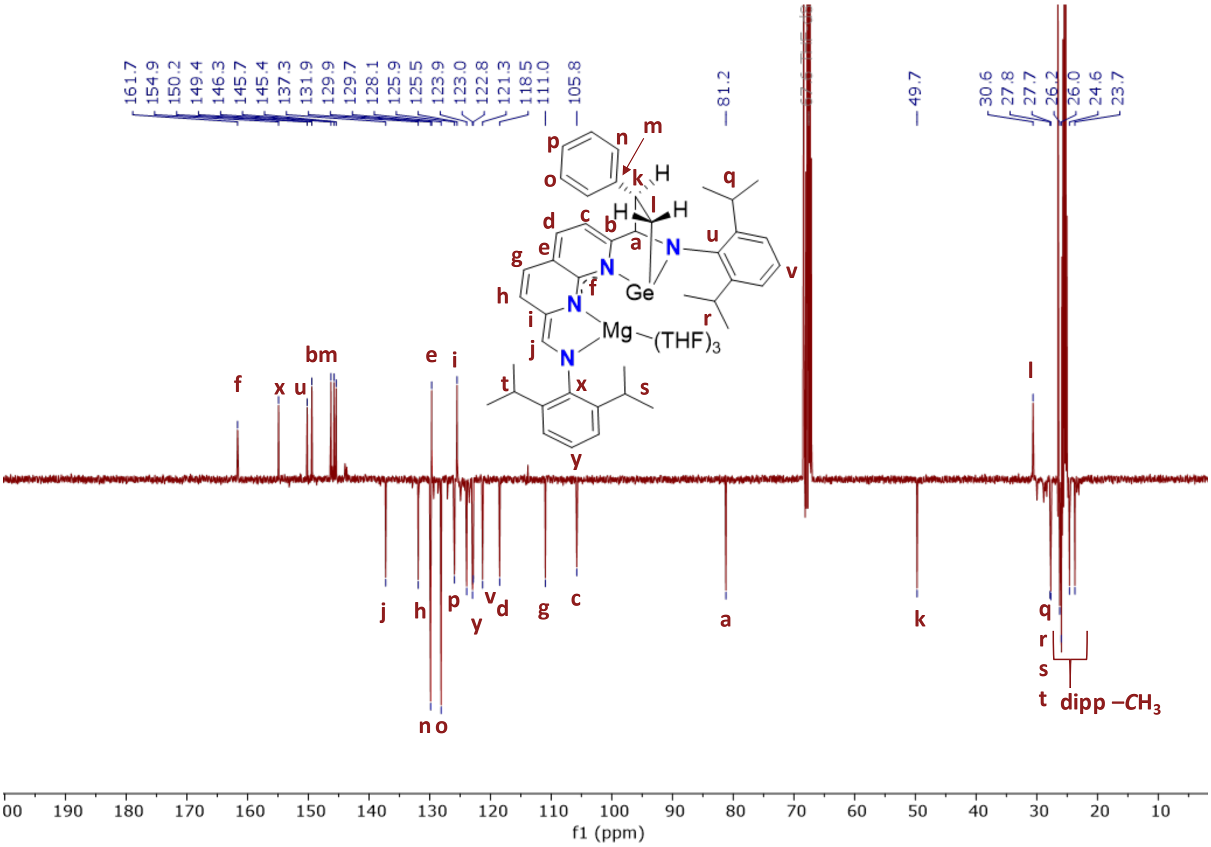

Figure S50: ^{13}C{^{1}H}-NMR (APT) spectrum of **7** in THF-d_8 at 298 K. Only resonances that could be unambiguously assigned were labelled. The number of resonances detected is consistent with the proposed structure.

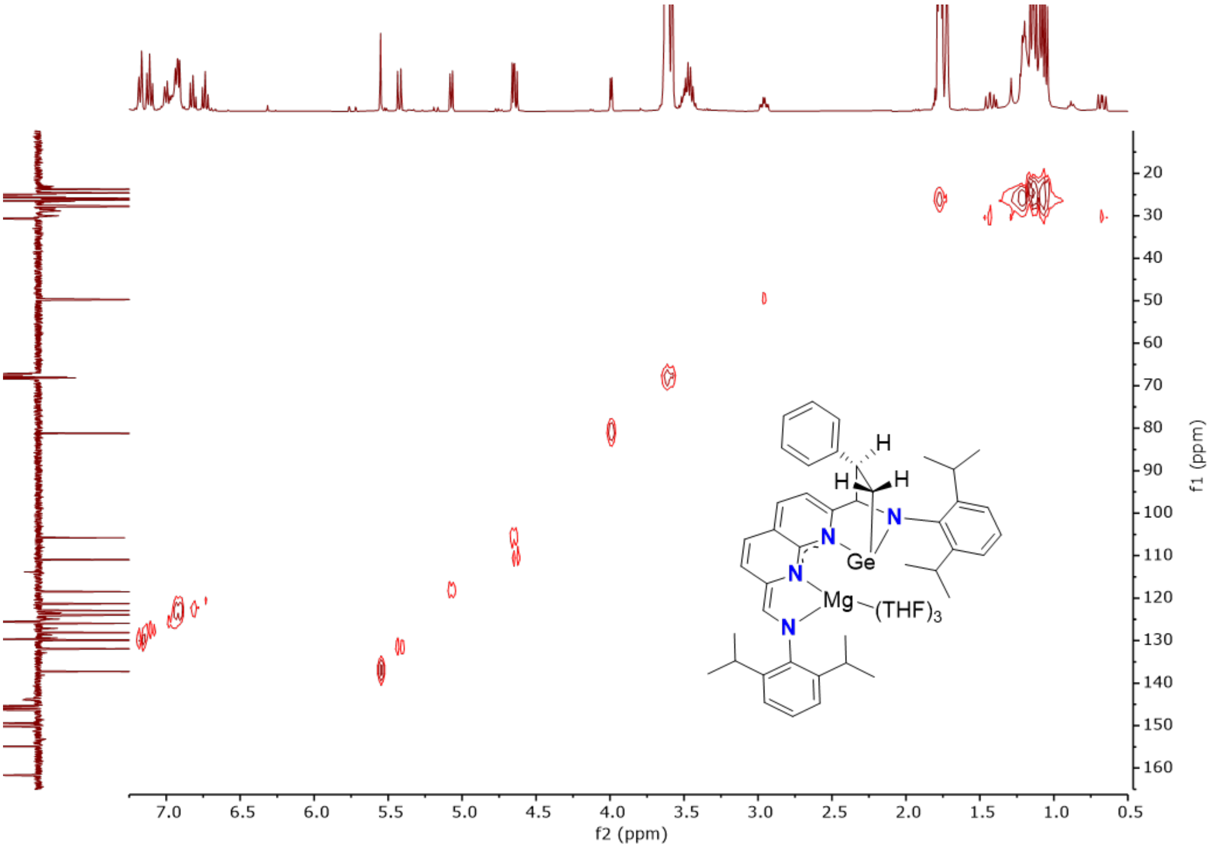

Figure S51: ^{1}H-^{13}C ASAP HMQC-NMR spectrum of **7** in THF-d_8 at 298 K.

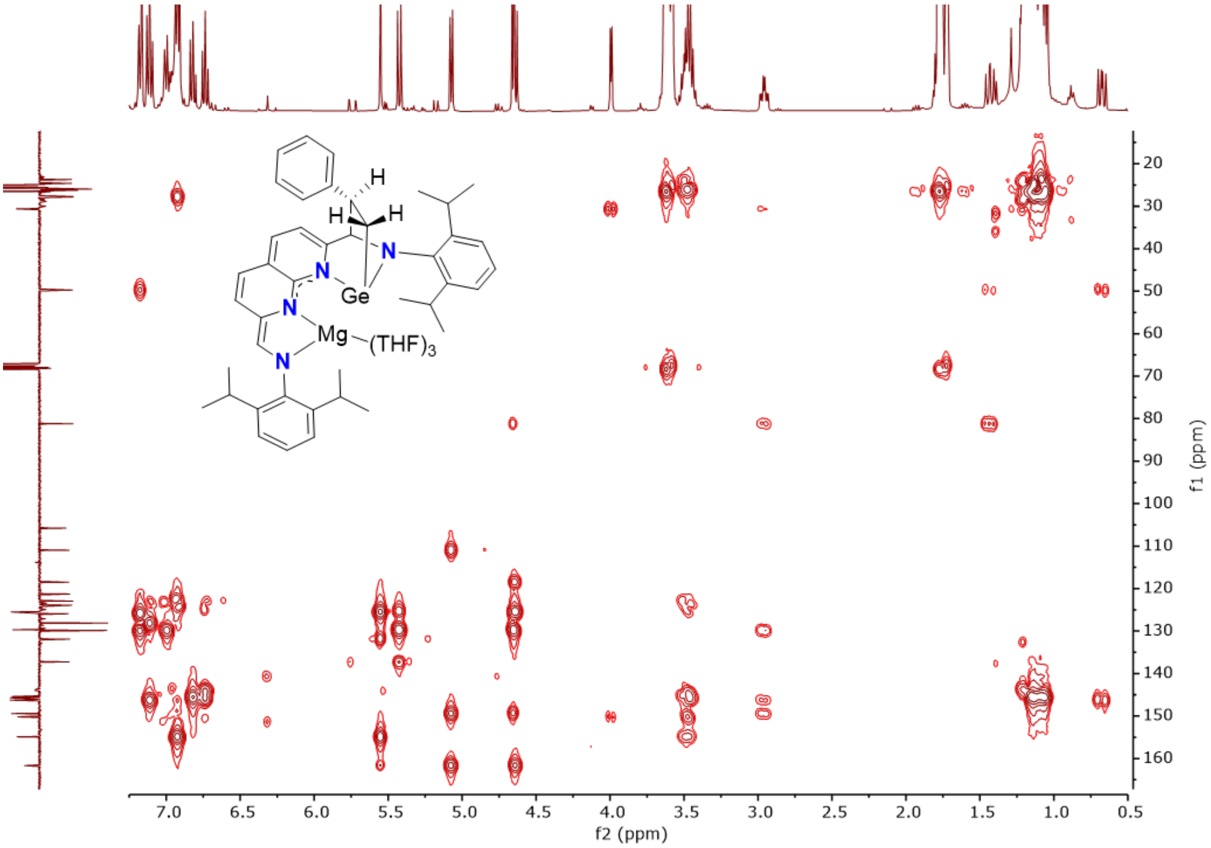

Figure S52: ^{1}H-^{13}C HMBC-NMR spectrum of **7** in THF-d_8 at 298 K.

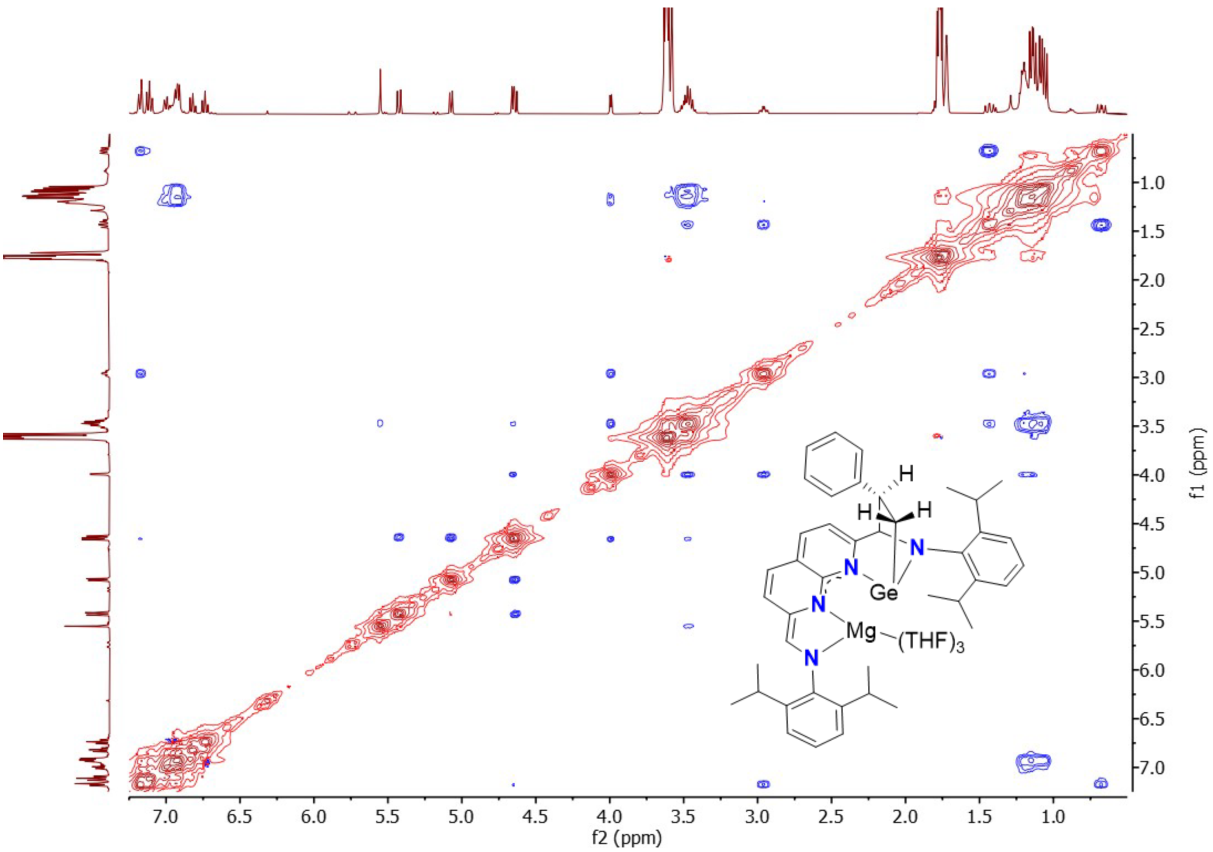

Figure S53: 2D-NOESY spectrum of **7** in THF-d_8 at 298 K.

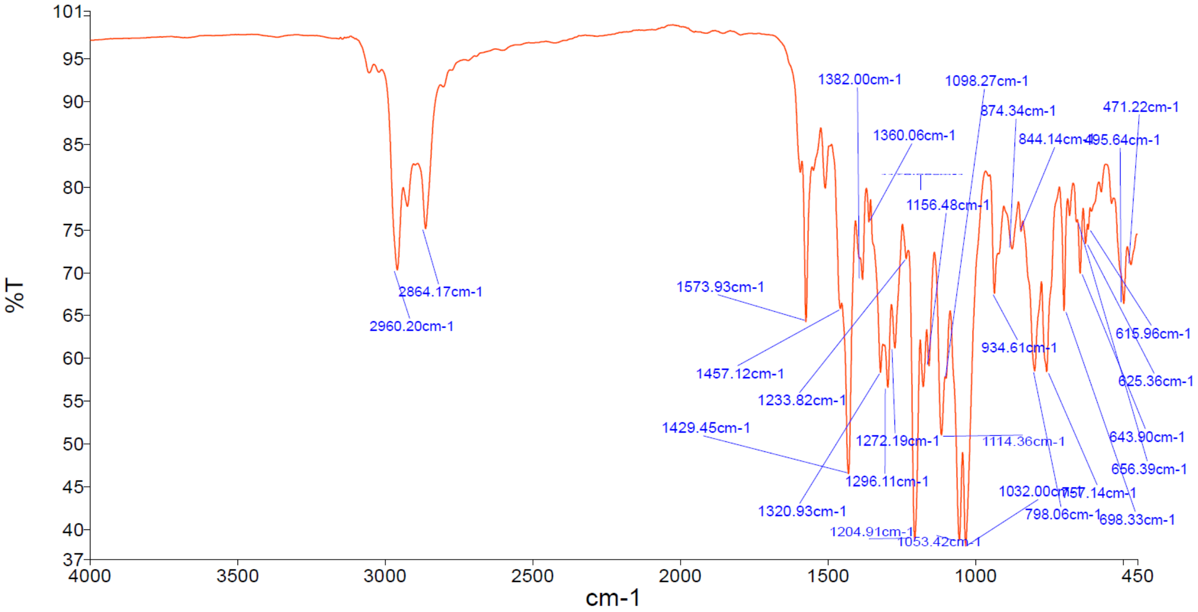

Figure S54: ATR-IR spectrum of **7** measured as a film under N_2 flow at 298 K.

1.10 Synthesis of ^{dipp}**NBA***GeMg·2-methylbut-1-en-3-yne (**8**):

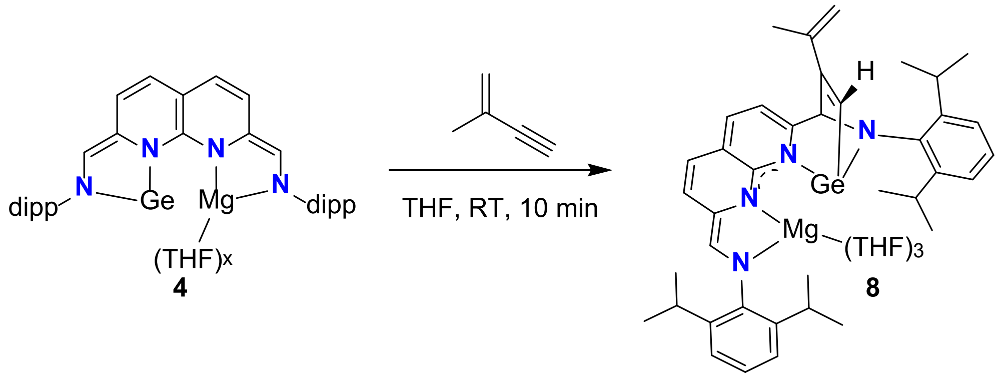

2-Methylbut-1-en-3-yne (0.9 µL, 8.7 µmol, 1.0 equiv) was added by microsyringe to a stirring solution of **4** (8.7 µmol, 1.0 equiv, from stock-solution, see Section 1.6) in THF (1 mL). The mixture was stirred for 10 min at ambient temperature and dried under vacuum to a green film. The film was washed with pentane (2 x 1 mL) and dried in vacuo to a green solid (4.4 mg, 5.0 µmol, 57%).

^{1}H-NMR (400 MHz, THF-d_8, 298 K): δ 7.15 (s 1H), 6.95-6.87 (m, 4H), 6.82 (dd, $^{3}J_{H,H}$ = 7.6 Hz, $^{3}J_{H,H}$ = 7.4 Hz, 1H), 6.99 (t, $^{3}J_{H,H}$ = 7.4 Hz, 1H), 6.94-6.91 (m, 4 H), 6.82 (dd, $^{3}J_{H,H}$ = 7.7 Hz, $^{3}J_{H,H}$ = 7.6 Hz, 1H), 6.69 (dd, $^{3}J_{H,H}$ = 7.6 Hz, $^{3}J_{H,H}$ = 7.5 Hz, 1H), 5.56 (s, 1H), 5.43 (d, $^{3}J_{H,H}$ = 8.9 Hz, 1H), 5.41 (d, $^{3}J_{H,H}$ = 6.4 Hz, 1H), 5.17 (d, $^{3}J_{H,H}$ = 6.4 Hz, 1H), 5.02 (s, 1H), 4.94 (s, 1H), 4.76 (s, 1H), 4.71 (d, $^{3}J_{H,H}$ = 8.9 Hz, 1H), 3.46 (sept*, 4H), 1.87 (s, 1H), 1.22-1.05 (m, 24H).

^{13}C{^{1}H}-NMR (100 MHz, THF-d_8, 298 K): δ 162.2, 160.1, 154.8, 152.3, 150.5, 150.1, 145.7, 145.5, 141.6, 137.6, 131.4, 128.8, 125.1, 123.8, 123.1, 122.9, 121.5, 118.2, 110.8, 110.3, 103.5, 79.1, 27.8, 27.7, 26.2, 24.5, 23.8, 23.1, 21.8.

*Overlapping resonances.

ATR-IR (cm^{-1}): 2960 (s), 2925 (s), 2855 (m), 1458 (m), 1057 (w).

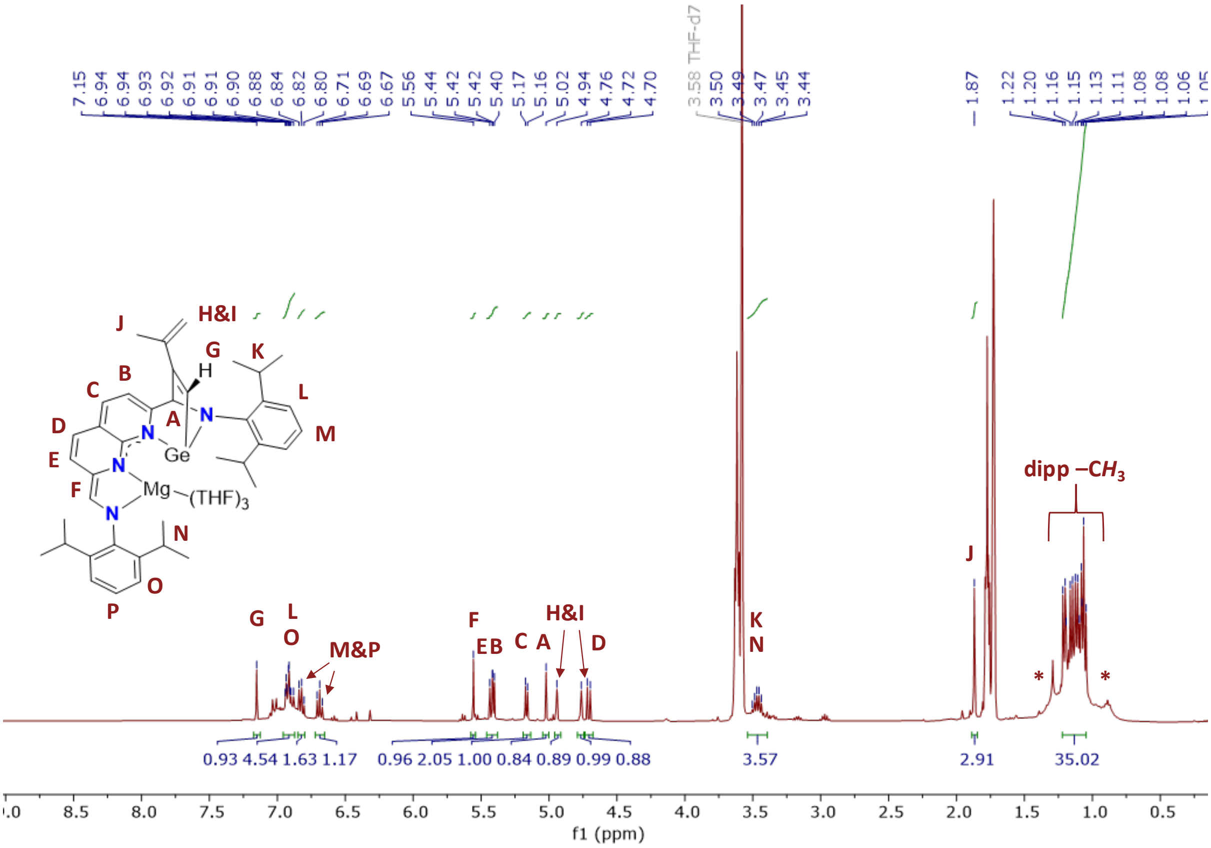

Figure S55: ^{1}H-NMR spectrum of **8** in THF-d_8 at 298 K. Resonances marked with * are attributed to adventitious pentane.

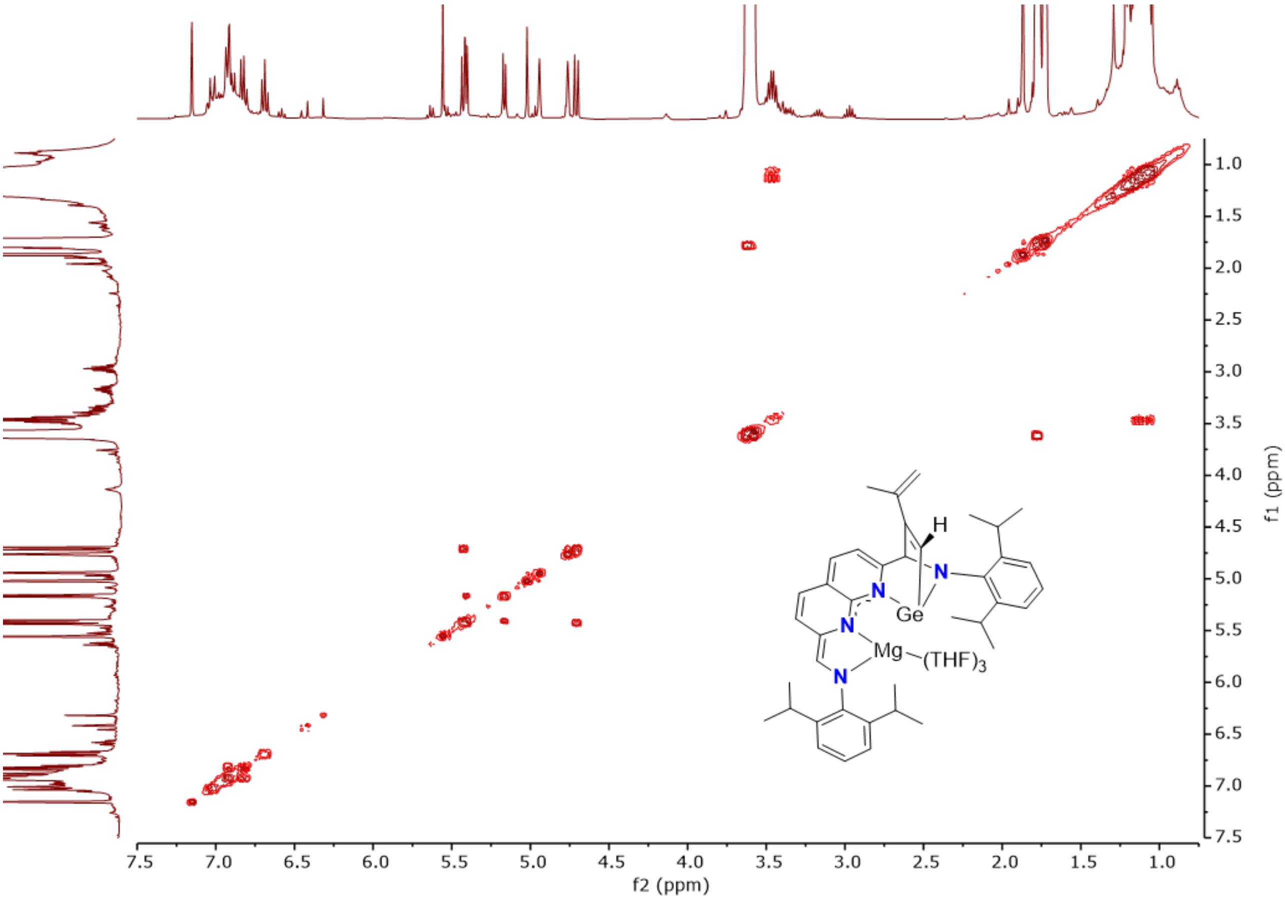

Figure S56: ^{1}H COSY-NMR spectrum of **8** in THF-d_8 at 298 K.

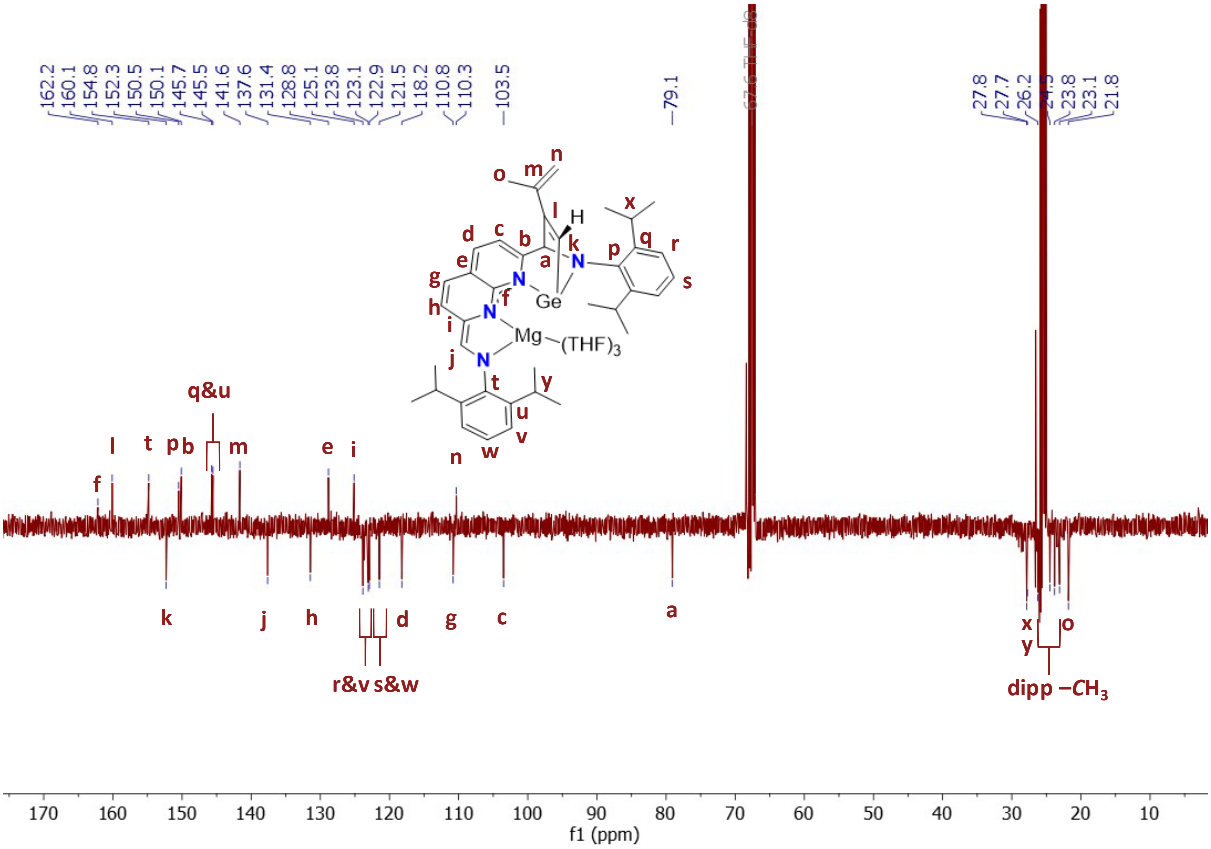

Figure S57: $^{13}C\{^1H\}$-NMR (APT) spectrum of **8** in THF-d_8 at 298 K.

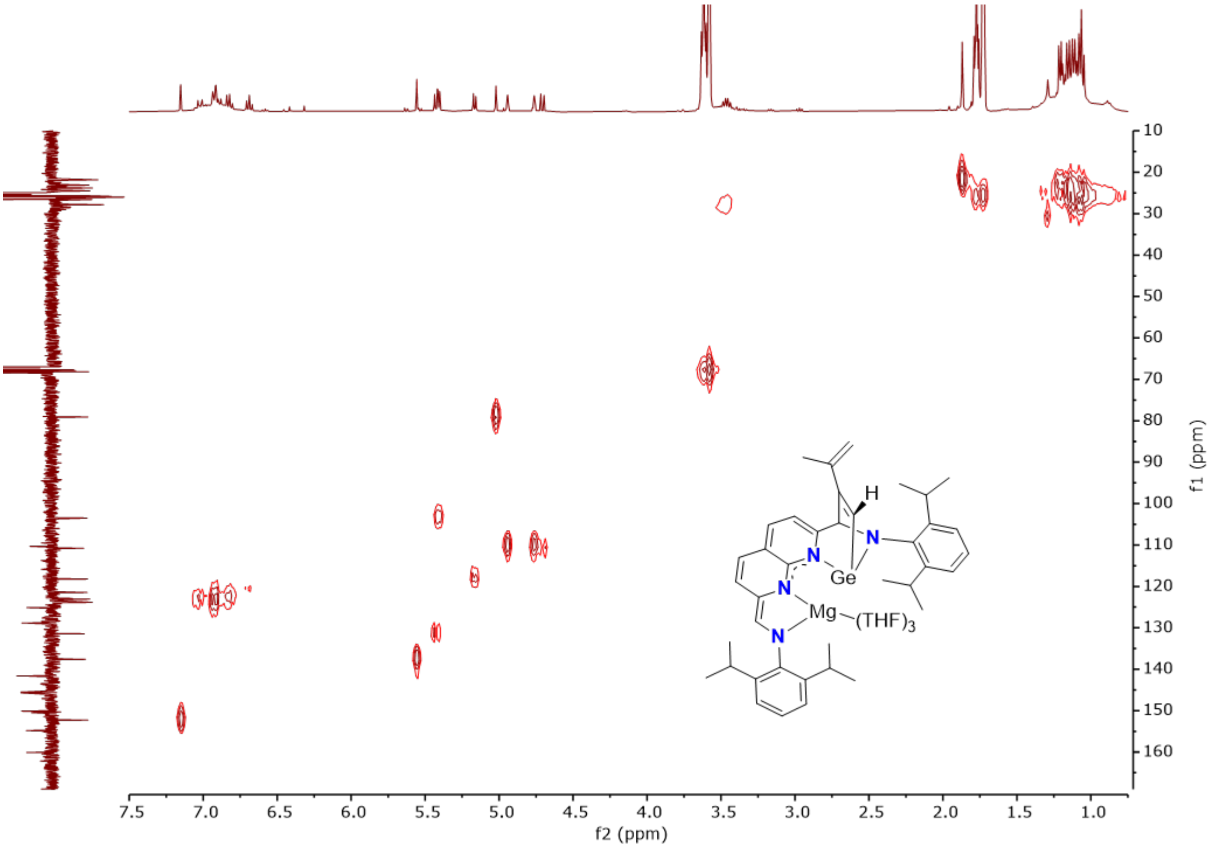

Figure S58: ^1H-^{13}C ASAP HMQC-NMR spectrum of **8** in THF-d_8 at 298 K.

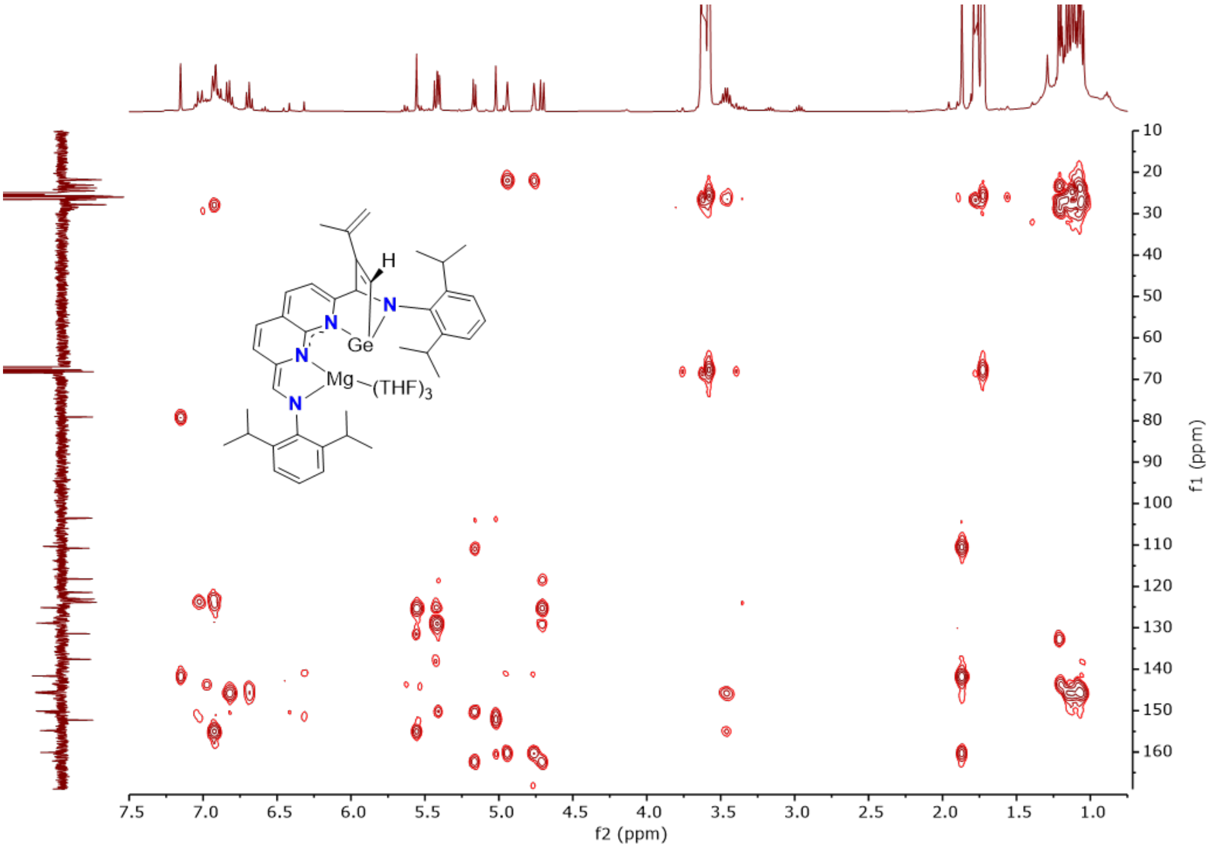

Figure S59: ^{1}H-^{13}C HMBC-NMR spectrum of **8** in THF-d_8 at 298 K.

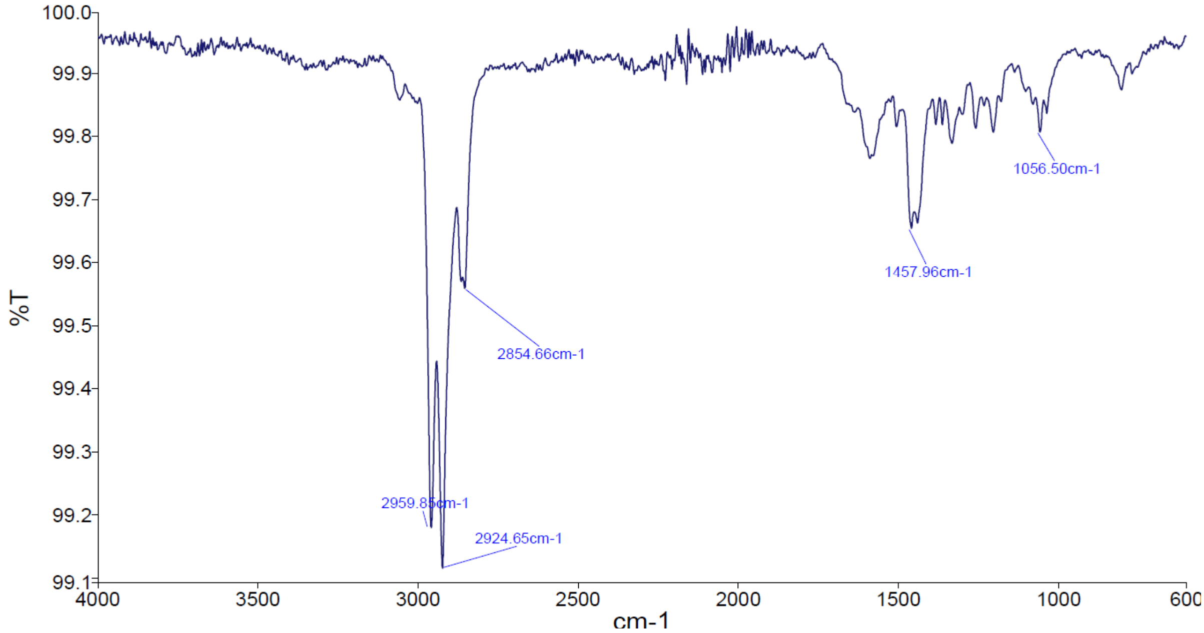

Figure S60: ATR-IR spectrum of **8** measured as a film under N_2 flow at 298 K.

2. Computational Methods:

2.1 General Considerations:

Calculations were performed using the Gaussian 16 rev. C.02 software.[3] The Becke 1988 exchange functional (B3LYP) was used.[4,5] The 6-31G* basis set was used for the geometry optimisations.[6–10] For other calculations the 6-311G** basis set was used.[8,11,12] Starting geometries for the optimisations were obtained from the coordinates of the crystal structures if possible, or by modification of the optimised geometry of the most similar complex. Additionally, Grimme's DFT-D3 scheme for atom-pairwise dispersion correction was used for all atoms in every calculation.[13,14] The absence of imaginary frequencies was checked to confirm that the optimised structures correspond to real local minima. NBO[15] calculations were performed using the NBO 7.0 software.[16] Natural population analysis,[17,18] and computation of Wiberg bond indices[19] were also performed using the NBO 7.0 software. Geometry optimisation with the Ge–Zn distance frozen was performed by using the opt=modredundant keyword and freezing the desired coordinate. Input and output files can be downloaded free of charge from the Yoda data repository DOI: https://doi.org/10.24416/UU01-FJ03BR

2.2 Example Input File for Geometry Optimisation:

```
#p
scf=(maxcycle=300)
opt
freq=noraman
B3LYP/6-31G**
EmpiricalDispersion=GD3BJ
nosym
int=ultrafine

"Title"

0 1
[Cartesian Coordinates here]
```

2.3 Example Input File for NBO Calculations:

```
#p
B3LYP/6-311G**
pop=NBO7Read
density=current
geom=check
guess=read
EmpiricalDispersion=GD3BJ
nosym
int=ultrafine

"Title"
```

0 1

$nbo plot archive file=filename nlmo bndidx $end

2.4 Example Input File for SP Calculations:

```
#p
scf=(maxcycle=300)
SP
B3LYP/6-311G**
EmpiricalDispersion=GD3BJ
nosym
int=ultrafine

“Title”

0 1
[Cartesian Coordinates here]
```

2.5 Electronic Structure of 2:

The geometry-optimised structure in the gas phase of **2** revealed dearomatisation of the naphthyridine backbone, as evidenced by the alternating single and double C–C bond lengths found on the side where Ge was introduced. This contrasts with the other ring of the bicyclic naphthyridine motif, where uniform C–C bond lengths are observed throughout, consistent with the retention of aromatic character. Additionally, bond lengths in line with a $C_{methine}$–C_{napy} double bond and a $C_{methine}$–N single bond were calculated for the Ge-containing side of the system, whereas for the free binding pocket, bond lengths were in line with a $C_{methine}$–C_{napy} single bond and a $C_{methine}$–N double bond. Combined, these observations are in agreement with Ge being bound in a two-electron reduced pocket, forming a NHGe structure, whilst the free binding pocket retains its imine character, consistent with our spectroscopic observations.

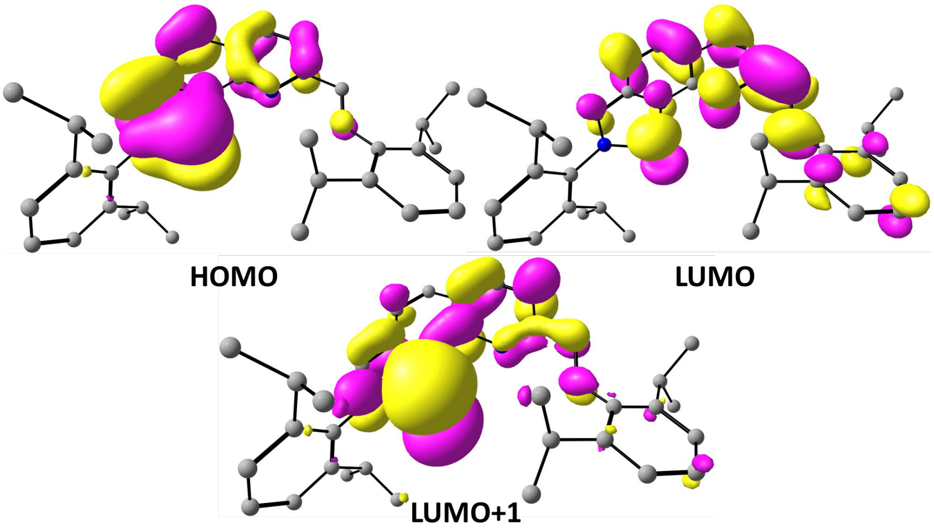

Figure S61: Frontier orbitals of gas-phase optimised **2**. Hydrogen atoms are omitted for clarity.

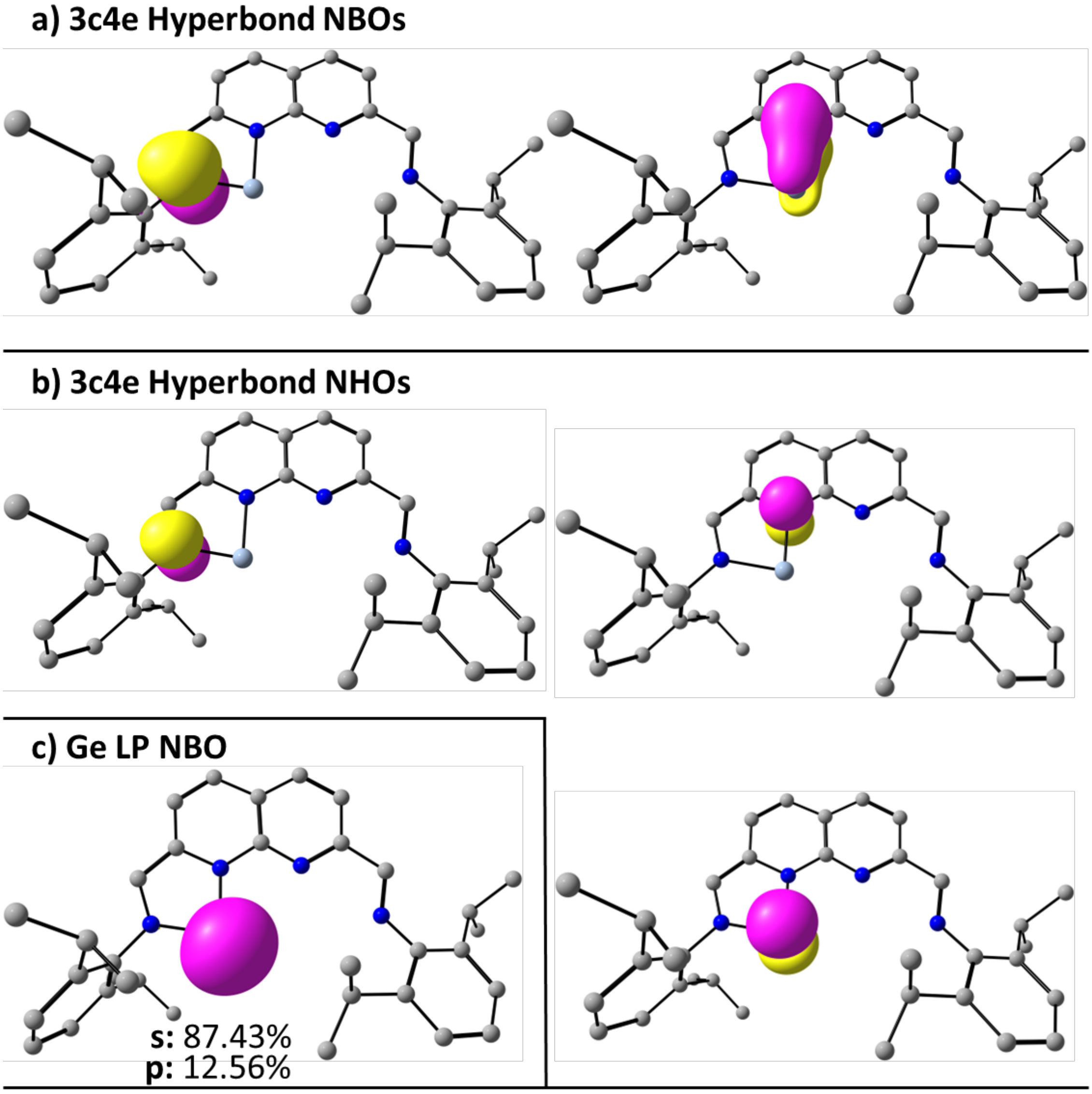

Figure S62: The constituent 3c4e NBOs (**a**) and NHOs (**b**), and the mostly s-character NBO of the germylene lone pair of **2**. Hydrogen atoms are omitted for clarity.

Table S1: Comparison of bond lengths in the solid-state of **3** with the calculated bond metrics found for **3a**, **3b**, and the free germylene **2**. Bond lengths are in Å.

Bond	3 (XRD)	3a	3b	2
**Ge1–Zn1**	3.1110(9)	2.861	3.111	-
**Ge1–N1**	1.920(4)	1.942	1.959	1.913
**Ge1–N3**	1.849(4)	1.896	1.898	1.902
**Zn1–Cl1**	2.2142(17)	2.294	2.286	-
**Zn1–Cl2**	2.2207(16)	2.221	2.218	-
**Zn1–N2**	2.139(4)	2.107	2.116	-
**Zn1–N4**	2.064(4)	2.061	2.022	-
**N1–C2**	1.409(7)	1.412	1.415	1.400
**N1–C6**	1.382(6)	1.365	1.370	1.384
**N2–C6**	1.330(6)	1.339	1.342	1.324
**N2–C9**	1.380(6)	1.365	1.369	1.342
**N3–C1**	1.378(7)	1.368	1.363	1.374
**N4–C10**	1.283(6)	1.289	1.291	1.273
**C1–C2**	1.360(8)	1.372	1.371	1.375
**C2–C3**	1.423(8)	1.419	1.416	1.432
**C3–C4**	1.348(8)	1.362	1.361	1.361
**C4–C5**	1.434(7)	1.436	1.433	1.443
**C5–C6**	1.443(7)	1.439	1.444	1.422
**C5–C7**	1.385(7)	1.396	1.396	1.402
**C7–C8**	1.373(8)	1.394	1.391	1.393
**C8–C9**	1.383(7)	1.388	1.388	1.403
**C9–C10**	1.452(7)	1.450	1.448	1.478

2.6 XYZ Coordinates of **2**:

C	-4.40886	0.69702	-0.84060
C	-5.54040	-0.11044	-0.75118
C	-3.13478	0.11791	-0.92518
C	-5.37494	-1.50302	-0.71777
C	-3.09491	-1.30308	-0.91365
N	-4.16455	-2.07561	-0.80050
H	-6.53356	0.32325	-0.69576
H	-4.50368	1.77920	-0.85196
C	-1.88801	0.83675	-1.03668
C	-0.71534	0.15491	-1.14168
C	-0.69069	-1.27658	-1.15344
N	-1.89529	-1.98061	-1.04029
H	-1.90509	1.92183	-1.03664
H	0.22926	0.68236	-1.22609
C	-6.51467	-2.44084	-0.64138
C	0.39211	-2.11548	-1.27854
N	0.07543	-3.45218	-1.27277
H	1.42132	-1.79491	-1.38068
N	-6.27831	-3.64238	-0.29395
C	-7.13387	-4.74138	-0.33584
C	1.09962	-4.43907	-1.43032

C	-6.51151	-5.91483	-0.84989
C	-7.26871	-7.07833	-0.95876
C	-5.06142	-5.83403	-1.30333
C	-4.28837	-7.15208	-1.19030
H	-4.41262	-7.60538	-0.20182
H	-3.22092	-6.95557	-1.33926
H	-4.59922	-7.88673	-1.94190
C	-4.96948	-5.26423	-2.73118
H	-4.58286	-5.10497	-0.64261
H	-3.92295	-5.14283	-3.03275
H	-5.44705	-4.28350	-2.79210
H	-5.45816	-5.93220	-3.44972
C	-8.59603	-7.10883	-0.52930
C	-8.46355	-4.77123	0.14333
H	-6.81580	-7.97475	-1.36922
C	-9.17230	-5.97313	0.02998
H	-9.17462	-8.02383	-0.61462
H	-10.19621	-6.01866	0.38830
C	-9.12047	-3.56835	0.80469
C	-10.16576	-2.92495	-0.12304
H	-9.72664	-2.63348	-1.08230
H	-10.60445	-2.03426	0.33995
H	-10.97718	-3.62874	-0.33710
C	-9.74476	-3.92262	2.16462
H	-8.34229	-2.82569	1.00143
H	-10.12291	-3.01951	2.65517
H	-9.00810	-4.38906	2.82472
H	-10.58628	-4.61430	2.05845
C	1.68982	-5.00631	-0.28479
C	1.45605	-4.84987	-2.72963
C	2.44860	-5.82581	-2.86304
C	2.67648	-5.98106	-0.46726
C	3.06028	-6.38279	-1.74334
H	3.82900	-7.13999	-1.86543
H	3.14342	-6.43617	0.40026
H	2.73727	-6.16176	-3.85378
C	0.71958	-4.31891	-3.95036
C	1.66203	-3.91238	-5.09135
H	1.09103	-3.46722	-5.91243
H	2.20418	-4.77149	-5.49971
H	2.40099	-3.17997	-4.75255
C	-0.31436	-5.35832	-4.42206
H	0.16651	-3.42663	-3.64618
H	0.18091	-6.27389	-4.76317
H	-0.91257	-4.96431	-5.25045
H	-0.99260	-5.63131	-3.60741
C	1.22042	-4.62992	1.11225

C	0.29403	-5.72671	1.67041
H	-0.09156	-5.44272	2.65533
H	0.83376	-6.67413	1.77608
H	-0.55962	-5.90225	1.00772
C	2.38249	-4.34527	2.07452
H	0.63082	-3.71310	1.02967
H	1.99777	-3.99744	3.03854
H	3.04858	-3.57503	1.67419
H	2.98257	-5.24061	2.26696
Ge	-1.76767	-3.88793	-1.10108
H	-7.49456	-2.06339	-0.96462

2.7 XYZ Coordinates of **3a**:

Ge	3.25306	2.94869	11.03183
Zn	2.49457	3.54883	13.72489
Cl	2.56270	1.27569	13.42622
Cl	4.25271	4.89407	13.90569
N	1.36021	3.01286	10.60092
N	0.71311	3.88768	12.65239
N	3.32883	2.11027	9.33250
N	1.34049	4.31951	15.24873
C	2.13462	1.96454	8.68114
C	1.03926	2.45908	9.34211
C	-0.30424	2.47801	8.88724
C	-1.29125	3.01890	9.65439
C	-0.98607	3.53281	10.95956
C	0.37595	3.48557	11.42019
C	-1.94289	4.07278	11.82063
C	-1.57712	4.49958	13.09588
C	-0.25273	4.36564	13.49050
C	0.16707	4.66741	14.84563
C	4.55276	1.69778	8.71666
C	5.16621	0.50555	9.14479
C	6.35388	0.11800	8.51506
C	6.91525	0.89159	7.50404
C	6.31015	2.08577	7.12035
C	5.12487	2.51692	7.72306
C	4.61347	-0.29134	10.31588
C	4.53774	-1.79862	10.03720
C	5.45021	0.00131	11.57582
C	4.53748	3.88224	7.39425
C	4.50516	4.17985	5.88912
C	5.30898	4.97328	8.16182
C	1.81788	4.72413	16.52577
C	2.28876	3.72154	17.39669

C	2.74799	4.10821	18.65654
C	2.76641	5.44801	19.03426
C	2.35410	6.42592	18.13586
C	1.88441	6.09460	16.86045
C	2.32125	2.25938	16.98455
C	1.57204	7.21450	15.87556
C	0.36566	8.05297	16.32759
C	2.81289	8.09247	15.63903
H	2.09925	1.49673	7.70454
H	-0.52128	2.05970	7.90998
H	-2.31918	3.04984	9.31069
H	-2.97493	4.13814	11.49123
H	-2.30926	4.89864	13.78882
H	-0.53344	5.18184	15.50531
H	6.84730	-0.79641	8.82761
H	7.83597	0.57262	7.02486
H	6.77372	2.69847	6.35430
H	3.59404	0.05236	10.51150
H	3.94881	-2.00801	9.13886
H	4.06762	-2.31105	10.88210
H	5.53060	-2.23908	9.89859
H	6.46995	-0.38215	11.45878
H	4.99646	-0.45977	12.45676
H	5.52186	1.07732	11.76423
H	3.50558	3.90317	7.75327
H	5.51186	4.26360	5.46743
H	3.99564	5.13069	5.70358
H	3.97620	3.39502	5.33969
H	5.29196	4.79032	9.24120
H	4.87195	5.96011	7.97605
H	6.35761	4.99942	7.84705
H	3.09967	3.34876	19.34737
H	3.12282	5.73118	20.01989
H	2.41505	7.47192	18.41885
H	1.32710	6.77183	14.90756
H	-0.53182	7.43720	16.45271
H	0.14381	8.83553	15.59471
H	0.56178	8.54096	17.28785
H	3.11913	8.61187	16.55277
H	2.59751	8.85181	14.87981
H	3.64671	7.47953	15.28920
C	1.51863	1.36886	17.94490
C	3.77443	1.77564	16.84308
H	1.85452	2.16682	16.00194
H	1.95020	1.36976	18.95143
H	1.51467	0.33548	17.58477
H	0.48088	1.70772	18.02561

H	4.33191	2.40588	16.14455
H	4.29215	1.80024	17.80829
H	3.79562	0.75035	16.46342

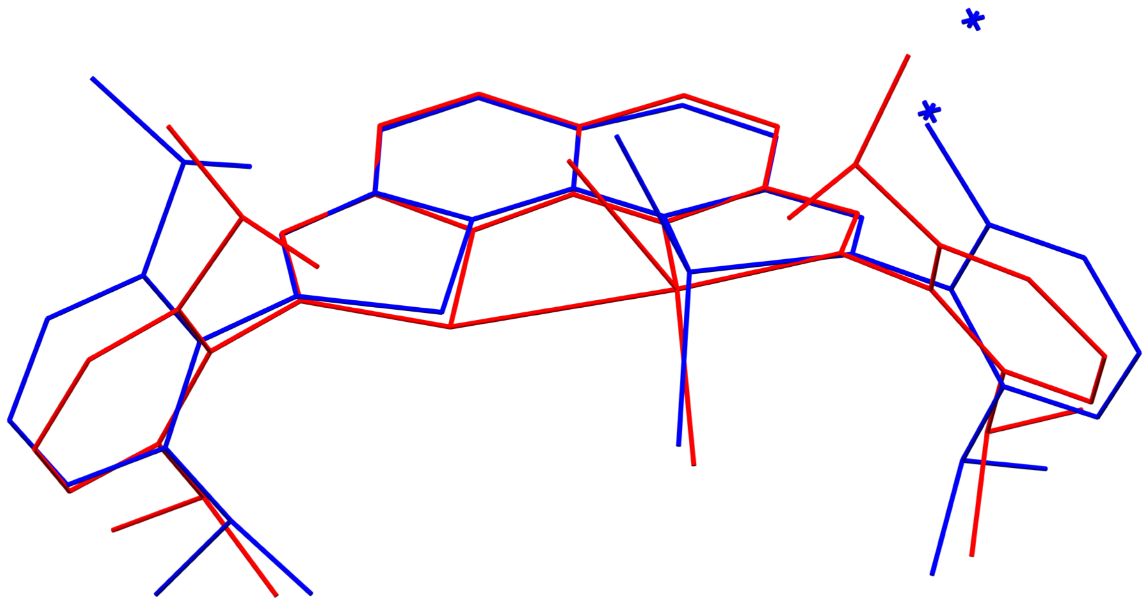

Figure S63: Overlay of gas-phase optimised structure **3a** (red) with the solid-state structure of **3**.

2.8 XYZ Coordinates of **3a** Without Empirical Dispersion:

Ge	3.31849	2.84399	11.02875
Zn	2.69109	3.62854	13.71539
Cl	3.08583	1.37717	13.72912
Cl	4.19794	5.25513	13.47821
N	1.40395	2.79270	10.69894
N	0.80365	3.68985	12.76031
N	3.36303	2.04716	9.30220
N	1.53938	4.36671	15.30565
C	2.14563	1.83564	8.71574
C	1.05608	2.23978	9.44649
C	-0.30853	2.16716	9.05973
C	-1.29036	2.63265	9.88071
C	-0.95321	3.17928	11.16596
C	0.43110	3.22755	11.55855
C	-1.89958	3.67089	12.06739
C	-1.49806	4.16023	13.30875
C	-0.14664	4.13692	13.63074
C	0.31737	4.55435	14.94432
C	4.58074	1.70809	8.61998
C	5.16363	0.44133	8.83871
C	6.35158	0.14066	8.16133
C	6.94545	1.06206	7.30447
C	6.36509	2.31333	7.11780

C	5.17751	2.66537	7.76964
C	4.57361	-0.55863	9.82870
C	4.39754	-1.96275	9.22464
C	5.42589	-0.60390	11.11328
C	4.59989	4.06858	7.60189
C	4.47583	4.49007	6.12701
C	5.42642	5.09462	8.40490
C	1.98583	4.87050	16.57108
C	2.41191	3.93058	17.53747
C	2.83293	4.41262	18.77912
C	2.85294	5.77809	19.05272
C	2.46385	6.68543	18.07480
C	2.02832	6.26318	16.81154
C	2.34190	2.43453	17.25647
C	1.68118	7.31705	15.76057
C	0.41934	8.11284	16.14790
C	2.87009	8.26023	15.49441
H	2.08074	1.38400	7.73226
H	-0.54624	1.74058	8.09044
H	-2.33465	2.59065	9.59038
H	-2.94996	3.65833	11.79277
H	-2.21961	4.53422	14.02686
H	-0.40646	5.02302	15.61443
H	6.82206	-0.82561	8.31542
H	7.86768	0.80903	6.78930
H	6.84705	3.03233	6.46248
H	3.58006	-0.20331	10.11607
H	3.78523	-1.93501	8.31745
H	3.90571	-2.62321	9.94628
H	5.35780	-2.42106	8.96518
H	6.43729	-0.96953	10.90312
H	4.96890	-1.26606	11.85540
H	5.51372	0.38895	11.56462
H	3.58995	4.06885	8.02145
H	5.45369	4.58662	5.64370
H	3.98015	5.46380	6.05395
H	3.88949	3.76644	5.55173
H	5.46107	4.83858	9.46882
H	4.99041	6.09534	8.31333
H	6.45833	5.14113	8.03999
H	3.14910	3.70986	19.54266
H	3.18361	6.13379	20.02420
H	2.50870	7.74924	18.28702
H	1.47933	6.81051	14.81359
H	-0.44869	7.45970	16.29200
H	0.17018	8.84018	15.36778
H	0.56850	8.66559	17.08167

H	3.13349	8.84402	16.38287
H	2.61568	8.96717	14.69729
H	3.74610	7.69046	15.17646
C	0.98904	1.85457	17.71710
C	3.51102	1.64337	17.86338
H	2.40132	2.29558	16.17352
H	0.86848	1.95574	18.80173
H	0.92232	0.79136	17.46384
H	0.14693	2.36689	17.24006
H	4.47425	2.07351	17.57432
H	3.46618	1.60865	18.95769
H	3.48234	0.61102	17.50196

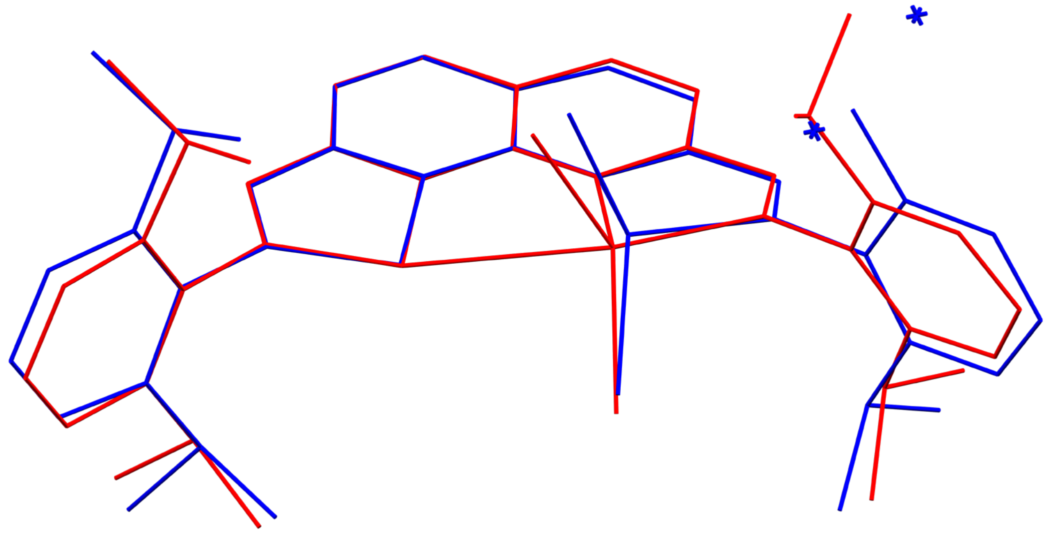

Figure S64: Overlay of gas-phase optimised structure **3a** without empirical dispersion (red) with the solid-state structure of **3** (blue).

2.9 XYZ Coordinates of **3b**:

Ge	3.42583	3.09325	10.90188
Zn	2.62736	3.69701	13.84743
Cl	2.97497	1.48724	13.37802
Cl	4.26648	5.19013	13.79419
N	1.48442	3.02383	10.64874
N	0.86214	3.94806	12.70837
N	3.40726	2.09356	9.28840
N	1.45004	4.39484	15.33580
C	2.17660	1.85515	8.75248
C	1.11305	2.36425	9.45336
C	-0.24490	2.31142	9.05617
C	-1.21088	2.89900	9.81424
C	-0.86309	3.50638	11.06423
C	0.51643	3.50457	11.48991

C	-1.81010	4.09236	11.90588
C	-1.42923	4.58584	13.14936
C	-0.10186	4.45926	13.53596
C	0.29586	4.76670	14.89401
C	4.59833	1.69418	8.60505
C	5.32313	0.58741	9.08577
C	6.48015	0.21192	8.39470
C	6.90518	0.91449	7.27163
C	6.19018	2.02538	6.83134
C	5.03049	2.44223	7.49127
C	4.91553	-0.13166	10.36188
C	4.89597	-1.65886	10.20867
C	5.83858	0.30046	11.51651
C	4.32697	3.72823	7.07998
C	4.12076	3.84250	5.56329
C	5.10078	4.94101	7.63122
C	1.88997	4.74532	16.63991
C	2.29373	3.68693	17.48336
C	2.71065	4.00253	18.77586
C	2.75280	5.32673	19.21002
C	2.40694	6.35552	18.34219
C	1.98035	6.09551	17.03422
C	2.21713	2.25392	16.98521
C	1.72701	7.26070	16.08684
C	0.51187	8.09419	16.52522
C	2.98574	8.13254	15.94227
H	2.08168	1.31564	7.81734
H	-0.48782	1.81012	8.12517
H	-2.25222	2.88959	9.51236
H	-2.84673	4.13219	11.58727
H	-2.15248	5.01690	13.83249
H	-0.41593	5.28460	15.53820
H	7.05715	-0.63706	8.74647
H	7.80433	0.60607	6.74676
H	6.54735	2.58579	5.97358
H	3.89956	0.18015	10.61855
H	4.24575	-1.96755	9.38427
H	4.52548	-2.12127	11.12866
H	5.89551	-2.06337	10.01796
H	6.86702	-0.03023	11.33305
H	5.49435	-0.12281	12.46359
H	5.85489	1.38938	11.62693
H	3.33785	3.73898	7.54402
H	5.07149	3.92216	5.02665
H	3.53858	4.73909	5.32785
H	3.58574	2.97333	5.16836
H	5.20176	4.88671	8.72001

H	4.58561	5.87480	7.38215
H	6.10917	4.98362	7.20614
H	3.01049	3.20781	19.44926
H	3.07750	5.55570	20.22039
H	2.48675	7.38554	18.67465
H	1.52065	6.86188	15.09102
H	-0.39687	7.48535	16.58729
H	0.32914	8.90968	15.81801
H	0.67378	8.53845	17.51282
H	3.26308	8.60220	16.89158
H	2.80752	8.93082	15.21427
H	3.82432	7.52789	15.59007
C	0.78882	1.69829	17.12502
C	3.23659	1.31790	17.64098
H	2.44697	2.26825	15.91586
H	0.49592	1.64882	18.17947
H	0.72902	0.69071	16.70213
H	0.06201	2.32667	16.60244
H	4.24803	1.72986	17.58386
H	3.00125	1.12790	18.69394
H	3.23330	0.35420	17.12416

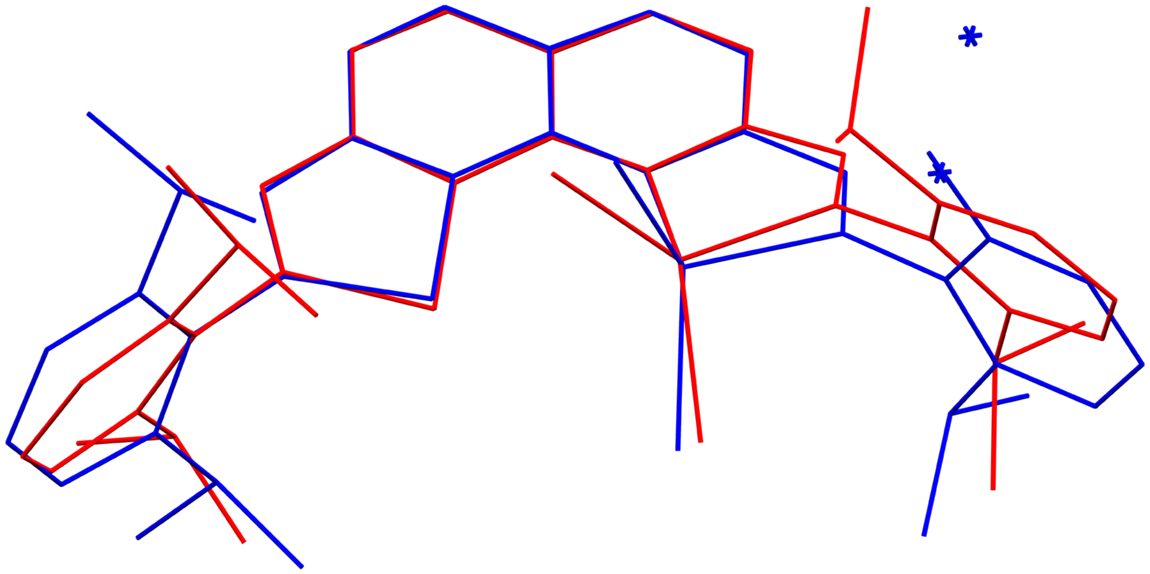

Figure S65: Overlay of gas-phase optimised structure **3b** (red) with the solid-state structure of **3**.

2.10 Electronic Structure of Complexes **4a-4d**:

Geometry optimisations and electronic structure calculations were performed on **4** bearing varying amounts of coordinated THF molecules. The structure of **4** bearing no coordinated THF molecules (**4a**) revealed distorted binding of the GeMg core (Ge–Mg distance = 2.643 Å) with the Mg centre being positioned out of the naphthyridine plane (Figure S65). To

investigate this displacement, we performed NBO calculations, which assigned a Lewis structure consistent with preferential reduction of the germylene centre to a germylone over two-electron reduction of the diimine pocket. 2nd Order perturbation analysis revealed that the displacement of the Mg centre out of the naphthyridine plane is caused by electronic effects. Specifically, this displacement maximises overlap of the donor NBOs on Ge with the acceptor NBO on Mg. The two donor NBOs on Ge are the two lone pairs, one residing in an orbital of s-character (83.9%) similar to **2**, and one residing in an orbital of purely p-character (Figure S66). Both donate into an orbital of mostly s-character (95.4%) on Mg (Figure S66), with the latter donor NBO being energetically the major contributor (154.0 *vs*. 19.6 kcal·mol^{-1}). Compared to the Ge–Mg interactions, the N–Mg interactions are weaker (15.1 and 19.4 kcal·mol^{-1}).

The geometry optimisation of **4** bearing a single coordinated THF molecule (**4b**) resulted in a square planar geometry around the Mg centre with a Ge–Mg distance of 2.570 Å. NBO calculations converged on a Ge^{II}–Mg^{II} core bound to a four-electron reduced **^{dipp}NBA** ligand. 2nd Order perturbation analysis revealed a delocalisation energy of 32.4 kcal·mol^{-1} (Figure S67) from the Ge donor NBO (79.9% s-character) to the acceptor NBO on Mg (99.5% s-character). Comparatively, this delocalisation energy is larger than the delocalisation energies of the two N-donors (16.2 and 20.6 kcal·mol^{-1}) and the combined donation of the two lone pairs on THF (16.7 kcal·mol^{-1}).

In a similar fashion, the geometry optimisation of **4** bound to two THF molecules (**4c**) resulted in a slightly distorted square pyramidal geometry around Mg, with a Ge–Mg distance of 2.661 Å. NBO calculations resulted in a Lewis structure similar to **4b**, with an additional THF ligand. Despite the increased coordinative and electronic saturation of the Mg centre, 2nd order perturbation analysis revealed that the delocalisation energy from the Ge lone pair (81.9% s-character) NBO to the Mg acceptor NBO (99.1% s-character) is still appreciably larger (29.1 kcal·mol^{-1}, Figure S68) than the interactions from the N-donors and O-donors (11.7, 15.8, 11.4 and 9.7 kcal·mol^{-1} respectively).

The geometry-optimised structure of **4** bound to three THF molecules (**4d**) features a slightly distorted octahedral geometry around Mg, with a Ge–Mg distance of 2.896 Å. NBO calculations converged on a Lewis structure similar to **4b** and **4c**. 2nd order perturbation analysis revealed that the delocalisation energy from the Ge lone pair (86.8% s-character) NBO to the Mg acceptor NBO (99.3% s-character) is appreciably larger (20.3 kcal·mol^{-1}, Figure S69) than the interactions from the N-donors and O-donors (11.8, 7.8, 9.4, 8.1 and 8.7 kcal·mol^{-1} respectively).

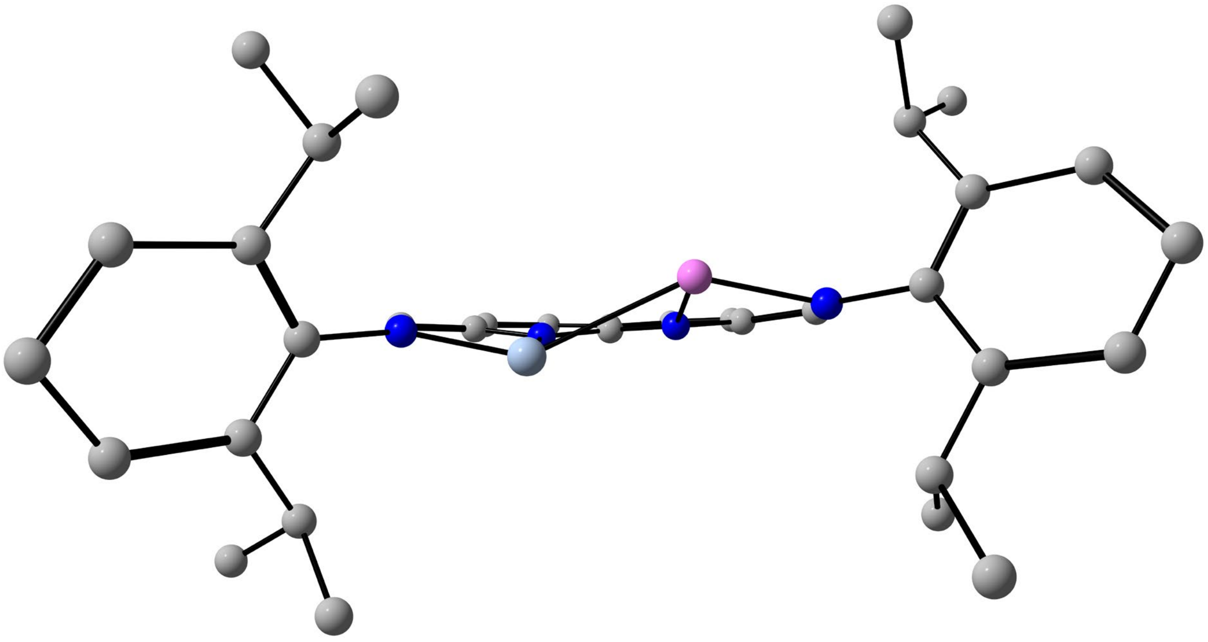

Figure S66: Geometry-optimised gas-phase structure of **4a**, showing the displacement of Mg (pink) out of the naphthyridine plane.

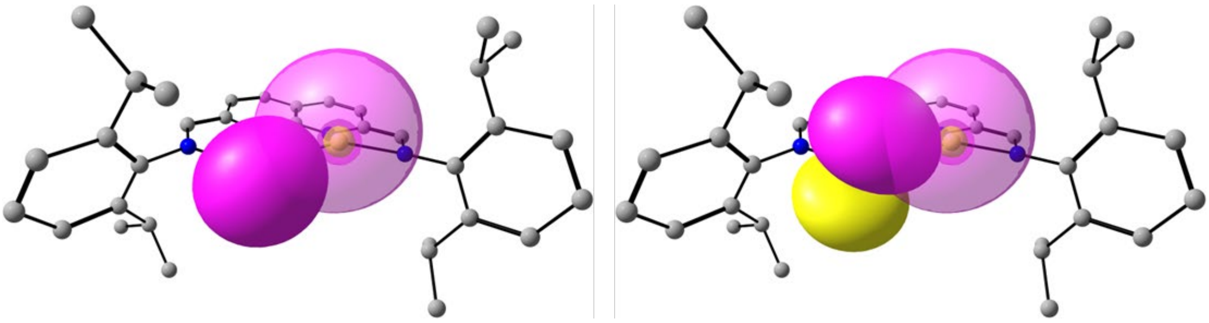

Figure S67: Overlap of the two Ge-based lone pair donor NBOs (filled) with the Mg-based acceptor NBO (translucent) of **4a**.

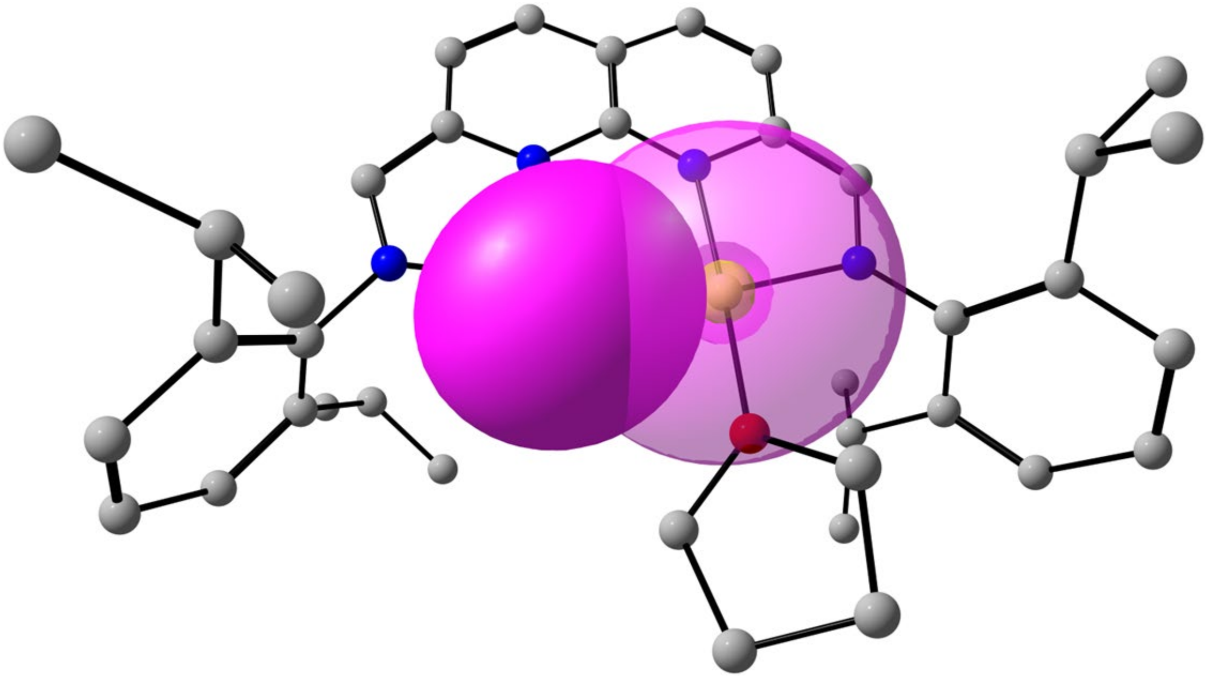

Figure S68: Overlap of the Ge-based lone pair donor NBO (filled) with the Mg-based acceptor NBO (translucent) of **4b**.

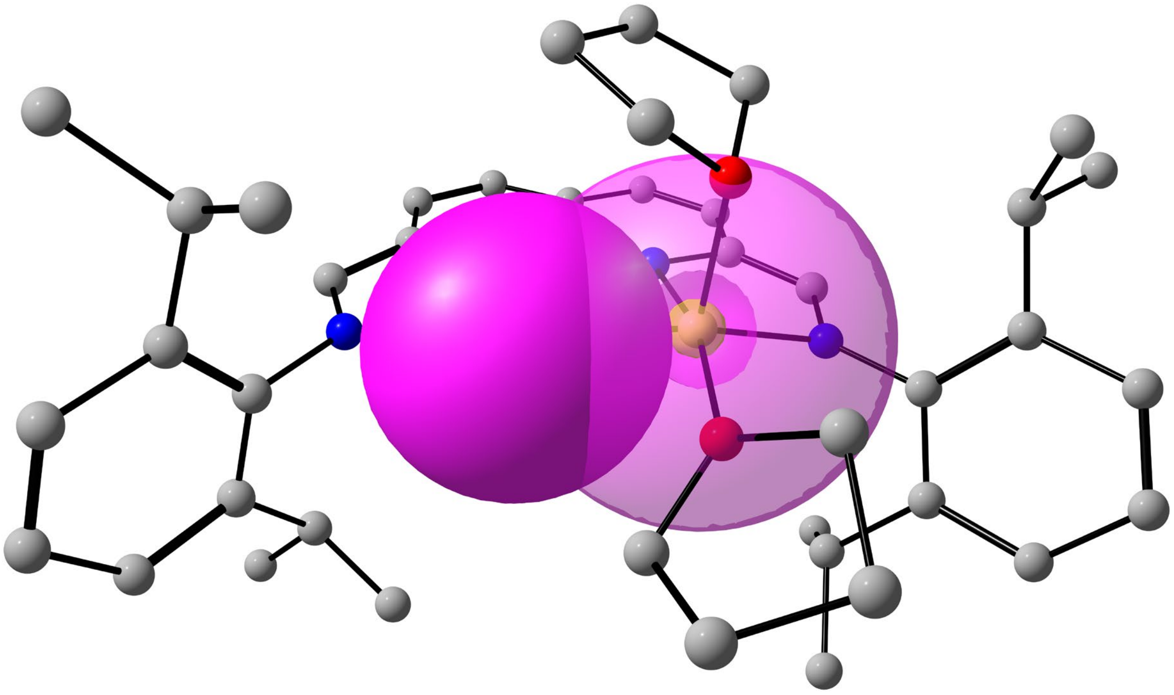

Figure S69: Overlap of the Ge-based lone pair donor NBO (filled) with the Mg-based acceptor NBO (translucent) of **4c**.

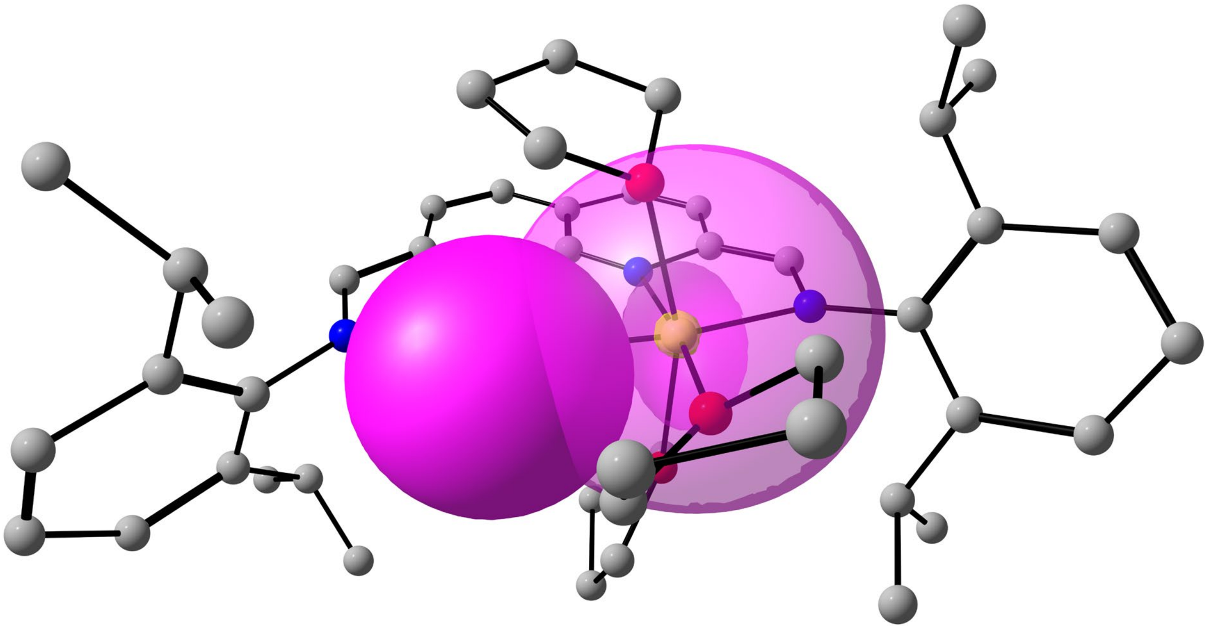

Figure S70: Overlap of the Ge-based lone pair donor NBO (filled) with the Mg-based acceptor NBO (translucent) of **4d**.

2.11 XYZ Coordinates of **4a**:

Ge	3.049823000	3.325952000	10.991280000
Mg	2.459475000	3.232306000	13.565703000
N	1.206432000	2.902176000	10.623644000
N	0.618344000	3.629635000	12.703259000
N	3.286930000	2.239111000	9.430012000
N	1.551460000	4.316297000	15.083208000
C	2.143210000	1.891117000	8.782777000
C	0.959869000	2.230603000	9.427590000

C	-0.382114000	1.992690000	9.052280000
C	-1.409569000	2.379336000	9.887513000
C	-1.143060000	2.960834000	11.161511000
C	0.210992000	3.180263000	11.506433000
C	-2.108165000	3.317454000	12.151143000
C	-1.702232000	3.815225000	13.373708000
C	-0.329939000	3.976235000	13.667528000
C	0.212125000	4.332472000	14.911207000
C	4.555841000	2.052632000	8.793481000
C	5.362777000	0.973589000	9.198519000
C	6.605358000	0.812702000	8.577479000
C	7.032570000	1.697174000	7.591210000
C	6.225281000	2.767930000	7.217752000
C	4.975166000	2.971319000	7.811522000
C	4.923391000	0.039922000	10.315020000
C	5.055324000	-1.441354000	9.934369000
C	5.701271000	0.360671000	11.603350000
C	4.142309000	4.192008000	7.446363000
C	3.917964000	4.312831000	5.931668000
C	4.784966000	5.466093000	8.023200000
C	2.066841000	4.636998000	16.371972000
C	1.961284000	3.727270000	17.444432000
C	2.504403000	4.090617000	18.682835000
C	3.142407000	5.313128000	18.857990000
C	3.260780000	6.193117000	17.783575000
C	2.734945000	5.873253000	16.529759000
C	1.334788000	2.351272000	17.271187000
C	2.827802000	6.837001000	15.355739000
C	1.542419000	7.676830000	15.241143000
C	4.065041000	7.741974000	15.396346000
H	2.197753000	1.416319000	7.810466000
H	-0.586743000	1.503256000	8.106384000
H	-2.441090000	2.201762000	9.599665000
H	-3.162579000	3.178659000	11.935068000
H	-2.434699000	4.068325000	14.134492000
H	-0.441831000	4.553250000	15.753959000
H	7.246053000	-0.011864000	8.873410000
H	8.000093000	1.556825000	7.118648000
H	6.574746000	3.464200000	6.461943000
H	3.867751000	0.240966000	10.515403000
H	4.501758000	-1.664931000	9.017464000
H	4.659969000	-2.074237000	10.735323000
H	6.099487000	-1.729510000	9.774283000
H	6.772620000	0.172052000	11.473688000
H	5.342338000	-0.253975000	12.435513000
H	5.573944000	1.411922000	11.878613000
H	3.160182000	4.088896000	7.913952000

H	4.856984000	4.476449000	5.392952000
H	3.261151000	5.160647000	5.712341000
H	3.454477000	3.407907000	5.526799000
H	4.878700000	5.392576000	9.110840000
H	4.170833000	6.343059000	7.792963000
H	5.783341000	5.630162000	7.603515000
H	2.432546000	3.399532000	19.517682000
H	3.555568000	5.578789000	19.826420000
H	3.769976000	7.140178000	17.925406000
H	2.889176000	6.228361000	14.445551000
H	0.663161000	7.038680000	15.123652000
H	1.594072000	8.347588000	14.376688000
H	1.403882000	8.287726000	16.139991000
H	4.008872000	8.473755000	16.209318000
H	4.146875000	8.305103000	14.461477000
H	4.983870000	7.162178000	15.527862000
C	0.153937000	2.127925000	18.227894000
C	2.398146000	1.250331000	17.423096000
H	0.945946000	2.283050000	16.252125000
H	0.473108000	2.161156000	19.274887000
H	-0.305177000	1.149533000	18.051284000
H	-0.613605000	2.895759000	18.090665000
H	3.217368000	1.404289000	16.712659000
H	2.828162000	1.246413000	18.430093000
H	1.964407000	0.262008000	17.236695000

2.12 XYZ Coordinates of **4b**:

Ge	2.993556000	2.688654000	11.172454000
Mg	2.471864000	3.565613000	13.531657000
N	1.179182000	2.407374000	10.725833000
N	0.590892000	3.163356000	12.811217000
N	3.259870000	2.086951000	9.419260000
N	1.437362000	4.227327000	15.124282000
C	2.122099000	1.750470000	8.732969000
C	0.937849000	1.921267000	9.440809000
C	-0.405413000	1.691943000	9.055705000
C	-1.420391000	1.967772000	9.947755000
C	-1.158933000	2.479303000	11.250000000
C	0.182044000	2.687963000	11.618174000
C	-2.150052000	2.816717000	12.237967000
C	-1.764907000	3.324569000	13.448918000
C	-0.389751000	3.520433000	13.770051000
C	0.085944000	4.052844000	14.954587000
C	4.549633000	1.978133000	8.811367000
C	5.273193000	0.779149000	8.952014000
C	6.544383000	0.707666000	8.372597000

C	7.078429000	1.791811000	7.681413000
C	6.350739000	2.973149000	7.565375000
C	5.075679000	3.090848000	8.128251000
C	4.720661000	-0.379527000	9.767631000
C	4.851294000	-1.729101000	9.048535000
C	5.394260000	-0.413405000	11.151504000
C	4.310366000	4.403654000	8.060586000
C	4.275143000	4.999013000	6.646408000
C	4.892923000	5.403733000	9.075641000
C	1.959751000	4.674996000	16.348873000
C	1.795563000	3.929010000	17.542951000
C	2.398950000	4.389311000	18.719810000
C	3.178763000	5.539522000	18.733154000
C	3.363270000	6.255325000	17.549816000
C	2.766162000	5.848414000	16.355343000
C	1.043751000	2.604497000	17.559323000
C	2.914531000	6.673521000	15.083257000
C	1.629770000	7.477205000	14.814355000
C	4.143235000	7.588752000	15.062041000
H	2.196590000	1.383020000	7.717881000
H	-0.616473000	1.308079000	8.064090000
H	-2.453782000	1.798923000	9.658548000
H	-3.199644000	2.670815000	12.002708000
H	-2.507039000	3.593535000	14.195506000
H	-0.602695000	4.360006000	15.738810000
H	7.124346000	-0.204583000	8.469655000
H	8.066470000	1.717833000	7.236790000
H	6.780339000	3.817707000	7.036092000
H	3.657022000	-0.190274000	9.932619000
H	4.379070000	-1.698860000	8.062113000
H	4.367843000	-2.517180000	9.634532000
H	5.898056000	-2.019656000	8.910638000
H	6.471835000	-0.590285000	11.060950000
H	4.966213000	-1.208658000	11.770914000
H	5.243093000	0.538535000	11.670126000
H	3.279986000	4.201839000	8.363664000
H	5.271859000	5.293199000	6.301228000
H	3.645804000	5.894450000	6.629621000
H	3.868807000	4.282740000	5.926180000
H	4.854460000	4.984580000	10.085936000
H	4.317844000	6.335711000	9.074314000
H	5.935958000	5.642701000	8.840019000
H	2.268811000	3.821688000	19.637079000
H	3.644099000	5.877273000	19.654151000
H	3.972421000	7.152692000	17.566547000
H	3.025963000	5.976242000	14.244357000
H	0.767037000	6.811854000	14.743027000

H	1.710167000	8.039220000	13.877270000
H	1.451679000	8.190046000	15.626684000
H	4.061956000	8.399635000	15.793307000
H	4.244531000	8.053574000	14.076103000
H	5.065963000	7.038448000	15.275419000
C	-0.230940000	2.685352000	18.413964000
C	1.947924000	1.451337000	18.027501000
H	0.739755000	2.380035000	16.534948000
H	0.005761000	2.907508000	19.460380000
H	-0.777215000	1.736415000	18.387578000
H	-0.898020000	3.472824000	18.051049000
H	2.839738000	1.369995000	17.397277000
H	2.282106000	1.589663000	19.061086000
H	1.411345000	0.498061000	17.975988000
O	4.398552000	3.638998000	14.198710000
C	4.846665000	3.131537000	15.488631000
C	6.102999000	3.936592000	15.773451000
C	6.735796000	4.048315000	14.376668000
C	5.525224000	4.180274000	13.446846000
H	5.054680000	2.059857000	15.384727000
H	4.037553000	3.293327000	16.199284000
H	6.754914000	3.446718000	16.500214000
H	5.821793000	4.918707000	16.164210000
H	7.414255000	4.898750000	14.280151000
H	7.297363000	3.139161000	14.140364000
H	5.282546000	5.220637000	13.212943000
H	5.616859000	3.615245000	12.516020000

2.13 XYZ Coordinates of **4c**:

Ge	2.948482000	2.431230000	11.306131000
Mg	2.372178000	3.127441000	13.808831000
N	1.127400000	2.255186000	10.809512000
N	0.514528000	3.039226000	12.884266000
N	3.219107000	1.966906000	9.514582000
N	1.345351000	4.196421000	15.180920000
C	2.084556000	1.708588000	8.786741000
C	0.893950000	1.867462000	9.487454000
C	-0.446878000	1.731575000	9.054296000
C	-1.469457000	2.052277000	9.924596000
C	-1.218080000	2.523181000	11.240165000
C	0.119302000	2.614879000	11.670964000
C	-2.221521000	2.948818000	12.187829000
C	-1.847547000	3.449864000	13.401432000
C	-0.472739000	3.537150000	13.783900000
C	-0.007338000	4.109780000	14.953286000
C	4.510703000	1.933079000	8.902853000

C	5.272821000	0.751858000	8.965210000
C	6.545899000	0.756758000	8.385765000
C	7.045475000	1.899575000	7.767183000
C	6.280802000	3.062511000	7.727535000
C	5.002875000	3.103884000	8.294693000
C	4.754721000	-0.469131000	9.708516000
C	4.976668000	-1.780689000	8.944492000
C	5.385486000	-0.524009000	11.111861000
C	4.198550000	4.395232000	8.314194000
C	4.154359000	5.087997000	6.945304000
C	4.742846000	5.339103000	9.402184000
C	1.872392000	4.772991000	16.344424000
C	1.670816000	4.203919000	17.626930000
C	2.319899000	4.764514000	18.733987000
C	3.170727000	5.855339000	18.600959000
C	3.374697000	6.412115000	17.337668000
C	2.738407000	5.897513000	16.206966000
C	0.837567000	2.944131000	17.815281000
C	2.904086000	6.553123000	14.842211000
C	1.649645000	7.372280000	14.487468000
C	4.164456000	7.413103000	14.700099000
H	2.164836000	1.423791000	7.745988000
H	-0.648848000	1.405379000	8.040585000
H	-2.502107000	1.967062000	9.597282000
H	-3.268328000	2.892683000	11.905435000
H	-2.595106000	3.807851000	14.104459000
H	-0.707999000	4.536254000	15.669963000
H	7.153942000	-0.141457000	8.423635000
H	8.035187000	1.885637000	7.320366000
H	6.684091000	3.953085000	7.256187000
H	3.677630000	-0.334591000	9.840602000
H	4.543830000	-1.731629000	7.941107000
H	4.506837000	-2.612884000	9.478632000
H	6.040237000	-2.019354000	8.840360000
H	6.474171000	-0.627719000	11.050460000
H	4.990498000	-1.369774000	11.685064000
H	5.156085000	0.397901000	11.655403000
H	3.172722000	4.142099000	8.592253000
H	5.142938000	5.439522000	6.631566000
H	3.495074000	5.960758000	6.986104000
H	3.777682000	4.411792000	6.172126000
H	4.701214000	4.852894000	10.381737000
H	4.142683000	6.253586000	9.454589000
H	5.781884000	5.620609000	9.197517000
H	2.169065000	4.322063000	19.715122000
H	3.671071000	6.271839000	19.470018000
H	4.030690000	7.270820000	17.239928000

H	2.968318000	5.751619000	14.098835000
H	0.758848000	6.741738000	14.507020000
H	1.740068000	7.807198000	13.485793000
H	1.513427000	8.189414000	15.204519000
H	4.118790000	8.312115000	15.324005000
H	4.273068000	7.746435000	13.662846000
H	5.070902000	6.863709000	14.976642000
C	-0.305805000	3.149369000	18.820320000
C	1.729312000	1.757003000	18.218691000
H	0.383862000	2.698987000	16.854428000
H	0.071962000	3.380234000	19.822124000
H	-0.919579000	2.245250000	18.898142000
H	-0.952855000	3.975899000	18.511478000
H	2.491896000	1.570735000	17.456236000
H	2.239500000	1.945652000	19.169283000
H	1.133466000	0.844279000	18.335890000
O	4.351569000	3.368378000	14.312503000
C	4.818052000	3.065028000	15.659334000
C	6.081012000	3.897049000	15.813932000
C	6.668357000	3.854658000	14.394771000
C	5.425505000	3.938079000	13.510334000
H	5.020094000	1.989700000	15.715119000
H	4.020298000	3.329827000	16.352507000
H	6.754981000	3.490233000	16.571406000
H	5.812447000	4.917779000	16.100984000
H	7.368283000	4.667949000	14.190750000
H	7.188787000	2.905898000	14.225968000
H	5.151431000	4.970287000	13.274926000
H	5.489502000	3.365741000	12.582615000
O	2.425962000	1.204503000	14.677014000
C	1.114567000	0.611289000	14.888660000
C	0.926112000	-0.360544000	13.718286000
C	2.370497000	-0.668724000	13.237888000
C	3.262743000	0.127814000	14.196955000
H	0.388174000	1.421249000	14.921894000
H	1.139446000	0.095616000	15.854906000
H	0.348084000	0.113700000	12.923977000
H	0.392899000	-1.259369000	14.037412000
H	2.609303000	-1.734978000	13.260364000
H	2.509034000	-0.298501000	12.219565000
H	3.574335000	-0.471030000	15.062239000
H	4.140435000	0.578150000	13.732066000

2.14 XYZ Coordinates of **4d**:

Ge	2.925468000	2.795267000	11.075863000
Mg	2.483162000	3.772321000	13.766206000

N	1.076735000	2.299513000	10.980833000
N	0.568638000	3.353852000	12.990780000
N	3.038112000	1.750430000	9.520733000
N	1.448139000	4.525386000	15.368013000
C	1.852498000	1.215105000	9.084298000
C	0.746499000	1.507714000	9.869628000
C	-0.597052000	1.119657000	9.664320000
C	-1.553587000	1.533346000	10.566455000
C	-1.221084000	2.307931000	11.701461000
C	0.128788000	2.671197000	11.920426000
C	-2.180555000	2.750473000	12.689506000
C	-1.763624000	3.434834000	13.787001000
C	-0.377407000	3.727533000	14.002630000
C	0.104094000	4.301380000	15.160715000
C	4.181545000	1.678309000	8.664614000
C	5.207802000	0.756848000	8.940336000
C	6.306004000	0.706265000	8.074563000
C	6.381348000	1.539854000	6.963365000
C	5.359155000	2.450647000	6.708535000
C	4.246719000	2.538977000	7.550330000
C	5.158174000	-0.122802000	10.176975000
C	5.459278000	-1.597708000	9.881103000
C	6.107789000	0.440399000	11.247511000
C	3.185601000	3.603110000	7.316787000
C	2.653905000	3.612801000	5.877887000
C	3.746465000	4.974976000	7.726877000
C	1.882579000	4.943962000	16.638191000
C	1.653032000	4.172969000	17.812909000
C	2.164582000	4.609937000	19.038677000
C	2.917799000	5.774620000	19.145813000
C	3.149329000	6.528609000	17.999579000
C	2.639345000	6.144161000	16.756493000
C	0.917889000	2.840915000	17.774484000
C	2.851531000	7.056501000	15.560267000
C	2.093329000	8.382186000	15.739144000
C	4.338712000	7.305079000	15.268516000
H	1.820153000	0.654830000	8.159183000
H	-0.847382000	0.511660000	8.802982000
H	-2.594379000	1.256830000	10.420175000
H	-3.229517000	2.513258000	12.539728000
H	-2.472930000	3.752146000	14.546924000
H	-0.607701000	4.598230000	15.930534000
H	7.109538000	0.004103000	8.273826000
H	7.238278000	1.484002000	6.298716000
H	5.431182000	3.110468000	5.849556000
H	4.142743000	-0.061706000	10.573156000
H	4.786702000	-1.989404000	9.112707000

H	5.329728000	-2.199556000	10.786843000
H	6.487504000	-1.745103000	9.534723000
H	7.147159000	0.423082000	10.902913000
H	6.046615000	-0.141829000	12.173616000
H	5.843881000	1.479078000	11.468169000
H	2.344207000	3.387953000	7.978733000
H	3.434286000	3.871330000	5.154445000
H	1.852936000	4.351978000	5.775356000
H	2.250805000	2.634094000	5.601313000
H	4.099045000	4.929118000	8.761407000
H	2.973111000	5.747511000	7.657786000
H	4.586769000	5.270312000	7.089034000
H	1.983698000	4.011029000	19.927485000
H	3.313193000	6.091132000	20.106337000
H	3.719844000	7.450961000	18.073506000
H	2.426214000	6.546459000	14.698972000
H	1.028263000	8.196763000	15.906683000
H	2.195855000	9.014237000	14.848715000
H	2.471609000	8.949907000	16.596605000
H	4.847111000	7.782283000	16.113284000
H	4.458379000	7.966337000	14.402144000
H	4.852860000	6.363895000	15.053244000
C	-0.390408000	2.883260000	18.579713000
C	1.819308000	1.690294000	18.252789000
H	0.651525000	2.640694000	16.738425000
H	-0.201382000	3.086010000	19.639488000
H	-0.921499000	1.927202000	18.511167000
H	-1.053409000	3.668660000	18.205288000
H	2.737211000	1.639484000	17.658145000
H	2.110644000	1.812799000	19.301045000
H	1.300977000	0.728443000	18.165164000
O	4.477568000	3.872889000	14.362691000
C	4.787148000	3.461126000	15.736904000
C	6.288791000	3.175457000	15.741365000
C	6.586215000	2.840080000	14.271903000
C	5.670415000	3.811157000	13.542955000
H	4.185578000	2.573861000	15.940064000
H	4.479919000	4.266017000	16.404363000
H	6.545132000	2.364963000	16.427440000
H	6.848461000	4.065560000	16.046252000
H	7.635996000	2.974669000	14.001081000
H	6.301151000	1.809197000	14.042426000
H	6.107684000	4.814799000	13.485547000
H	5.360430000	3.493282000	12.546339000
O	2.627863000	1.626640000	14.361407000
C	1.418531000	0.934901000	14.767991000
C	1.156204000	-0.118731000	13.683591000

C	2.521025000	-0.277083000	12.966573000
C	3.470076000	0.622439000	13.766689000
H	0.631295000	1.677963000	14.862971000
H	1.611803000	0.471413000	15.741449000
H	0.391648000	0.222847000	12.984909000
H	0.811127000	-1.055204000	14.129121000
H	2.873397000	-1.311309000	12.937848000
H	2.441839000	0.091020000	11.942608000
H	3.983147000	0.071863000	14.567795000
H	4.199239000	1.136718000	13.143537000
O	2.708420000	5.586313000	12.629114000
C	3.818069000	6.101300000	11.848613000
H	4.495379000	6.635321000	12.518293000
C	3.197197000	6.992034000	10.764980000
H	3.731555000	6.904394000	9.817736000
H	3.213523000	8.042884000	11.072210000
C	1.751119000	6.480413000	10.702579000
H	1.703546000	5.529223000	10.160225000
H	1.053257000	7.192487000	10.255764000
H	4.329836000	5.240168000	11.409972000
C	1.473242000	6.210229000	12.169851000
H	1.319002000	7.132923000	12.742161000
H	0.667724000	5.508186000	12.369700000

2.15 XYZ Coordinates of **5**:

Ge	7.194097000	0.840246000	0.054409000
Mg	9.395192000	-0.736600000	0.538338000
O	10.771537000	0.081746000	-0.851514000
O	9.929631000	0.816367000	1.956797000
O	8.469437000	-1.758442000	-1.175549000
N	6.209716000	-0.221970000	1.432879000
N	7.948127000	-1.673448000	1.813643000
N	6.173180000	2.242192000	0.869572000
N	10.520061000	-2.342557000	1.272609000
C	4.839266000	1.604093000	0.871115000
C	5.011672000	0.368259000	1.743415000
C	4.208697000	-0.155481000	2.714123000
C	4.653717000	-1.329474000	3.397327000
C	5.884217000	-1.902384000	3.122817000
C	6.716806000	-1.281036000	2.114655000
C	6.449558000	-3.073067000	3.758149000
C	7.706213000	-3.494805000	3.426156000
C	8.494763000	-2.806210000	2.461707000
C	9.802685000	-3.116198000	2.130302000
C	6.340675000	3.627522000	0.944890000
C	7.010165000	4.353291000	-0.083304000

C	7.204205000	5.729634000	0.064788000
C	6.727836000	6.421117000	1.173591000
C	6.062985000	5.716576000	2.170462000
C	5.880994000	4.333619000	2.094040000
C	7.416678000	3.697694000	-1.397638000
C	6.259051000	3.758659000	-2.405978000
C	8.706872000	4.260179000	-2.008320000
C	5.225489000	3.619755000	3.269208000
C	3.714731000	3.907870000	3.345433000
C	5.885302000	3.980947000	4.611648000
C	11.797607000	-2.799830000	0.885345000
C	11.933954000	-3.846619000	-0.058619000
C	13.216231000	-4.241741000	-0.456107000
C	14.351661000	-3.608421000	0.038987000
C	14.212815000	-2.559228000	0.946642000
C	12.951633000	-2.140139000	1.379172000
C	10.704234000	-4.513990000	-0.657725000
C	10.782668000	-4.633625000	-2.187745000
C	10.442480000	-5.889941000	-0.023298000
C	12.791030000	-1.010308000	2.383967000
C	13.909549000	0.036666000	2.325812000
C	12.641989000	-1.572655000	3.808941000
C	5.650316000	0.662357000	-1.194824000
C	4.549796000	1.122029000	-0.569864000
C	3.178825000	1.151667000	-1.097478000
C	2.943259000	1.322618000	-2.473084000
C	1.650017000	1.356905000	-2.980761000
C	0.536320000	1.225919000	-2.140127000
C	0.768766000	1.054016000	-0.771186000
C	2.061688000	1.023665000	-0.255632000
C	-0.865564000	1.301051000	-2.690843000
C	11.807693000	-0.680993000	-1.541827000
C	12.257768000	0.195292000	-2.716040000
C	11.089854000	1.175287000	-2.911188000
C	10.603930000	1.372031000	-1.484193000
C	9.904752000	2.250939000	1.704529000
C	9.241764000	2.896843000	2.928191000
C	8.518570000	1.709827000	3.581275000
C	9.510897000	0.588552000	3.332304000
C	8.398380000	-1.229332000	-2.520272000
C	7.284624000	-2.007754000	-3.238480000
C	7.049108000	-3.219955000	-2.323147000
C	7.332247000	-2.639029000	-0.948017000
H	4.058156000	2.262154000	1.244787000
H	3.267706000	0.316749000	2.973064000
H	4.025109000	-1.771533000	4.165007000
H	5.859499000	-3.601486000	4.500162000

H	8.134923000	-4.370608000	3.907034000
H	10.274882000	-3.985421000	2.589216000
H	7.712278000	6.280473000	-0.719853000
H	6.874329000	7.493466000	1.258287000
H	5.698296000	6.247615000	3.044898000
H	7.606329000	2.635009000	-1.221281000
H	5.344637000	3.351898000	-1.974121000
H	6.497086000	3.178029000	-3.305318000
H	6.068316000	4.794334000	-2.707998000
H	8.580760000	5.287526000	-2.363212000
H	9.006177000	3.659899000	-2.874418000
H	9.528227000	4.257128000	-1.284394000
H	5.364625000	2.546308000	3.126610000
H	3.534926000	4.968216000	3.554559000
H	3.248055000	3.323369000	4.146017000
H	3.201578000	3.672946000	2.408574000
H	6.967211000	3.835893000	4.575950000
H	5.481876000	3.351366000	5.411920000
H	5.698416000	5.023436000	4.889279000
H	13.327127000	-5.050100000	-1.173320000
H	15.339454000	-3.925721000	-0.282094000
H	15.101120000	-2.063251000	1.324292000
H	9.855804000	-3.868757000	-0.423354000
H	11.590423000	-5.299097000	-2.509022000
H	10.949841000	-3.659298000	-2.658707000
H	9.848422000	-5.044498000	-2.585664000
H	9.542863000	-6.350966000	-0.446892000
H	10.301151000	-5.802030000	1.056663000
H	11.284843000	-6.568319000	-0.199231000
H	11.855494000	-0.502987000	2.140964000
H	13.660015000	0.886237000	2.970285000
H	14.053483000	0.414573000	1.307827000
H	14.869550000	-0.359972000	2.674209000
H	11.777395000	-2.237407000	3.875977000
H	12.506798000	-0.762988000	4.535735000
H	13.534052000	-2.139973000	4.097326000
H	5.561648000	0.221514000	-2.182540000
H	3.789969000	1.450439000	-3.140229000
H	1.497722000	1.497110000	-4.048121000
H	-0.076696000	0.938547000	-0.097793000
H	2.208091000	0.860022000	0.806408000
H	-0.925225000	0.867284000	-3.693725000
H	-1.207269000	2.340935000	-2.766739000
H	-1.575454000	0.771457000	-2.049029000
H	11.356581000	-1.622900000	-1.857716000
H	12.604583000	-0.904736000	-0.834120000
H	13.167896000	0.743381000	-2.453532000

H	12.472648000	-0.399134000	-3.607116000
H	10.300066000	0.730539000	-3.523084000
H	11.393657000	2.115131000	-3.377638000
H	11.208540000	2.112699000	-0.948633000
H	9.550789000	1.627144000	-1.387929000
H	9.305496000	2.406268000	0.806491000
H	10.929788000	2.591124000	1.530772000
H	9.998221000	3.303560000	3.607365000
H	8.567958000	3.703682000	2.635869000
H	8.300219000	1.859016000	4.640557000
H	7.588788000	1.507512000	3.043678000
H	10.392042000	0.664525000	3.981347000
H	9.099429000	-0.415813000	3.384133000
H	9.376455000	-1.384142000	-2.981622000
H	8.190312000	-0.155817000	-2.459462000
H	6.376500000	-1.403412000	-3.301263000
H	7.572573000	-2.288529000	-4.254270000
H	7.760081000	-4.020645000	-2.547768000
H	6.036419000	-3.622140000	-2.403184000
H	6.492905000	-2.041872000	-0.576285000
H	7.632372000	-3.359399000	-0.189016000

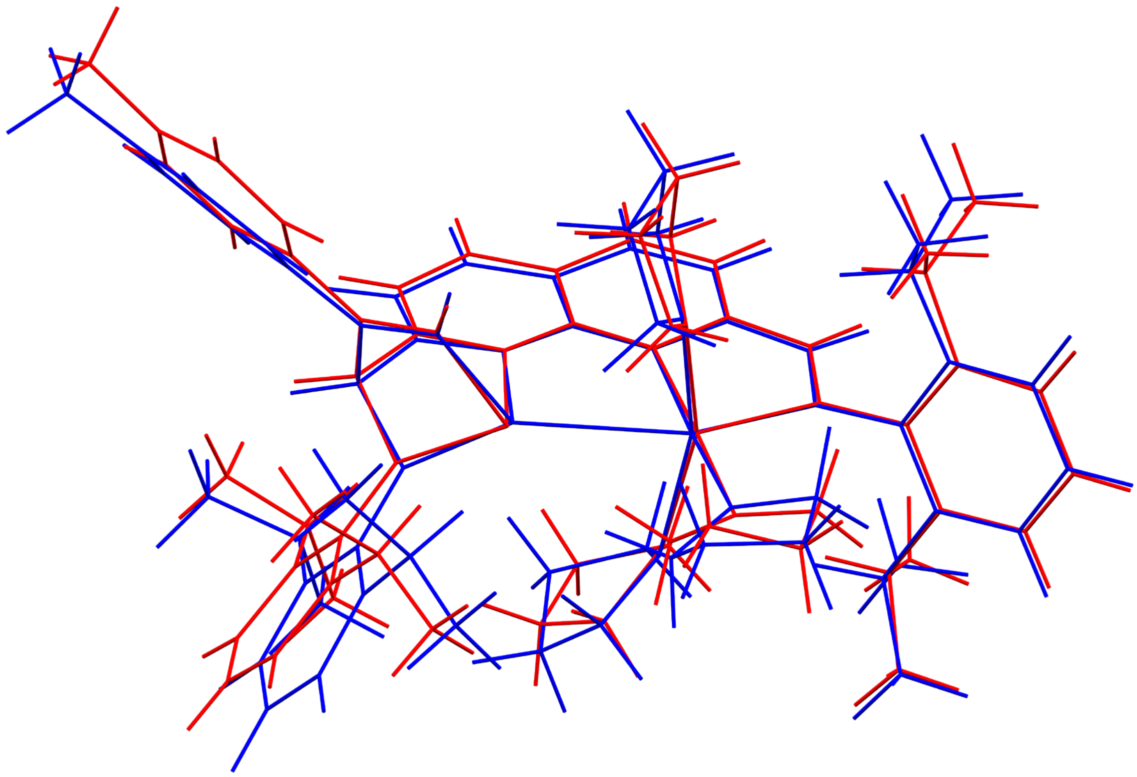

Figure S71: Overlay of gas-phase optimised structure **5** (blue) with the solid-state structure of **5** (red).

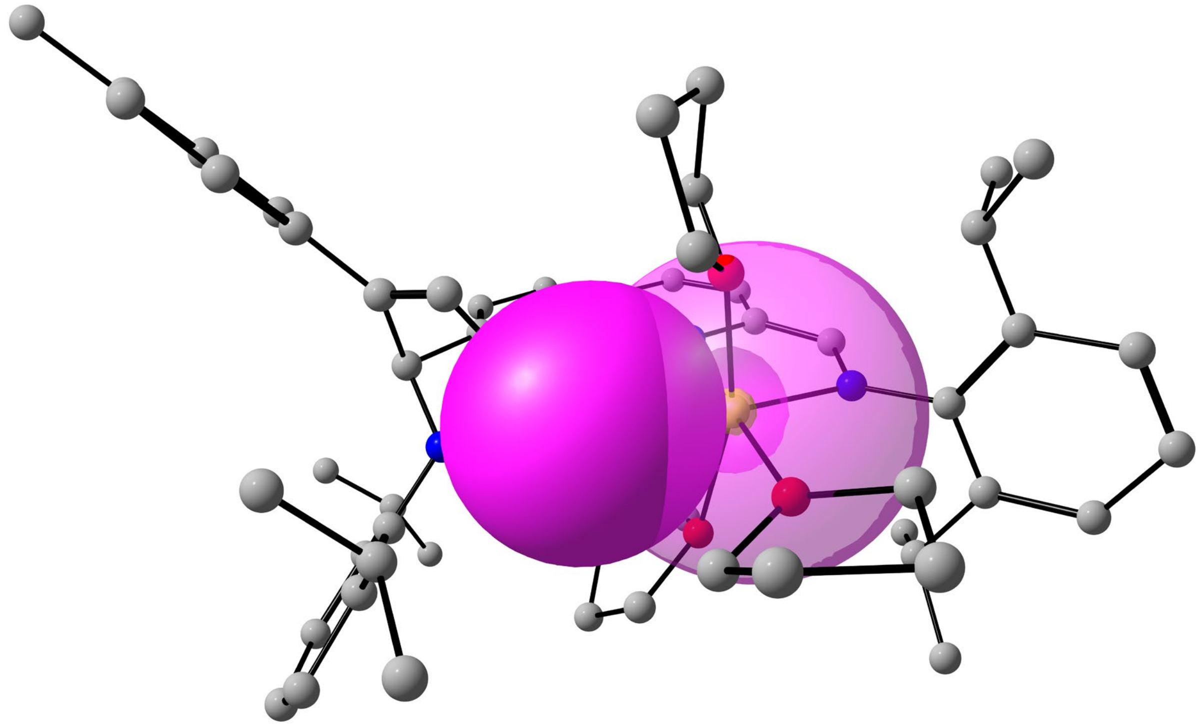

Figure S72: Overlap of the Ge-based lone pair donor NBO (filled) with the Mg-based acceptor NBO (translucent) of **5**.

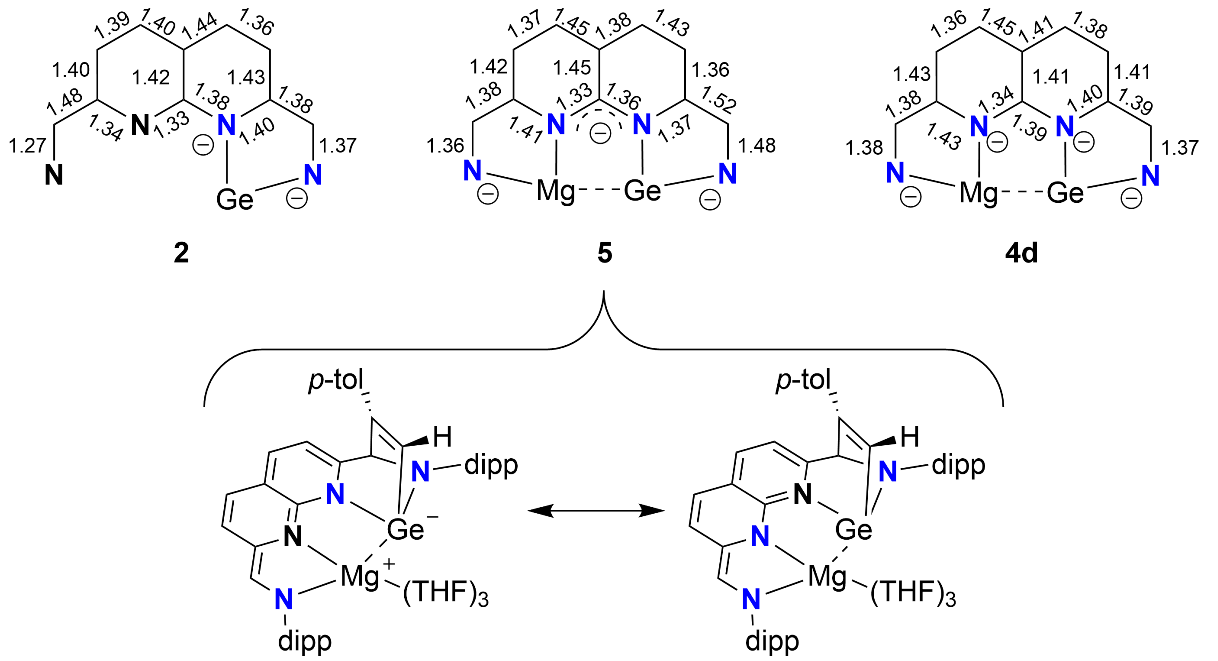

Figure S73: Comparison of the ligand bond metrics of the gas phase optimised structures of **2**, **5**, and **4d**. Two resonance structures are shown of **5**, with the negative charge placed on either of the naphthyridine ring N-donors.

Table S2: Computed energies from the DFT calculations, in hartrees.

Complex	Electronic Energy	Gibbs Thermal Correction
**2**	-3616.35256	0.597697
**2**·*p*-tolylacetylene	-3964.208329	0.726649
p-tolylacetylene	-347.8311965	0.103261
**4d**	-4514.231267	0.940352
**5**	-4862.129449	1.069966

2.16 XYZ Coordinates of **2**·*p*-tolylacetylene:

Ge	7.79982	1.50149	-0.11092
N	6.25080	0.15711	1.01257
N	7.60732	-1.56748	1.65906
N	6.45276	2.72228	0.46746
N	9.85659	-2.97811	1.23926
C	5.18521	2.08622	0.12917
C	5.11014	0.82132	1.00383
C	3.99457	0.38866	1.75581
C	4.11486	-0.74063	2.53817
C	5.33444	-1.45894	2.55415
C	6.40402	-0.97258	1.73808
C	5.59751	-2.61344	3.33174
C	6.83719	-3.20413	3.25968
C	7.81761	-2.65445	2.38602
C	9.16695	-3.25677	2.27021
C	6.42447	3.74501	1.44123
C	5.83425	4.98262	1.07508
C	5.72455	5.99656	2.02821
C	6.22088	5.82663	3.31792
C	6.84367	4.63061	3.65701
C	6.94962	3.57474	2.74395
C	5.37971	5.23864	-0.35556
C	3.86917	5.50449	-0.45429
C	6.18110	6.39112	-0.98439
C	7.66250	2.30365	3.17832
C	7.03350	1.66953	4.42778
C	9.16525	2.56625	3.35777
C	11.19359	-3.29744	1.00500
C	11.77407	-4.57346	1.19119
C	13.13467	-4.71847	0.89738
C	13.89669	-3.65028	0.43619
C	13.30008	-2.40826	0.21776
C	11.94428	-2.21275	0.47067
C	10.97060	-5.78561	1.64111
C	11.15602	-6.98463	0.69594
C	11.31318	-6.17592	3.08937
C	11.25644	-0.87315	0.25520
C	11.89289	-0.01248	-0.84124
C	11.16702	-0.07944	1.57160
C	6.53054	1.22041	-1.63874
C	5.27583	1.59532	-1.32195
C	4.06217	1.50385	-2.14886
C	4.15317	1.26356	-3.53378
C	3.01912	1.17336	-4.32647
C	1.73374	1.31515	-3.78026

C	1.63666	1.55116	-2.40775
C	2.77469	1.64783	-1.60806
C	0.50899	1.22822	-4.65500
H	4.33003	2.73495	0.32526
H	3.07235	0.95767	1.72938
H	3.28473	-1.08402	3.14923
H	4.82045	-3.01542	3.97548
H	7.07442	-4.08553	3.84654
H	9.52164	-3.85757	3.11856
H	5.26529	6.94114	1.75003
H	6.13613	6.62603	4.04813
H	7.25024	4.50608	4.65664
H	5.61126	4.34295	-0.93530
H	3.28473	4.67877	-0.03590
H	3.56680	5.63132	-1.49903
H	3.58920	6.41365	0.08931
H	6.00349	7.33783	-0.46297
H	5.89811	6.52830	-2.03373
H	7.25399	6.18176	-0.94292
H	7.57144	1.56677	2.38222
H	7.11834	2.31836	5.30575
H	7.53222	0.72322	4.66477
H	5.97024	1.46371	4.26638
H	9.59130	2.94959	2.42548
H	9.69269	1.64356	3.61968
H	9.35296	3.30372	4.14561
H	13.60429	-5.68800	1.03162
H	14.95348	-3.78926	0.22865
H	13.89577	-1.58793	-0.16721
H	9.90991	-5.52191	1.60575
H	12.18225	-7.36404	0.71695
H	10.92069	-6.71023	-0.33613
H	10.49618	-7.80681	0.99232
H	10.70295	-7.02320	3.42062
H	11.15034	-5.34281	3.78048
H	12.36596	-6.46553	3.17287
H	10.22372	-1.09246	-0.03644
H	11.25414	0.85629	-1.02962
H	12.00368	-0.56744	-1.77826
H	12.88026	0.36435	-0.55064
H	10.64444	-0.65127	2.34249
H	10.60774	0.84448	1.40618
H	12.16579	0.17310	1.94590
H	6.76843	0.78542	-2.60536
H	5.13208	1.15950	-3.98914
H	3.12729	0.99294	-5.39313
H	0.65565	1.66435	-1.95381

H	2.64716	1.83131	-0.54730
H	0.50420	0.30464	-5.24425
H	0.46800	2.06202	-5.36576
H	-0.40828	1.25315	-4.06079

2.17 XYZ Coordinates of *p*-tolylacetylene:

C	-1.98169	-1.08815	1.30757
C	-2.79028	-1.25694	2.19182
H	-3.50193	-1.40548	2.97005
C	0.86197	-0.49150	-1.80157
C	-0.50654	-0.58532	-2.08561
C	1.26767	-0.59550	-0.46481
H	-0.84392	-0.50105	-3.11509
H	2.32389	-0.51922	-0.22122
C	-1.44090	-0.78211	-1.07519
C	0.34442	-0.79235	0.55573
H	-2.49769	-0.85058	-1.30986
H	0.67299	-0.86877	1.58663
C	-1.02761	-0.88954	0.26435
C	1.87219	-0.31129	-2.90624
H	2.21711	-1.28085	-3.28633
H	1.44631	0.23647	-3.75169
H	2.75399	0.23302	-2.55639

3. Crystallographic Information:

3.1 X-ray Crystal Structure Determination of ^{dipp}**NBA**:

$C_{34}H_{40}N_4$ + disordered solvent, Fw = 504.70[*], colourless plate, 0.47 × 0.20 × 0.06 mm^3, monoclinic, $P2_1/n$ (no. 14), a = 8.9749(8), b = 15.2114(12), c = 23.800(2) Å, β = 96.267(4) °, V = 3229.7(5) Å^3, Z = 4, D_x = 1.038 g/cm^3[*], μ = 0.06 mm^{-1}[*]. The diffraction experiment was performed on a Bruker Kappa ApexII diffractometer with sealed tube and Triumph monochromator (λ = 0.71073 Å) at a temperature of 100(2) K up to a resolution of $(\sin \theta/\lambda)_{max}$ = 0.58 Å^{-1}. The Eval15 software[20] was used for the intensity integration. A multi-scan absorption correction and scaling was performed with SADABS[21] (correction range 0.63-0.75). A total of 38413 reflections was measured, 5202 reflections were unique (R_{int} = 0.116), 3093 reflections were observed [I>2σ(I)]. The structure was solved with Patterson superposition methods using SHELXT.[22] Structure refinement was performed with SHELXL-2018[23] on F^2 of all reflections. The crystal structure contains channels (372 Å^3 / unit cell) filled with severely disordered *n*-hexane molecules. Their contribution to the structure factors was secured by back-Fourier transformation using the SQUEEZE algorithm[24] resulting in 97 electrons / unit cell. Non-hydrogen atoms were refined freely with anisotropic displacement parameters. Hydrogen atoms were introduced in calculated positions and refined with a riding model. 351 Parameters were refined with no restraints. R1/wR2 [I > 2σ(I)]: 0.0539 / 0.1125. R1/wR2 [all refl.]: 0.1101 / 0.1326. S = 1.025. Residual electron density between -0.21 and 0.33 e/Å^3. Geometry calculations and checking for higher symmetry was performed with the PLATON program.[25]

[*] Derived values do not contain the contribution of the disordered solvent molecules.

3.2 X-ray Crystal Structure Determination of (^{dipp}**NBA***Mg)$_2$ (**1**):

$C_{68}H_{80}MgN_8$, Fw = 1058.02, dark red needle, 0.37 × 0.11 × 0.06 mm^3, triclinic, P1 (no. 1), a = 9.5033(5), b = 11.6436(4), c = 14.6882(7) Å, α = 79.969(2), β = 89.660(3), γ = 69.433(2) °, V = 1495.75(12) Å^3, Z = 1, D_x = 1.175 g/cm^3, μ = 0.09 mm^{-1}. The diffraction experiment was performed on a Bruker Kappa ApexII diffractometer with sealed tube and Triumph monochromator (λ = 0.71073 Å) at a temperature of 150(2) K up to a resolution of $(\sin \theta/\lambda)_{max}$ = 0.61 Å^{-1}. Intensity integration was performed with the Eval15 software[20]. A multi-scan absorption correction and scaling was performed with SADABS[21] (correction range 0.58-0.75). A total of 22437 reflections was measured, 10987 reflections were unique (R_{int} = 0.058), 7491 reflections were observed [I>2σ(I)]. The structure was solved with Patterson superposition methods using SHELXT[22]. Structure refinement was performed with SHELXL-2018[23] on F^2 of all reflections. Non-hydrogen atoms were refined freely with anisotropic displacement parameters. One of the *i*-propyl groups was refined with a disorder model. Hydrogen atoms were introduced in calculated positions and refined with a riding model. 740 Parameters were refined with 1114 restraints (floating origin, distances and angles of the *i*-propyl groups, displacement parameters of all atoms). R1/wR2 [I > 2σ(I)]: 0.0751 / 0.1705. R1/wR2 [all refl.]: 0.1189 / 0.1953. S = 1.019. Because of the very weak diffraction and the absence of strong anomalous scatterers, the absolute structure could not be determined reliably. Residual electron density between -0.38 and 0.95 e/Å^3. Geometry calculations and checking for higher symmetry was performed with the PLATON program.[25]

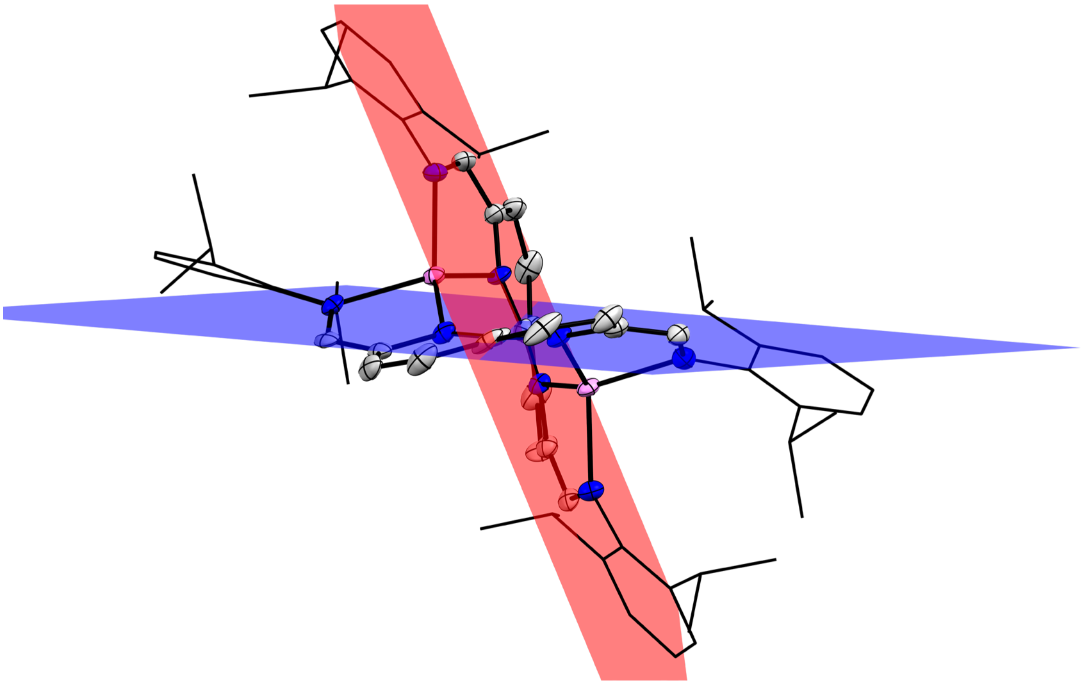

Figure S74: Dihedral angle of 67.9(10) ° between the two monomeric units of **1**, planes defined by N11–C61–N21 and N12–C62–N22.

Table S3: Comparison of bond lengths in the solid-state of the free **^{dipp}NBA** ligand and the two monomeric units in **1**. Bond lengths are in Å.

**Bond**	**^{dipp}NBA**	**1**-1	**1**-2
**C1–C2**	1.471(3)	1.383(10)	1.385(10)
**C9–C10**	1.478(3)	1.387(9)	1.409(9)
**C1–N3**	1.270(3)	1.324(9)	1.331(9)
**C2–N1**	1.332(3)	1.399(8)	1.413(8)
**C10–N4**	1.263(3)	1.321(9)	1.301(9)
**C9–N2**	1.327(3)	1.401(9)	1.400(9)

3.3 X-ray Crystal Structure Determination of ^{dipp}NBA*GeZnCl$_2$ (3):

$C_{34}H_{40}Cl_2GeN_4Zn \cdot 5C_4H_8O_2$, Fw = 1154.08, green needle, 0.71 × 0.22 × 0.06 mm^3, triclinic, P $\overline{1}$ (no. 2), a = 9.4888(4), b = 15.8244(6), c = 20.8674(7) Å, α = 95.401(1), β = 101.085(1), γ = 101.331(1) °, V = 2986.5(2) Å^3, Z = 2, D_x = 1.283 g/cm^3, μ = 1.05 mm^{-1}. The diffraction experiment was performed on a Bruker Kappa ApexII diffractometer with sealed tube and Triumph monochromator (λ = 0.71073 Å) at a temperature of 150(2) K up to a resolution of $(\sin\theta/\lambda)_{max}$ = 0.65 Å^{-1}. Intensity integration was performed with the Eval15 software.[20] The integration ignored the diffuse scattering which was significantly present in the diffraction pattern. A multi-scan absorption correction and scaling was performed with SADABS[21] (correction range 0.54-0.75). A total of 62546 reflections was measured, 13729 reflections were unique (R_{int} = 0.063), 9078 reflections were observed [I>2σ(I)]. The structure was solved with Patterson superposition methods using SHELXT[22]. Structure refinement was performed with SHELXL-2019[23] on F^2 of all reflections. Non-hydrogen atoms were refined freely with anisotropic displacement parameters. One of the *i*-propyl groups of the metal complex was refined with a disorder model. The non-coordinated 1,4-dioxane solvent molecules were refined with very approximate disorder models. Hydrogen atoms were introduced in calculated positions and refined with a riding model. 1006 Parameters were refined with 3294

restraints (distances, angles and displacement parameters of the *i*-propyl groups and solvent molecules). R1/wR2 [I > 2σ(I)]: 0.0788 / 0.2215. R1/wR2 [all refl.]: 0.1155 / 0.2410. S = 1.043. Residual electron density between -1.05 and 1.11 e/Å^3. Geometry calculations and checking for higher symmetry was performed with the PLATON program.[25]

Table S4: Selected bond distances and angles found in **3**, in Å and ° respectively.

Distances (Å)		Angles (°)	
**Ge1–Zn1**	3.1110(8)	**N1–Ge1–N3**	84.15(17)
**Ge1–N1**	1.920(4)	**N1–Ge1–Zn1**	83.71(12)
**Ge1–N3**	1.850(4)	**N3–Ge1–Zn1**	164.35(13)
**Zn1–Cl1**	2.2142(16)	**Ge1–Zn1–Cl1**	81.06(5)
**Zn1–Cl2**	2.2207(15)	**Ge1–Zn1–Cl2**	81.90(4)
**Zn1–N2**	2.138(4)	**Ge1–Zn1–N2**	73.23(10)
**Zn1–N4**	2.065(4)	**Ge1–Zn1–N4**	151.77(12)
**N1–C2**	1.410(6)	**Cl1–Zn1–Cl2**	121.04(7)
**N1–C6**	1.381(6)	**Cl1–Zn1–N2**	102.10(12)
**N2–C6**	1.330(6)	**Cl1–Zn1–N4**	116.78(14)
**N2–C9**	1.381(6)	**Cl2–Zn1–N2**	125.58(12)
**N3–C1**	1.378(6)	**Cl2–Zn1–N4**	103.98(13)
**N4–C10**	1.283(6)	**N2–Zn1–N4**	81.31(16)
**C1–C2**	1.361(7)		
**C2–C3**	1.422(7)		
**C3–C4**	1.349(7)		
**C4–C5**	1.434(7)		
**C5–C6**	1.443(6)		
**C5–C7**	1.386(7)		
**C7–C8**	1.373(7)		
**C8–C9**	1.383(7)		
**C9–C10**	1.452(7)		

3.4 X-ray Crystal Structure Determination of ^{dipp}**NBA***GeMg·*p*-tolylacetylene (**5**):

$C_{55}H_{72}GeMgN_4O_3$ + disordered solvent, Fw = 934.06[*], green-brown needle, 0.54 × 0.13 × 0.10 mm^3, tetragonal, P4/ncc (no. 130), a = b = 33.8763(7), c = 18.7137(4) Å, V = 21475.8(10) Å^3, Z = 16, D_x = 1.156 g/cm^3[*], μ = 0.63 mm^{-1}[*]. The diffraction experiment was performed on a Bruker Kappa ApexII diffractometer with sealed tube and Triumph monochromator (λ = 0.71073 Å) at a temperature of 150(2) K up to a resolution of $(\sin \theta/\lambda)_{max}$ = 0.58 Å^{-1}. The Eval15 software[20] was used for the intensity integration. A numerical absorption correction and scaling was performed with SADABS[21] (correction range 0.62-1.00). A total of 189564 reflections was measured, 8936 reflections were unique (R_{int} = 0.157), 5291 reflections were observed [I>2σ(I)]. The structure was solved with Patterson superposition methods using SHELXT.[22] Structure refinement was performed with SHELXL-2019[23] on F^2 of all reflections. The crystal structure contains solvent channels along the c-axis (2204 Å^3 / unit cell). There are two symmetry independent channel types. We assume that the channel with fourfold rotation symmetry (169 Å^3 / channel) is filled with disordered THF molecules, and the channel with fourfold rotoinversion symmetry (328 Å^3 / channel) with disordered methyl-tert-butyl

ether. The molecules in the solvent channels were treated as diffuse electron density using the SQUEEZE algorithm[24] resulting in 348 electrons / unit cell. Non-hydrogen atoms were refined freely with anisotropic displacement parameters. One coordinated THF molecule was refined with puckering disorder. Hydrogen atoms of the coordinated THF molecules were introduced in calculated positions. All other hydrogen atoms were located in difference electron maps. Hydrogen atoms were constrained in the refinement. 605 Parameters were refined with 246 restraints (geometries and displacement parameters of the coordinated THF). R1/wR2 [I > 2σ(I)]: 0.0587 / 0.1424. R1/wR2 [all refl.]: 0.1125 / 0.1709. S = 1.026. Residual electron density between -0.42 and 0.61 e/Å^3. Geometry calculations and checking for higher symmetry was performed with the PLATON program.[25]

[*] Derived values do not contain the contribution of the disordered solvent molecules.

Table S5: Naphthyridine bond lengths in the solid-state of **5**.

Bond	Distance (Å)
**C1–C2**	1.511(6)
**C2–C3**	1.349(6)
**C3–C4**	1.414(7)
**C4–C5**	1.379(6)
**C5–C6**	1.438(6)
**C5–C7**	1.426(6)
**C7–C8**	1.368(6)
**C8–C9**	1.415(6)
**C9–C10**	1.380(6)
**N1–C2**	1.385(5)
**N1–C6**	1.354(5)
**N2–C6**	1.332(5)
**N2–C9**	1.418(5)

CCDC 2427530-2427533 contain the supplementary crystallographic data for this paper. These data can be obtained free of charge from The Cambridge Crystallographic Data Centre via www.ccdc.cam.ac.uk/data_request/cif.

4. References:

[1] D. J. Shields, T. Elkoush, E. Miura-Stempel, C. L. Mak, G.-H. Niu, A. D. Gudmundsdottir, M. G. Campbell, *Inorg. Chem.* **2020**, *59*, 18338–18344.

[2] M. J. Behlen, C. Uyeda, *J. Am. Chem. Soc.* **2020**, *142*, 17294–17300.

[3] Frisch, M. J.; Trucks, G. W.; Schlegel, H. B.; Scuseria, G. E.; Robb, M. A.; Cheeseman, J. R.; Scalmani, G.; Barone, V.; Petersson, G. A.; Nakatsuji, H.; Li, X.; Caricato, M.; Marenich, A. V.; Bloino, J.; Janesko, B. G.; Gomperts, R.; Mennucci, B.; Hratchian, H. P.; Ortiz, J. V.; Izmaylov, A. F.; Sonnenberg, J. L.; Williams; Ding, F.; Lipparini, F.; Egidi, F.; Goings, J.; Peng, B.; Petrone, A.; Henderson, T.; Ranasinghe, D.; Zakrzewski, V. G.; Gao, J.; Rega, N.; Zheng, G.; Liang, W.; Hada, M.; Ehara, M.; Toyota, K.; Fukuda, R.; Hasegawa, J.; Ishida, M.; Nakajima, T.; Honda, Y.; Kitao, O.; Nakai, H.; Vreven, T.; Throssell, K.; Montgomery Jr., J. A.; Peralta, J. E.; Ogliaro, F.; Bearpark, M. J.; Heyd, J. J.; Brothers, E. N.; Kudin, K. N.; Staroverov, V. N.; Keith, T. A.; Kobayashi, R.; Normand, J.; Raghavachari, K.; Rendell, A. P.; Burant, J. C.; Iyengar, S. S.; Tomasi, J.; Cossi, M.; Millam, J. M.; Klene, M.; Adamo, C.; Cammi, R.; Ochterski, J. W.; Martin, R. L.; Morokuma, K.; Farkas, O.; Foresman, J. B.; Fox, D. J. Gaussian 16 Rev. C.02, Wallingford, CT, (2016)

[4] A. D. Becke, *J. Chem. Phys.* **1993**, *98*, 5648–5652.

[5] C. Lee, W. Yang, R. G. Parr, *Phys. Rev. B* **1988**, *37*, 785–789.

[6] R. Ditchfield, W. J. Hehre, J. A. Pople, *J. Chem. Phys.* **2003**, *54*, 724–728.

[7] M. S. Gordon, J. S. Binkley, J. A. Pople, W. J. Pietro, W. J. Hehre, *J. Am. Chem. Soc.* **1982**, *104*, 2797–2803.

[8] M. M. Francl, W. J. Pietro, W. J. Hehre, J. S. Binkley, M. S. Gordon, D. J. DeFrees, J. A. Pople, *J. Chem. Phys.* **1982**, *77*, 3654–3665.

[9] P. C. Hariharan, J. A. Pople, *Theoret. Chim. Acta* **1973**, *28*, 213–222.

[10] W. J. Hehre, R. Ditchfield, J. A. Pople, *J. Chem. Phys.* **2003**, *56*, 2257–2261.

[11] R. Krishnan, J. S. Binkley, R. Seeger, J. A. Pople, *J. Chem. Phys.* **2008**, *72*, 650–654.

[12] A. D. McLean, G. S. Chandler, *J. Chem. Phys.* **1980**, *72*, 5639–5648.

[13] S. Grimme, J. Antony, S. Ehrlich, H. Krieg, *J. Chem. Phys.* **2010**, *132*, 154104.

[14] S. Grimme, S. Ehrlich, L. Goerigk, *J. Comput. Chem.* **2011**, *32*, 1456–1465.

[15] J. P. Foster, F. Weinhold, *J. Am. Chem. Soc.* **1980**, *102*, 7211–7218.

[16] NBO 7.0. E. D. Glendening, J, K. Badenhoop, A. E. Reed, J. E. Carpenter, J. A. Bohmann, C. M. Morales, P. Karafiloglou, C. R. Landis, and F. Weinhold, Theoretical Chemistry Institute, University of Wisconsin, Madison (2018)

[17] A. E. Reed, F. Weinhold, *J. Chem. Phys.* **1983**, *78*, 4066–4073.

[18] A. E. Reed, R. B. Weinstock, F. Weinhold, *J. Chem. Phys.* **1985**, *83*, 735–746.

[19] K. B. Wiberg, *Tetrahedron* **1968**, *24*, 1083–1096.

[20] A. M. M. Schreurs, X. Xian, L. M. J. Kroon-Batenburg, *J. Appl. Cryst.* **2010**, *43*, 70–82.

[21] L. Krause, R. Herbst-Irmer, G. M. Sheldrick, D. Stalke, *J. Appl. Cryst.* **2015**, *48*, 3–10.

[22] G. M. Sheldrick, *Acta Cryst. A* **2015**, *71*, 3–8.

[23] G. M. Sheldrick, *Acta Cryst. C* **2015**, *71*, 3–8.

[24] A. L. Spek, *Acta Cryst. C* **2015**, *71*, 9–18.

[25] A. L. Spek, *Acta Cryst. D* **2009**, *65*, 148–155.
